# Human microglia clear intraneuronal α-synuclein aggregates by GPNMB-mediated trogocytosis

**DOI:** 10.1126/scitranslmed.adz9258

**Published:** 2026-10-07

**Authors:** Hung-Ju Chueh, Antigoni Katsikoudi, Ana Aragón-González, Chor Lai Lam, Liezel Tamon, Ashwin Jainarayanan, Devika Agarwal, Sally A. Cowley, David Sims, George K Tofaris

**Affiliations:** 1 Nuffield Department of Clinical Neurosciences, University of Oxford, Oxford, UK; 2 Kavli Institute for Nanoscience Discovery, University of Oxford, Oxford, UK; 3 MRC Weatherall Institute of Molecular Medicine, University of Oxford, Oxford, UK; 4 Kennedy Institute of Rheumatology, University of Oxford, Oxford, UK; 5 James and Lillian Martin Centre for Stem Cell Research, Sir William Dunn School of Pathology, University of Oxford, Oxford, UK

## Abstract

Microglia are the primary immune cells of the brain, but their role in Parkinson’s disease is not fully understood. Chronic microglial activation is toxic to neurons, but in the early stages of pathology they also exert beneficial functions. We used induced pluripotent stem cell (iPSC)–derived co-culture models to investigate how human microglia respond to α-synuclein aggregates that form de novo inside human dopaminergic neurons with α-synuclein gene triplication or are triggered by fibrils. We found that microglia cleared Ser129-phosphorylated α-synuclein aggregates through a contact-dependent mechanism. This process was fine-tuned by sensing (P2RY12) and inhibitory (CD22) signals and involved selective phagocytosis of neuronal sub-compartments (trogocytosis), independently of TREM2 or phosphatidylserine. In the presence of intraneuronal α-synuclein aggregates, microglia exhibited morphological and transcriptional changes indicative of activation. Using single cell sequencing, we identified a cluster of disease-associated microglia (DAM) responsible for this beneficial phenotype and the Parkinson’s disease GWAS candidate Glycoprotein non-metastatic melanoma protein B (GPNMB*)* as a key effector of aggregate clearance by microglial lysosomes. GPNMB interacted with Ser129-phosphorylated α-synuclein in iPSC-derived microglia exposed to aggregate-laden dopaminergic neurons and was upregulated in substantia nigra microglia of individuals with incidental Lewy bodies or Parkinson’s disease. Microglia-specific GPNMB knockdown using CRISPRi reduced intraneuronal aggregate clearance. GPNMB-mediated trogocytosis was negatively regulated by an autocrine IL-10 signalling loop, whereas IL-10 receptor blockade enhanced the phagocytic response. Together, our study identifies a subtype of human microglia capable of removing intraneuronal aggregates and potential therapeutic targets.

## Introduction

Parkinson’s disease (PD) is a neurodegenerative disorder currently without a cure, affecting more than 1% of the global population over the age of 60. Pathologically, it is characterised by the degeneration of primarily dopaminergic but also other types of neurons and formation of intraneuronal filamentous inclusions composed of α-synuclein. The identification of families with α-synuclein gene (*SNCA*) multiplications indicates that an increase in wild-type α-synuclein levels through a gene dosage effect is sufficient to cause PD, suggesting a causal relationship between α-synuclein accumulation, aggregation and proteotoxicity (1). Therefore, understanding the mechanisms that regulate the clearance of α-synuclein aggregates is key to the development of therapies that prevent the spread of pathology and progression of the clinical symptoms. So far, most studies have focused on intracellular clearance pathways (2), which are already compromised by protein aggregates (3), whereas the contribution of non-cell-autonomous mechanisms is not fully understood or largely ascribed to the clearance of aggregates after their release from neurons (4, 5).

Microglia are the specialised brain resident scavenging cells that survey its microenvironment, provide neurotrophic support, prune synapses, and remove cellular debris and pathogenic entities. Although activated microglia bind to and internalise pathogenic proteins such as α-synuclein via pattern recognition receptors facilitating their clearance (6, 7), persistent activation by these stimuli exacerbates neuroinflammation and neurodegeneration (8, 9). Age-dependent changes in microglia also influence their phagocytic efficiency such as the up-regulation of the sialic acid–binding receptor CD22, which when blocked restores microglial homeostasis and improves cognitive function in aged mice (10). Such studies underscore the therapeutic potential of targeting selective microglial receptors. Transcriptomic profiling of microglia has identified a subpopulation known as disease-associated microglia (DAM), that are characterised by the upregulation of genes implicated in lysosomal, phagocytic, and lipid metabolism pathways (11, 12). Depending on the stimulus, microglia transition into diverse DAM states with sub-specialised functions (13, 14) some of which are beneficial, at least in the early phase of the neurodegenerative process (15). Deciphering these activation states is confounded by the realisation that microglial responses in humans differ from rodents (16) and exhibit a greater transcriptomic heterogeneity (17).

This complexity of microglia states calls for models that involve human cells in an environment that resembles the relevant disease pathology. Here, we have modelled the interaction of microglia with dopaminergic neurons using human induced pluripotent stem cell (iPSC)-derived co-cultures under pathological conditions of intraneuronal α-synuclein aggregation to determine whether microglia can clear aggregates and to identify the cellular and molecular pathways that govern this response.

## Results

### Human iPSC-based modelling of long-term interactions between microglia and dopaminergic neurons reveals a protective response.

Most studies to date have focused on short-term interactions between microglia and neurons, typically over 48-72 hrs often with LPS-primed microglia (18), or on the acute microglial response to fibrils (19). To investigate the effects of healthy human microglia in a chronic model of intraneuronal α-synuclein pathology, we initially co-cultured iPSC-derived microglia (MG) from two control lines with dopaminergic neurons (DANs) from a patient with *SNCA* triplication (SNCA^TRIP^, DAN^TRIP^; table S1). DAN^TRIP^ were differentiated as we described previously (20) and their identity was confirmed by immunostaining (83% TUJ1^+^ MAP2^+^, 72% TH^+^) as shown in fig. S1A. MG were generated by a MYB-independent iPSC differentiation protocol (21). Immunofluorescence showed that 94% of cells express IBA1 and 97% express TMEM119 (fig. S1B).

**Fig. 1:**
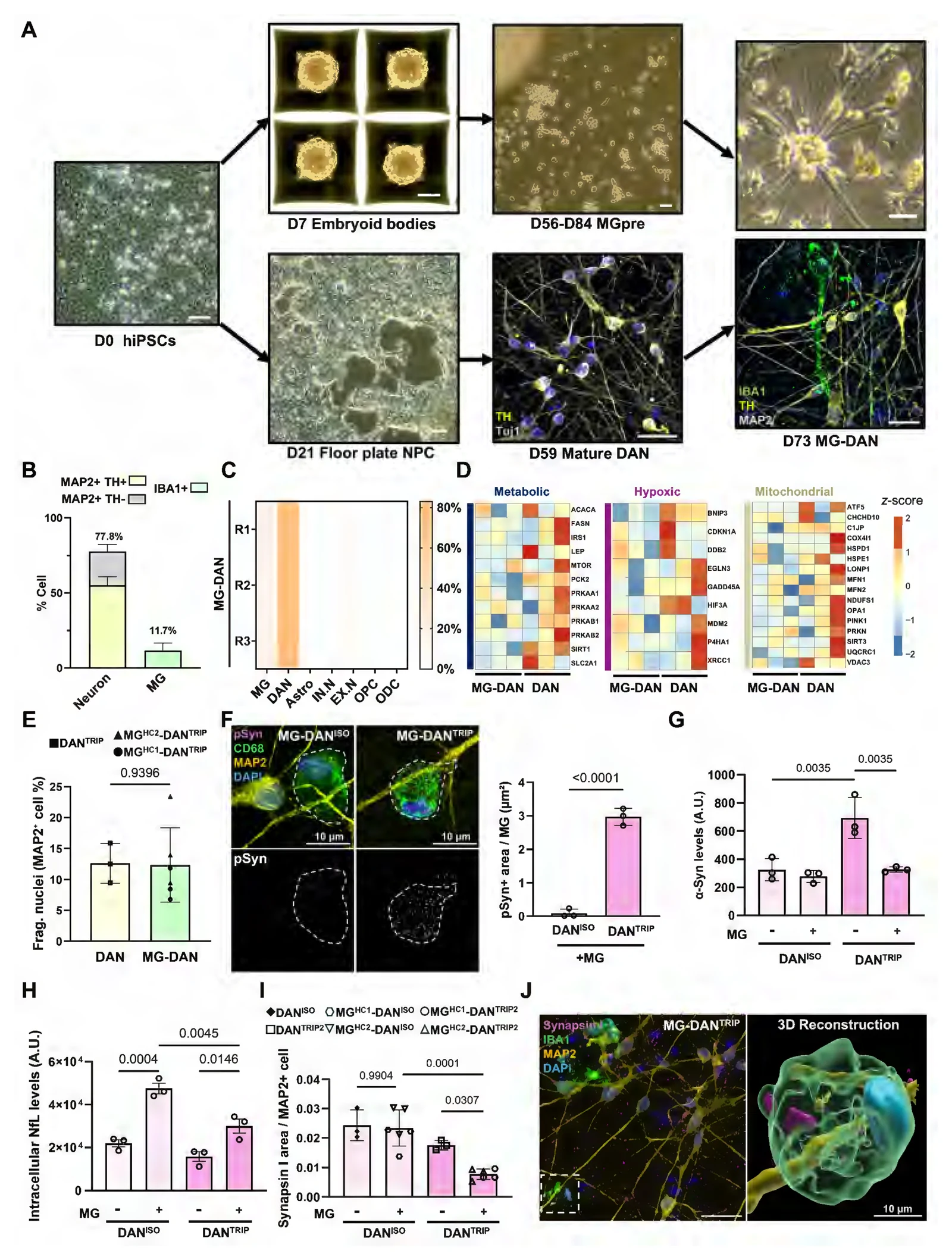
Human iPSC-based modelling of long-term interactions between microglia and dopaminergic neurons reveals a protective response. **(A)** Schematic illustration of the co-culture model. Macrophage precursors (MGpre), generated from iPSC-derived embryoid bodies, were added to DAN cultures at D59 and differentiated into microglia (MG) during two weeks of co-culture. Transcriptomic analysis, immunofluorescence and all cellular assays were performed at D73. Immunostaining confirmed the presence of IBA1^+^ MG and TH^+^ DANs in co-cultures. **(B)** Immunostaining quantification showing the percentage of microglia (IBA1^+^) and the percentage of DAN (TH^+^) and other MAP2^+^ neurons in the co-culture (n = 3 independent differentiations for MG^HC1^-DAN^TRIP1^). **(C)** Heatmap showing cell-type deconvolution of bulk RNA-seq data using the MuSiC algorithm. Relative proportions (%) of microglia (MG), dopaminergic neurons (DAN), astrocytes (Astro), inhibitory neurons (IN.N), excitatory neurons (EX.N), oligodendrocyte precursor cells (OPC), and oligodendrocytes (ODC) in the co-culture. R1–R3 denote three independent differentiations for MG^HC1^-DAN^TRIP1^. **(D)** Bulk RNA-seq heatmaps for metabolic, oxidative, and mitochondrial stress signatures in MG^HC1^-DAN^TRIP1^ co-cultures compared to DAN^TRIP1^ monocultures. **(E)** Quantification of the percentage of MAP2^+^ cells with fragmented nuclei in DAN^TRIP1^ monocultures versus MG^HC1^- DAN^TRIP1^ co-cultures (n=3 independent differentiations) **(F)** Representative instant structured illumination microscopy (iSIM) images and quantification showing pSyn ^+^ puncta within CD68^+^ MG^HC^ co-cultured with DAN^TRIP^ or DANISO (n = 3 independent differentiation for MG^HC1^-DAN^ISO^ and MG^HC1^-DAN^TRIP2^). **(G)** Quantification of total α-synuclein by ELISA in cell lysates from DAN monocultures and MG-DAN co-cultures (n = 3 independent differentiations for DAN^ISO^, DAN^TRIP2^, MG^HC1^-DAN^ISO^ and MG^HC1^-DAN^TRIP2^). **(H)** Quantification of NfL by MSD electrochemiluminescence in cell lysates from DAN monocultures and MG-DAN co-cultures (n = 3 independent differentiations for DAN^ISO^, DAN^TRIP2^, MG^HC1^-DAN^ISO^ and MG^HC1^-DAN^TRIP2^). **(I)** Quantification of total Synapsin I area in DAN monocultures and MG-DAN co-cultures (n = 3 independent differentiations for DAN^ISO^, DAN^TRIP2^, MG^HC1^-DAN^ISO^ and MG^HC1^-DAN^TRIP2^). **(J)** Representative 3D reconstruction from confocal images showing Synapsin I^+^ and MAP2^+^ puncta within IBA1^+^MG co-cultured with DAN^TRIP^. All graphs show mean ± SD and were analysed by two-tailed t-test (**E, F**) or one-way ANOVA (**G, H, I**) followed by Tukey’s post hoc test. *Scale bar*, 20 μm unless otherwise specified.

Harvested microglia precursors (MGpre) were added to DANs at 1:1 ratio and co-cultured for two weeks from D59 until D73, a period during which further maturation of both cell types was achieved (Fig. 1A and fig. S1C). Quantification by immunofluorescence confocal microscopy at D73 revealed that the co-culture consisted of 11.7% IBA1^+^ MG and 78.8% of MAP2^+^ neurons, of which 71.1% were TH^+^ (Fig. 1B). The composition of the co-culture was further assessed transcriptomically using multiple cell markers (Fig. 1C). This was achieved by cell type deconvolution analysis of bulk RNASeq with a reference multi-subject human single cell atlas (22) using the MuSiC method (23), which estimated the cell type composition of the bulk RNASeq samples across multiple bootstraps identifying ~80% as DANs and ~15% MG. Expression levels of relevant genes (*MERTK, GPR34, PROS1, C1QA, GAS6, TMEM119, TREM2*, and *P2RY12*), which were previously reported in MG (21, 24), were confirmed by qPCR in the microglial monocultures and MG^HC^-DAN^TRIP^ co-cultures, and were not detectable in DAN monocultures (fig. S1D). *P2RY12* was significantly up regulated further in MG^HC^-DAN^TRIP^ compared to MG alone (*P* = 0.0108) (fig. S1D). Thus, allogenic MG can be co-cultured with human DANs derived from patients with PD at a ratio that is roughly proportional to their brain composition (25) and could be used to study the MG response to PD pathology. We then analysed transcriptomic markers of metabolic, oxidative, and mitochondrial stress in co-cultures versus monocultures. We found that MG^HC^-DAN^TRIP^ co-cultures exhibited lower expression of stress-related genes compared to DAN^TRIP^ monocultures, suggesting that addition of MG is beneficial (Fig. 1D, fig. S2A, table S2). Quantification of fragmented nuclei in MAP2^+^ cells showed that neuronal death was not significantly increased in the presence of MG (Fig. 1E).

We therefore asked whether this improvement could be due to removal of aggregates from neurons. To this end, we used instant structured illumination microscopy (iSIM), which offers higher resolution for smaller aggregates in co-cultures of MG with either DAN^TRIP^ or their isogenic control DANs (DAN^ISO^). We identified puncta of serine 129 phosphorylated α-synuclein (pSyn), a widely used marker of endogenous α-synuclein aggregation (20, 26), in MG^HC^ only when co-cultured with DAN^TRIP^, not DAN^ISO^ (*P* < 0.0001) (Fig. 1F). This observation was associated with restoration of total intracellular α-synuclein levels in the MG-DAN^TRIP^ co-culture to isogenic control levels, as assessed by ELISA (Fig. 1G). Neurofilament light chain (NfL) levels in these cell lysates, measured by MSD electrochemiluminescence, were significantly increased when either DAN^TRIP^ or DAN^ISO^ were co-cultured with MG, with highest NfL levels detected in MG-DAN^ISO^ (*P* = 0.0004) followed by MG-SNCA^TRIP^ (*P* = 0.0146) co-cultures (Fig. 1H). Synapsin^+^ pre-synaptic terminals were significantly reduced in DAN^TRIP^ (*P* = 0.0307) but not DAN^ISO^ when co-cultured with MG (Fig. 1I and fig. S2B), accompanied by the detection of synapsin^+^ and MAP2^+^ puncta inside IBA1^+^ cells using 3D reconstruction (Fig 1J). Thus, MG sense and remove small aggregates that develop de novo inside neurons both at the axonal terminal (synapsin^+^) and somatodendritic (MAP2^+^) compartments, without causing neuronal injury.

### Microglia promote the clearance of large intraneuronal α-synuclein aggregates

**Fig. 2:**
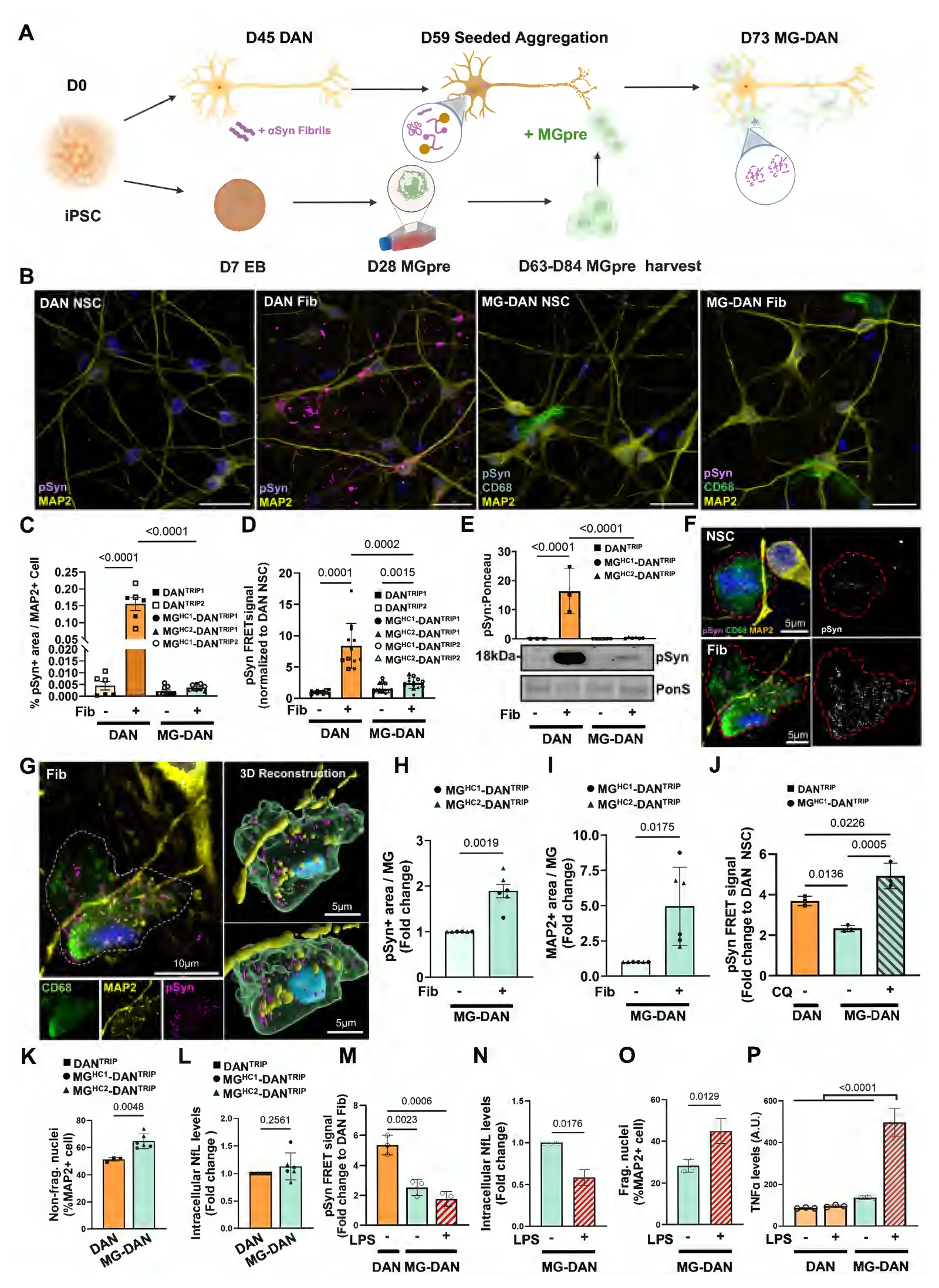
Microglia promote the clearance of fibril-seeded intraneuronal α-synuclein aggregates **(A)** Schematic diagram of the co-culture model. iPSC-derived DANs were seeded with fibrils at D45 and cultured for 2 weeks to allow the formation of abundant pSyn+ aggregates. Macrophage precursors (MGpre), differentiated from iPSC-derived embryoid bodies, were added to the fibril-seeded DANs at D59, and matured into microglia during the subsequent 2 weeks of co-culture, until D73. **(B)** Representative immunostaining of pSyn (magenta), CD68 (green), and MAP2 (yellow) in DAN and MG-DAN co-culture under non-seeded (NSC) or fibril-seeded (Fib) conditions. **(C)** Quantification of the total pSyn^+^ area per total scan area (% pSyn^+^ area), normalised to MAP2^+^ neuron count (n = 3 independent differentiations for DAN^TRIP1^, DAN^TRIP2^, MG^HC1^-DAN^TRIP1^, MG^HC2^-DAN^TRIP1^ and MG^HC1^-DAN^TRIP2^). **(D)** Quantification of pSyn signal by time-resolved FRET for each condition as indicated. Fibril-seeded DANs were co-cultured with MG (n=9 for DAN^TRIP1^, n=3 for DAN^TRIP2^, n=6 for MG^HC1^-DAN^TRIP1^, n=3 for MG^HC1^-DAN^TRIP2^, n=3 for MG^HC2^-DAN^TRIP1^, n=3 for MG^HC2^-DAN^TRIP2^). **(E)** Representative immunoblot of RIPA-insoluble fraction showing pSyn levels in DAN monocultures compared to MG^HC^-DAN^TRIP^ co-cultures under fibril-seeded conditions (n = 3 independent differentiations for DAN^TRIP1^, MG^HC1^-DAN^TRIP1^ and MG^HC2^-DAN^TRIP1^). **(F)** Representative Airyscan super-resolution images, showing pSyn^+^ puncta within CD68^+^ MG co-cultured with DANs under fibril-seeded conditions. **(G)** 3D reconstruction of Airyscan super-resolution image of MG co-cultured with DANs under fibril-seeded conditions in **(F)**, showing the presence of pSyn^+^ puncta and MAP2^+^ puncta within CD68^+^ MG. **(H)** Quantification of pSyn^+^ area within CD68^+^ MG (n = 3 independent differentiations for MG^HC1^-DAN^TRIP1^ and MG^HC2^-DAN^TRIP1^). **(I)** Quantification of MAP2^+^ area within CD68^+^ MG (n = 3 independent differentiations for MG^HC1^-DAN^TRIP1^ and MG^HC2^-DAN^TRIP1^). **(J)** Quantification of pSyn signal by time-resolved FRET at baseline and following chloroquine (CQ) treatment (10 µM) of MG^HC^-DAN^TRIP^ co-cultures (n = 3 independent differentiations for DAN^TRIP1^ and MG^HC1^-DAN^TRIP1^). **(K)** Quantification of the percentage of MAP2^+^ cells with non-fragmented nuclei in DAN^TRIP^ monocultures versus MG^HC^-DAN^TRIP^ co-cultures under seeded conditions (n=3 independent differentiations for DAN^TRIP1^, MG^HC1^-DAN^TRIP1^ and MG^HC2^-DAN^TRIP1^). **(L)** Quantification of NfL by MSD electrochemiluminescence in cell lysates from DAN^TRIP^ monocultures and MG^HC^-DAN^TRIP^ co-cultures under fibril-seeded conditions (n = 3 independent differentiations for DAN^TRIP1^, MG^HC1^-DAN^TRIP1^ and MG^HC2^-DAN^TRIP1^). **(M)** Quantification of pSyn by time-resolved FRET in LPS-treated co-cultures (n = 3 independent differentiations for DAN^TRIP2^ and MG^ISO^-DAN^TRIP2^). **(N)** Quantification of NfL by MSD electrochemiluminescence in cell lysates from DAN^TRIP^ monocultures and MG^HC^-DAN^TRIP^ co-cultures at baseline and following LPS treatment (n = 3 independent differentiations for MG^ISO^-DAN^TRIP2^). **(O)** Percentage of fragmented neuronal nuclei in MG^HC^-DAN^TRIP^ co-cultures at baseline and after LPS treatment (n = 3 independent differentiations for MG^ISO^-DAN^TRIP2^). **(P)** TNFα levels in DAN^TRIP^ monocultures and MG^HC^-DAN^TRIP^ co-cultures at baseline and after LPS treatment (n = 3 independent differentiations for DAN^TRIP2^ and MG^ISO^-DAN^TRIP2^). All graphs show mean ± SD and were analysed by one sample t-test (**H, I, L, O**), two-tailed t-test (**K, P**) or one-way ANOVA (**C, D, E, J, M, N**) followed by Tukey’s post hoc test. *Scale bar*, 20 μm unless otherwise specified.

We then assessed whether human MG clear larger, established intraneuronal aggregates. To this end, we co-cultured MG derived from two unrelated healthy iPSC lines with DAN^TRIP^ seeded with fibrils (Fig. 2A). Fibrils generated from recombinant α-synuclein (fig. S3A to S3E) were first added at D45, a timepoint at which we previously found iPSC-derived DANs to be electrophysiologically active (27). Two weeks after fibril addition (D59), intraneuronal α-synuclein aggregation was confirmed by immunostaining (fig. S3F) and time-resolved FRET (fig. S3G) for total pSyn in cell lysates. MG were then added to the neurons with established α-synuclein aggregates at D59 and co-cultured for two further weeks up to D73 (Fig. 2A).

Using immunofluorescence confocal microscopy, we found a significant reduction in intraneuronal pSyn^+^ aggregates in the presence of MG (Fig. 2B), as quantified by 3 different parameters: percentage area containing aggregates per MAP2^+^ neuron (Fig. 2C), number of aggregates per MAP2^+^ neuron (fig. S4A), and average aggregate size (fig. S4B) (*P* < 0.0001). This finding in seeded DAN^TRIP^ was further corroborated by time-resolved FRET for pSyn (*P* = 0.0002) (Fig. 2D) and immunoblotting of RIPA-insoluble pSyn in cell lysates (*P* < 0.0001) (Fig. 2E). Neuronal clearance of seeded aggregates was accompanied by a more prominent increase in pSyn^+^ puncta within MG as visualised by 3D rendering (Fig. 2F and 2G) and confirmed by quantification of the area of aggregates within MG (*P* = 0.0019) (Fig. 2H) or the number of MG containing aggregates (*P* = 0.0024) (fig. S4C). Additionally, the presence of MAP2^+^ puncta within MG was confirmed by quantifying the MAP2^+^ area per microglial cell (*P* = 0.0175) (Fig. 2I) and the number of MAP2^+^ puncta per microglial cell (*P* = 0.0005) (fig. S4D). We confirmed that MG in monocultures internalise aggregates when treated with fibrils (1μM) for 24h and this process was blocked by pre-treatment with a non-toxic concentration (10 µM) of cytochalasin D, an inhibitor of actin polymerisation used to block phagocytosis (*P* = 0.0015) (fig. S4E-G). Moreover, continuous treatment of the fibril-seeded co-cultures with a low (non-toxic) dose of the lysosomotropic agent chloroquine (10 µM), blocked aggregate clearance (*P* = 0.0005) (Fig. 2J). Collectively, these data indicate that healthy human MG clear fibril-induced intraneuronal α-synuclein aggregates, by a lysosomal/phagocytic mechanism. We also assessed in our co-culture model of seeded aggregation, iPSC-derived MG with the PD-associated *SNCA*^*TRIP*^, *LRRK2*^*G2019S*^ gene mutation (28) or the PD risk allele *GBA*^*N409S*^ (aka *GBA*^*N370S*^) that is implicated in lysosomal function (29). Under our experimental conditions, these mutations did not influence MG clearance of aggregates when compared to healthy controls as quantified by time-resolved FRET for pSyn levels (fig. S4H). In *SNCA*^*TRIP*^ MG monocultures we assessed lysosomal function relative to isogenic control MG at baseline and after addition of fibrils using Magic Red, which measures cathepsin B activity. These experiments did not reveal a lysosomal defect in *SNCA*^*TRIP*^ MG (fig. S4I). In control experiments, we tested and confirmed that endotoxin levels at the time of MG addition were low (<0.5 EU/ml) and comparable between the seeded and non-seeded conditions (fig. S4J). Therefore, endotoxin contamination is unlikely to be a confounding factor in our experiments.

We further verified that aggregate clearance is not due to MG-induced neuronal death. We found that under seeded conditions, the % neurons with healthy, non-fragmented nuclei were significantly higher in MG^HC^-DAN^TRIP^ co-culture compared to DAN^TRIP^ monoculture (*P* = 0.0048) (Fig. 2K) and NfL levels in cell lysates were similar in fibril seeded DAN^TRIP^ monocultures and MG^HC^-DAN^TRIP^ co-cultures (Fig. 2L). In contrast, MG treated with lipopolysaccharide (LPS, 100 ng/ml), a potent pro-inflammatory agent, also significantly reduced intraneuronal pSyn (*P* = 0.0006) (Fig. 2M). This was accompanied by neuronal damage, evidenced by significantly reduced NfL levels in cell lysates (*P* < 0.0176; Fig. 2N), increased neuronal death, indicated by a higher number of fragmented nuclei in MAP2^+^ cells (*P* = 0.0129) (Fig. 2O) and secretion of the cytotoxic cytokine TNFα (*P* < 0.0001) (30), which was minimally detectable at baseline (Fig. 2P, fig. S4K), even in MG^TRIP^ - DAN^TRIP^ co-cultures (fig. S4L). Thus, iPSC-based MG^HC^-DAN^TRIP^ co-cultures model both beneficial and harmful responses depending on the extent of MG activation but at basal conditions, the clearance of pSyn is not due to the death of aggregate-laden neurons.

### Microglial activation in response to intraneuronal α-synuclein aggregation is TREM2-independent and involves contact partly mediated by CD22 and P2RY12.

**Fig 3:**
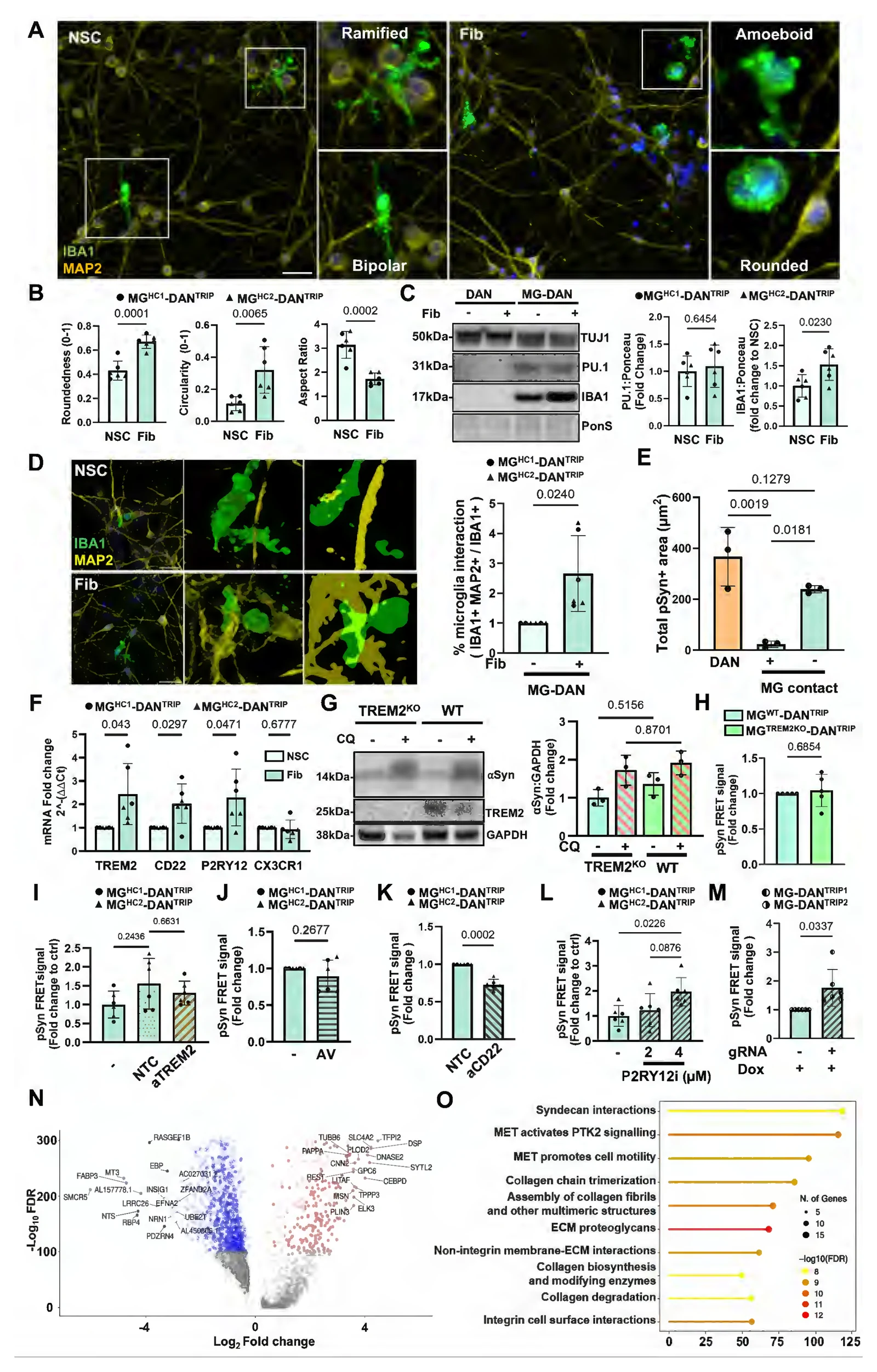
Microglial activation in response to intraneuronal α-synuclein aggregation is TREM2-independent and involves contact mediated by P2RY12 and CD22 **(A)** Confocal images of IBA1^+^ MG in MG^HC^-DAN^TRIP^ co-cultures exhibiting diverse morphologies, including ramified, bipolar, amoeboid, and rounded forms. **(B)** Quantification of IBA1-masked MG for roundedness, circularity and aspect ratio under non-seeded (NSC) and fibril-seeded (Fib) conditions (n=3 independent differentiations for MG^HC1^-DAN^TRIP1^ and MG^HC2^-DAN^TRIP1^) **(C)** Representative immunoblot and quantification of IBA1 and PU.1 in cell lysates, comparing MG^HC^-DAN^TRIP^ co-cultures under fibril-seeded to NSC conditions (n =3 independent differentiations for MG^HC1^-DAN^TRIP1^ and MG^HC2^-DAN^TRIP1^). **(D)** Representative confocal images of MG^HC^-DAN^TRIP^ co-cultures and corresponding 3D reconstructions with binary masks of the IBA1 and MAP2 channels, showing their overlap for quantitative analysis. Quantification using Mander’s coefficient of the percentage overlap of IBA1 with MAP2 in each image stack (n = 3 independent differentiations for MG^HC1^-DAN^TRIP1^ and MG^HC2^-DAN^TRIP1^). **(E)** Total pSyn^+^ area, with or without separation of the MG with transwells (n = 3 independent differentiations for MG^HC1^-DAN^TRIP1^). **(F)** qPCR analysis of relevant receptors (*TREM2, CD22, P2RY12, CX3CR1*) in fibril-seeded co-cultures, normalised to *GAPDH* and plotted relative to NSC conditions (n=3 independent differentiations for MG^HC1^-DAN^TRIP1^ and MG^HC2^-DAN^TRIP1^). **(G)** Immunoblotting of αSyn in TREM2 knockout (TREM2^KO^) and isogenic WT MG monocultures at baseline and after treatment with chloroquine (50 µM) (n=3 independent differentiations for MG^TREM2KO^ and MG^WT^). **(H)** Quantification of pSyn by time-resolved FRET in cell lysates from DAN^TRIP1^ co-cultured with either TREM2^KO^ or isogenic WT control MG (n=5 independent differentiations for MG^TREM2KO^-DAN^TRIP1^ and MG^WT^-DAN^TRIP1^). **(I)** Quantification of pSyn by time-resolved FRET in cell lysates from MG^HC^-DAN^TRIP^ co-cultures at baseline and after addition of TREM2 receptor blocking antibodies under fibril-seeded conditions (n = 3 independent differentiations for MG^HC1^-DAN^TRIP1^ and MG^HC2^-DAN^TRIP1^). **(J)** Quantification of pSyn time-resolved FRET in cell lysates from MG^HC^-DAN^TRIP^ co-cultures at baseline and after Annexin V (AV) treatment under fibril-seeded conditions (n = 3 independent differentiations MG^HC1^-DAN^TRIP1^ and MG^HC2^-DAN^TRIP1^). **(K)** Quantification of pSyn by time-resolved FRET in cell lysates from MG^HC^-DAN^TRIP^ co-cultures under fibril-seeded conditions when treated with anti-CD22 antibody (aCD22) compared to IgG non-targeting control (NTC) (n = 3 independent differentiations for MG^HC1^-DAN^TRIP1^ and MG^HC2^-DAN^TRIP1^). **(L)** Quantification of pSyn by time-resolved FRET, in cell lysates from MG^HC^-DAN^TRIP^ co-cultures treated with an increasing concentrations of P2RY12 inhibitor (P2RY12i) or control under fibril-seeded conditions (n = 3 independent differentiations MG^HC1^-DAN^TRIP1^ and MG^HC2^-DAN^TRIP1^). **(M)** Time-resolved FRET quantification of pSyn in cell lysates from MG^HC^–DAN^TRIP^ co-cultures following microglia-specific P2RY12 knockdown by CRISPRi under fibril-seeded conditions (n = 3 differentiations for MG^dCas9^-DAN^TRIP1^ and MG^dCas9^-DAN^TRIP2^). **(N)** Volcano plot of differentially expressed neuronal genes in MG^HC^–DAN^TRIP^ co-cultures versus DAN^TRIP^ monocultures; red indicates upregulation, blue indicates downregulation. **(O)** Gene ontology terms enriched in DANs when co-cultured with MG as determined by Single-cell RNA-seq analysis. Note enrichment of terms associated with extracellular matrix. All graphs show mean ± SD and were analysed by one sample t-test (**D, F, J, H, K, M**) two-tailed t-test (**B, C, H**), one-way ANOVA (**E, G, I, L**) followed by Tukey’s post hoc test. *Scale bar*, 20 μm unless otherwise specified.

We assessed the extent of MG activation in response to increasing burden of neuronal α-synuclein pathology by quantifying the morphology of IBA1^+^ microglia in co-culture with either DAN^TRIP^ or fibril-seeded DAN^TRIP^. In the fibril-treated conditions, microglia displayed significantly higher circularity (*P* < 0.0001), lower aspect ratio (*P* = 0.0065) and elevated roundness (*P* = 0.0002), indicating a transition to a more rounded, less ramified morphology, characteristic of reactive activation with diminished process arborisation (Fig 3A, B). Accordingly, immunoblotting of cell lysates showed that the microglial marker IBA1 was significantly upregulated in the fibril-seeded condition (*P* = 0.0230) whilst PU.1, a transcription factor involved in microglial development remained unchanged (Fig. 3C). Furthermore, qPCR analysis revealed increased expression of disease associated microglial (DAM) genes that were previously reported in Alzheimer’s disease (AD) and other neurodegenerative conditions (11). Specifically, we found that genes related to phagocytosis, lysosome function and lipid metabolism (e.g. *APOE, TLR4, LPL, CD68*) were upregulated in MG co-cultured with fibril-seeded DANs (*P* < 0.05) (fig. S5A).

To decipher how the two cell-types interact under pathological conditions, we first asked whether contact between MG and DANs changes in response to increased aggregation induced by fibrils. We quantified the spatial overlap between IBA1-positive microglia and MAP2^+^ neurons by performing 3D volumetric analysis and calculated the proportion of IBA1^+^ volume overlapping MAP2^+^ volume. We found that microglial contact with neurons was enhanced when intraneuronal aggregate burden was significantly increased with seeding (*P* = 0.0240) (Fig. 3D). Using a transwell co-culture system we confirmed that eliminating the contact between MG and DANs prevented the clearance of pSyn^+^ aggregates as quantified by immunofluorescence (Fig. 3E and fig. S5B).

Contact was accompanied by significantly increased expression of the DAM-related innate immune receptors TREM2 (*P* = 0.043), CD22 (*P* = 0.0297), as well as the homeostatic receptor P2RY12 (*P* = 0.0471) but not CX3CR1 or TMEM119 (Fig. 3F, fig. S5A), suggesting that microglia engage a hybrid activation state tailored to intraneuronal α-synuclein aggregates. To investigate how microglia recognise aggregate-laden neurons, we first tested TREM2 because it is required for the transition of MG to a DAM phenotype (6) in neurodegenerative disease models and it is a reported receptor for phosphatidylserine which is exposed on damaged neurons (31). qPCR revealed significant up-regulation of *TYROBP* (*P* = 0.0177) which interacts with TREM2, as well as *AKT (P* = 0.0373) and *PI3K* (*P* = 0.0386*)*, which are downstream mediators in the TREM2 signalling pathway (fig. S5C). Whether TREM2 is a genetic modifier in PD is controversial (32, 33) and, its function in human models of α-synuclein pathology is unclear. To address this issue, we used iPSC-derived MG with CRISPR/Cas9 mediated knockout of TREM2 and their isogenic wildtype control (fig. S5D). Firstly, we added fibrils to either TREM2^KO^ or isogenic wildtype MG monocultures and measured α-synuclein degradation 24 h later at baseline or in the presence of chloroquine (50 μM). We found that fibrils were degraded to a similar extent by TREM2^KO^ or wildtype MG, as assessed by immunoblotting for total α-synuclein, and degradation was blocked by chloroquine (Fig. 3G). TREM2^KO^ or wildtype MG were also co-cultured with fibril-seeded DAN^TRIP^ as described earlier and pSyn was measured by time-resolved FRET. These experiments did not reveal any difference in the clearance of pSyn aggregates between TREM2^KO^ versus wildtype isogenic control MG (Fig. 3H). To ensure these results were not due to compensation from chronic lack of TREM2 in this model, we also assessed the effect of acute TREM2 inhibition using specific TREM2-blocking antibodies (kindly provided by Eli Lilly). We found that TREM2 blocking antibodies at 10 µg/ml did not modify the clearance of pSyn aggregates when compared to non-targeted control IgG (Fig. 3I). Furthermore, recombinant Annexin V (0.2 µg/ml, added 24 h before and during the two weeks of co-culture), which binds to and blocks phosphatidylserine signalling, had no effect (Fig. 3J). In control experiments, we stained non-permeabilised neurons with anti-Annexin V antibody (fig. S5E). Quantification of % of Annexin V^+^ area (*P* = 0.0133) (fig. S5F) or fluorescence intensity (*P* = 0.0119*)* (fig. S5G) confirmed Annexin V binding, which was increased in fibril-seeded neurons treated with Annexin V. Thus, phosphatidylserine which is exposed on the surface of neurons with aggregates, and TREM2 on MG are dispensable for microglial aggregate clearance in this human model.

We then asked whether contact may involve both homeostatic and DAM receptors acting in concert. We assessed CD22, an α2,6-linked sialic acid receptor previously shown to prevent aggregate clearance (10) and P2RY12, a G-protein-coupled receptor activated by neuronal damage (34, 35) that were up-regulated with increased aggregation. We found that continuous treatment during the 2-week co-culture period with blocking antibodies against CD22 (5 µg/ml) significantly enhanced aggregate clearance (*P* = 0.0002) (Fig. 3K), whereas treatment with the P2RY12 inhibitor PSB-0739 (4 μM) significantly increased residual aggregate load by 2-fold (*P* = 0.0226) (Fig. 3L), as assessed by time-resolved FRET for pSyn. To further investigate candidate effectors that are not readily targetable with antibodies, we integrated a doxycycline-inducible CRISPR-dCas9 interference cassette into one of the healthy control iPSC lines (fig. S6A to S6G). We knocked down *P2RY12* expression specifically in MG by lentiviral transduction at the iPSC stage with pooled gRNAs targeting the promoter region of *P2RY12* (fig. S6H), followed by differentiation into MG and subsequent addition to DANs. A 65% reduction in *P2RY12* mRNA as assessed using qPCR in the co-culture at D73 (*P* = 0.0004) (fig. S6I) significantly increased the residual neuronal pSyn^+^ aggregates by 1.8-fold as measured by time-resolved FRET (P = 0.0337) (Fig. 3M). Collectively, these data suggest that neuron-microglial contact is required for the removal of intraneuronal aggregates, and this process is fine-tuned by multiple signals such as P2RY12, which senses neuronal damage and CD22, which limits phagocytic activity.

Finally, we performed single-cell RNA sequencing (scRNA-seq) on DAN^TRIP^ seeded with fibrils alone and compared them to seeded DAN^TRIP^ co-cultured with MG. Unbiased clustering across the combined conditions revealed eight distinct clusters (fig. S7A). Clusters 0, 6, and 7 were identified as neurons in monocultures, based on their expression of dopaminergic markers (fig. S7B, S7C) and additional neuronal markers (fig. S7D) (n=22,045), whereas clusters 1 and 2 from MG^HC^-DAN^TRIP^ co-cultures were identified as neurons (n=6,984). Cluster 3 was classified as a microglial population, as evident by the relevant marker expression profile (fig. S7E) (n=2,676). We found that neurons cocultured with microglia exhibited a distinct transcriptional response compared to DAN^TRIP^ monoculture, principally in genes implicated in extracellular matrix (ECM) remodelling (Fig. 3N, table S3). Gene ontology analysis revealed enrichment in pathways such as syndecan interactions, collagen organisation, and MET signalling, non-integrin-membrane ECM interactions, suggesting that neurons actively modify their ECM microenvironment when in contact with MG (Fig. 3O). These findings suggest that active organisation of the neuronal ECM may further refine interactions with MG. Finally, we analysed transcriptomic markers related to metabolic activity, hypoxia, and mitochondrial stress in co-cultures versus neuronal monocultures. These results corroborated our earlier bulk RNA-seq findings, i.e. that stress markers are reduced in neurons co-cultured with MG when compared to neuronal monocultures, with the most pronounced effect observed under fibril seeded conditions (fig. S7F).

### Single-cell RNA sequencing identifies clusters of α-synuclein-related DAM.

**Fig 4:**
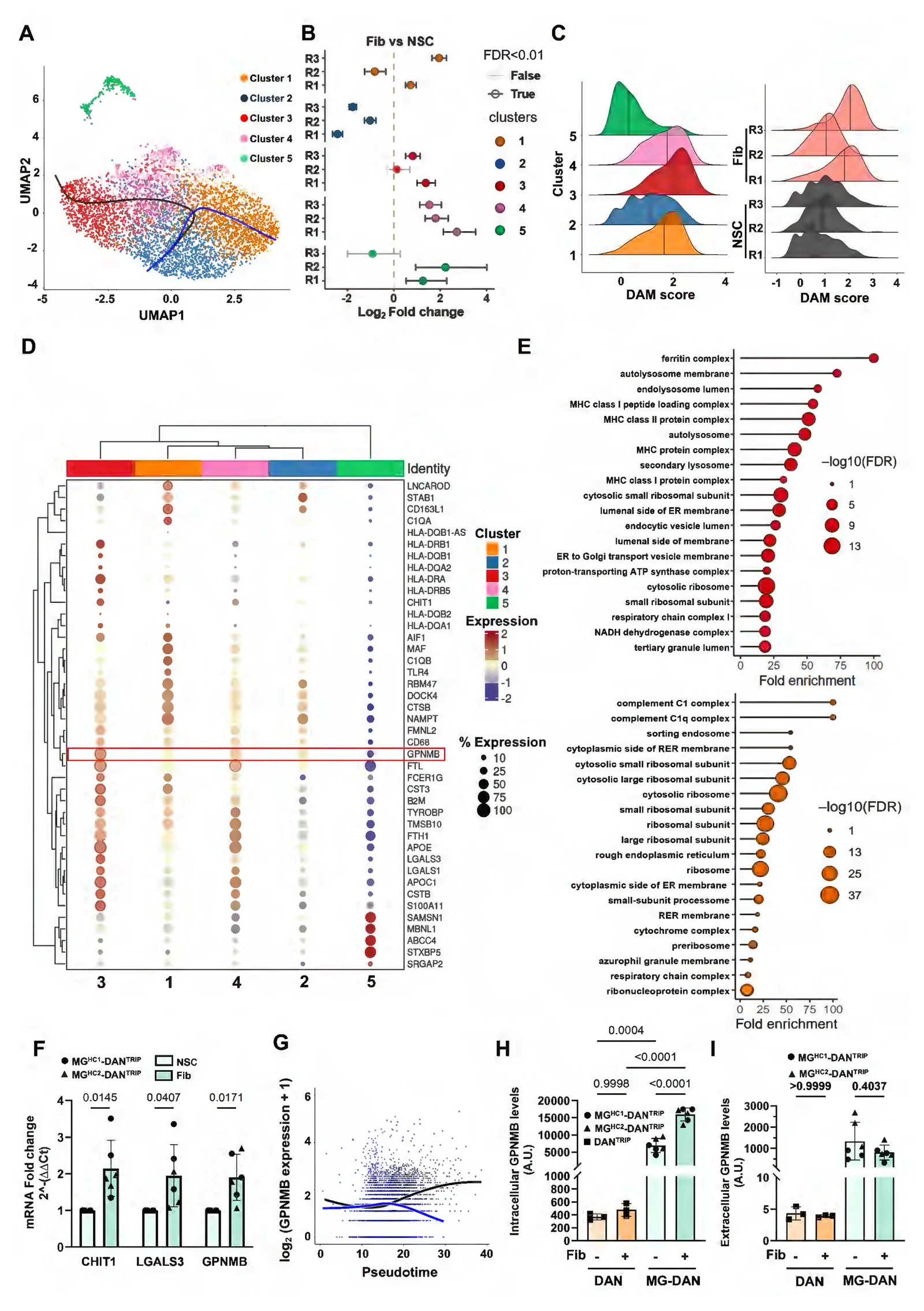
Single-cell RNA sequencing identifies clusters of α-synuclein-related DAM. **(A)** UMAP visualisation of MG single-cell transcriptomes revealed 5 distinct clusters and 2 pseudo-time trajectories. **(B)** Log_2_ fold change in MG cluster abundance between NSC- and fibril-seeded conditions. Open circles indicate FDR < 0.01. R1–R3 denote three independent differentiations for MG^HC1^-DAN^TRIP1^. **(C)** DAM signature module scores for microglial clusters 1 to 5 (left) and for microglia in NSC and fibril-seeded conditions (right). R1–R3 denote three independent differentiations for MG^HC1^-DAN^TRIP1^. Vertical lines indicate group medians. *p* <0.0001 **(D)** Dot plot summarising the average expression (blue-to-brown colour scale) and percentage of cells (dot size) expressing the top marker genes for each microglial cluster. Marker genes were identified by pairwise differential-expression testing with MAST, distinguishing each cluster from every other cluster. Note the high expression of *GPNMB* in clusters 3 and 4. **(E)** Gene ontology (GO) analysis of cellular component terms showing the top 20 categories ranked by fold enrichment score (adjusted p <0.05) across pseudotime along the two branches originating from cluster 2: the trajectory toward clusters 3 and 4 (upper panel) and the trajectory toward cluster 1 (lower panel). **(F)** *CHIT1, LGALS3*, and *GPNMB* expression in MG^HC^–DAN^TRIP^ quantified by qPCR under fibril-seeded conditions (n=3 independent differentiations for MG^HC1^-DAN^TRIP1^ and MG^HC2^-DAN^TRIP1^). **(G)** Expression of *GPNMB* along the pseudotime trajectories. Smoothed trend lines indicate the fitted expression trajectories across pseudotime (black line toward clusters 3 and 4; blue line toward cluster 1). Each dot represents one cell; y-axis shows log2-transformed GPNMB expression; x-axis shows pseudotime. **(H)** Quantification of GPNMB protein levels by ELISA in cell lysates from DAN^TRIP^ monocultures and MG^HC^–DAN^TRIP^ co-cultures at baseline and under fibril-seeded (Fib) conditions (n = 3 independent differentiations for DAN^TRIP1^, MG^HC1^-DAN^TRIP1^ and MG^HC2^-DAN^TRIP1^). **(I)** Quantification of GPNMB protein levels by ELISA in conditioned media from DAN monocultures and MG-DAN co-cultures at baseline and under fibril-seeded (Fib) conditions (n = 3 independent differentiations for MG^HC1^-DAN^TRIP1^ and MG^HC2^-DAN^TRIP1^). All graphs show mean ± SD and were analysed by Mann–Whitney U test (**C**), one sample t-test (**F**), two-tailed t-test or one-way ANOVA (**H, I**) followed by Tukey’s post hoc test. R1, R2, R3 refer to biological replicates from n=3 independent differentiations.

To fully define the molecular phenotype of human MG in our model and assess its relevance to human pathology, we performed scRNA-seq on MG isolated from co-cultures with DAN^TRIP^, with or without fibrils. In total, we profiled 5,640 MG transcriptomes across all conditions (Fig. 4A). Cells from all three replicates exhibited enrichment in microglial identity and maturation markers (fig. S8A), consistent with our bulk RNA-seq data and confirming successful differentiation.

Unbiased clustering across combined conditions revealed five distinct clusters, consistently detected across replicates. Clusters 3 and 4 were more abundant in the fibril-seeded condition (Fig. 4B, fig. S8B). Cluster 5 exhibited a proliferative state characterized by high expression of cell cycle-associated genes. Among the non-proliferative microglial clusters, clusters 3 and 4 displayed the strongest DAM signatures, whereas cluster 2 displayed the weakest (Fig. 4C). Comparison of these microglial clusters to previous analyses (13) further corroborated that clusters 3 and 4 most closely resemble neurodegeneration-associated DAM states, whereas cluster 2 aligns closer to a homeostatic phenotype and cluster 5 to a proliferative state (fig. S8C).

Pseudotime analysis indicated a trajectory from cluster 2, representing the homeostatic state, toward either cluster 3 and cluster 4, or cluster 1, reflecting dynamic transitions in microglial states in response to α-synuclein pathology (Fig. 4A). Differential gene expression analysis showed that cluster 3, which exhibited the strongest DAM signature, was enriched for canonical DAM genes including *TYROBP, APOE*, and the PD GWAS candidate *GPNMB* as well as antigen-presenting genes such as *B2M* and the PD GWAS-linked genes *HLA-DRB1* and *HLA-DQ* (Fig. 4D, fig. S8D; table S4). Cluster 4 was enriched for *TLR4* and complement components *C1QB* (Fig. 4D, fig. S8D; table S4).

To uncover molecular pathways underlying the divergent pseudotime trajectories, we performed GO cellular component analysis on branch-specific differentially expressed genes (Fig. 4E; table S5). The trajectory toward cluster 3 and 4 was enriched in ferritin complexes, autolysosomal and endolysosomal membranes, MHC complexes, and mitochondrial subunits. In contrast, the trajectory toward cluster 1 showed enrichment for complement components (especially C1), ribosomal and rough ER subunits, cytosolic small ribonucleoprotein particles, and secretory granules, suggesting a microglial state characterised by enhanced protein synthesis and secretion, particularly of complement proteins such as *C1Q*. We validated the up-regulation of DAM-associated genes *CHIT1 (P* = 0.0145), *LGALS3 (P* = 0.0407), *GPNMB (P* = 0.0171) by qPCR in n=3 additional differentiations per iPSC-MG line (Fig. 4F). Increased *GPNMB* expression was further confirmed when MG were co-cultured with DAN^TRIP^ relative to isogenic control neurons (*P* = 0.0247) (fig. S8E)

The PD GWAS candidate gene *GPNMB* was highly expressed in DAM microglial clusters 3 and 4, and its expression increased along this pseudotime trajectory (black line in Fig. 4G and 4A). We confirmed using ELISA (Fig. 4H) and immunoblotting (fig. S8F) that GPNMB protein is up-regulated in MG^HC^-DAN^TRIP^ co-culture lysates with minimal detection in DAN^TRIP^ monocultures in n=3 further independent differentiations per iPSC-MG line (*P* < 0.0001). In further control experiments, we tested and confirmed that *GPNMB* mRNA levels were minimally detected in DAN monocultures but were increased in co-cultures (*P* < 0.0001) (fig. S8G). We then investigated whether GPNMB is secreted, by measuring with ELISA GPNMB protein levels in conditioned media from DAN monocultures and MG-DAN co-cultures. These experiments showed that GPNMB was similarly secreted in MG-DAN co-cultures irrespective of the extent of neuronal aggregation but was barely detectable in DAN conditioned media at baseline or after seeding with fibrils (Fig. 4I).

### GPNMB interacts with α-synuclein in MG, promotes aggregate clearance and is up-regulated in post-mortem substantia nigra tissue from patients with PD

**Fig 5:**
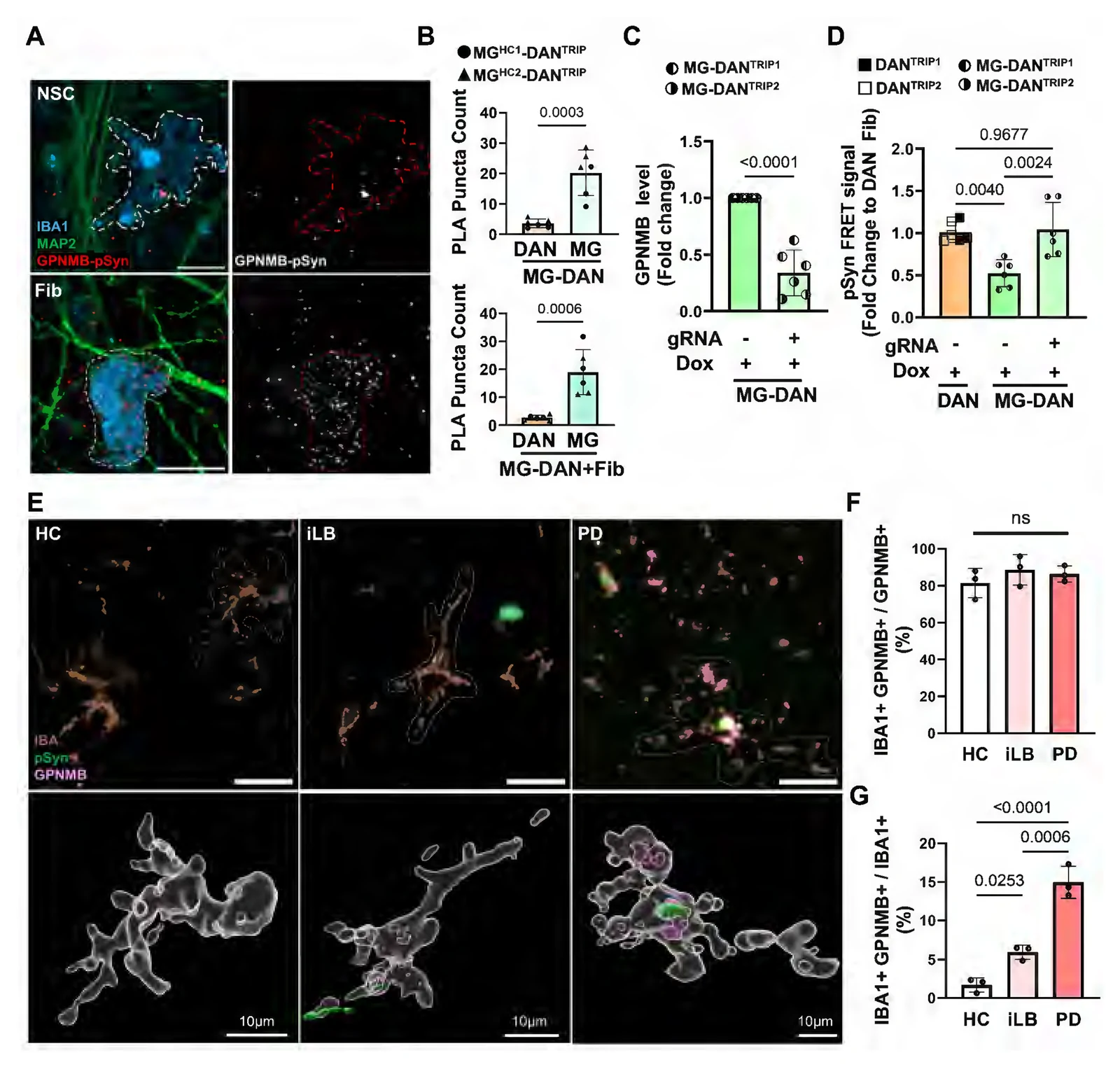
GPNMB interacts with α-synuclein and regulates aggregate clearance by MG. **(A)** Representative image of GPNMB and pSyn interaction (red) using proximity ligation assay with concomitant immunostaining for IBA1^+^ MG^HC^ (blue) and MAP2^+^ DAN^TRIP^ neurons (green) under non-seeded conditions (top) and Fib-seeded conditions (bottom). Right image in black and white shows the same field, highlighting the GPNMB–pSyn PLA puncta (n = 3 independent differentiations for MG^HC1^-DAN^TRIP1^ and MG^HC2^-DAN^TRIP1^) **(B)** Quantification of GPNMB and pSyn (red) interaction using proximity ligation assay in IBA1^+^ MG^HC^ and MAP2^+^ DAN^TRIP^ neurons under non-seeded (top) and fibril-seeded conditions (bottom) (n = 3 independent differentiations for MG^HC1^-DAN^TRIP1^ and MG^HC2^-DAN^TRIP1^). **(C)** Quantification of GPNMB protein levels by ELISA levels in cell lysates from MG^dCas9^-DAN^TRIP^ co-cultures (n = 3 independent differentiations for MG^dCas9^-DAN^TRIP1^ and MG^dCas9^-DAN^TRIP2^). **(D)** Time-resolved FRET quantification of pSyn in cell lysates from MG^dCas9^-DAN^TRIP^ co-cultures with or without GPNMB knockdown and in DAN^TRIP^ monocultures (n = 3 independent differentiations for DAN^TRIP1^, DAN^TRIP2^, MG^dCas9^-DAN^TRIP1^ and MG^dCas9^-DAN^TRIP2^). **(E)** Representative immunostaining for pSyn (green), IBA1 (brown), and GPNMB (magenta) in post mortem substantia nigra tissue from healthy donors (HC) and donors with incidental Lewy bodies (iLB) or PD. Lower panels show 3D reconstructions of identified IBA1^+^ cells containing GPNMB and pSyn. **(F)** Percentage of GPNMB^+^ voxels overlapping IBA1^+^ voxels in substantia nigra across HC, iLB, and PD (n=3 donors per group). **(G)** Percentage of IBA1^+^ voxels overlapping GPNMB^+^ voxels in substantia nigra across HC, iLB, and PD (n=3 donors per group). All graphs show mean ± SD and were analysed by two-tailed t-test (**B**), one sample t-test (**C**), one-way ANOVA (**D, F, G**) followed by Tukey’s post hoc test. *Scale bar*, 20 μm unless otherwise specified.

We next assessed whether GPNMB is involved in aggregate clearance. Using a proximity ligation assay, we found that GPNMB interacted with pSyn^+^ aggregates in MG rather than DANs under both non-seeded (*P* = 0.0003) and seeded (*P* = 0.0006) conditions (Fig. 5A, 5B). To investigate the function of GPNMB in microglial-mediated clearance of pSyn^+^aggregates, we used our doxycycline-inducible CRISPRi system to selectively knock down *GPNMB* expression in healthy control MG. Knockdown was achieved via lentiviral transduction of iPSCs with a pool of three gRNAs targeting distinct regions of the *GPNMB* promoter (fig. S9A-C), followed by differentiation into MG and subsequent co-culture with DAN^TRIP^. Continuous doxycycline treatment from Day 1 of differentiation resulted in a 66% reduction in GPNMB protein levels in the co-culture system upon gRNA expression (±gRNA) as measured by ELISA (*P* < 0.0001) (Fig. 5C). This degree of *GPNMB* knockdown in MG was sufficient to block the clearance of neuronal pSyn^+^ aggregates as measured by time-resolved FRET (Fig. 5D and fig. S9D). *GPNMB* knockdown, did not affect MG morphology or MG interactions with neurons in the co-cultures (fig. S9E-F). Collectively, these data suggest that GPNMB is a critical effector of α-synuclein aggregate clearance by MG.

Finally, we confirmed that GPNMB is predominantly expressed in IBA1-positive microglia in the substantia nigra of healthy control (81%), incidental Lewy body (iLB; 92%), and PD (87%) brains based on the % GPNMB^+^ voxels overlapping IBA1^+^ voxels (Fig. 5E, 5F, table S6). Furthermore, we found that within the IBA^+^ microglia, GPNMB levels were significantly increased in a graded manner with lowest voxels detected in HC, intermediate in iLB (*P* = 0.0253) and highest in PD (*P* < 0.0001) (Fig. 5G).

### Autocrine regulation of microglia by IL-10 limits α-synuclein aggregate clearance.

**Fig 6:**
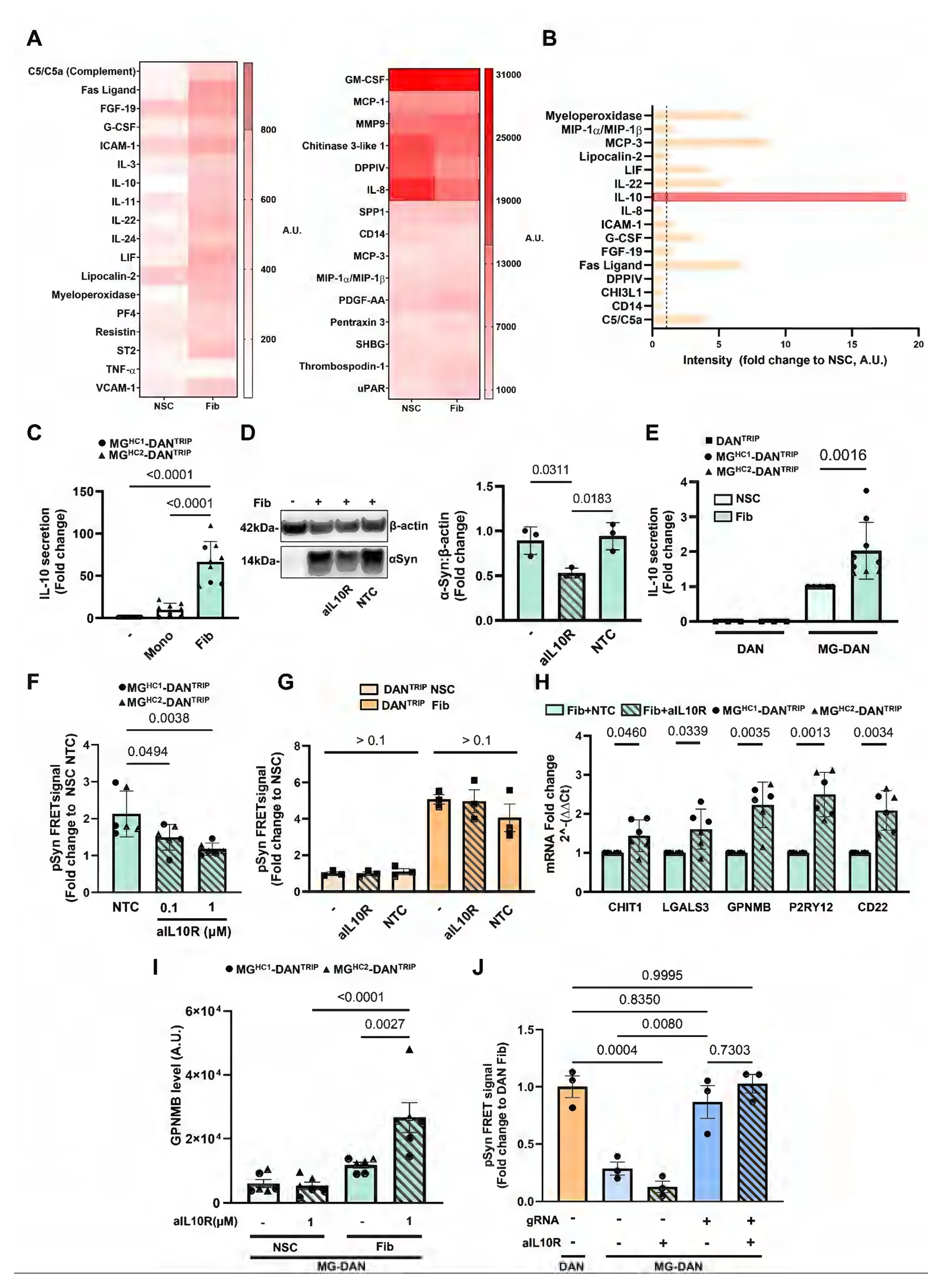
Autocrine regulation of microglia by IL-10 limits α-Synuclein aggregate clearance. **(A)** Secretome analysis of MG^HC^-DAN^TRIP^ co-cultures. Heatmaps showing cytokine signal abundance (AU) in conditioned media under non-seeded (NSC) and fibril-seeded (Fib) conditions. **(B)** Quantification of secreted cytokines showing the largest fold change in MG^HC^-DAN^TRIP^ co-culture conditioned media under Fib-seeded conditions relative to non-seeded controls (NSC). **(C)** IL-10 secretion in conditioned media of MG monocultures as quantified by ELISA at baseline and after treatment with fibrils or monomeric αSyn (Mono) (n = 3 independent differentiations for MG^HC1^-DAN^TRIP1^ and MG^HC2^-DAN^TRIP1^). **(D)** Representative immunoblot and quantification of αSyn levels in cell lysates from MG monocultures treated with fibrils and either anti-IL10Rα antibody (1μg/ml) or isotype NTC IgG (n=3 for MG^HC1^). **(E)** IL-10 levels as quantified by ELISA in cell lysates from DAN^TRIP^ monocultures and MG^HC^-DAN^TRIP^ co-cultures under non-seeded (NSC) or fibril-seeded (Fib) conditions. (n≥3 independent differentiations for DAN^TRIP1^, MG^HC1^-DAN^TRIP1^ and MG^HC2^-DAN^TRIP1^). **(F)** Time-resolved FRET quantification of pSyn in cell lysates from fibril-seeded MG^HC^-DAN^TRIP^ co-cultures treated with increasing concentrations of IL-10Rα blocking antibodies or NTC IgG (n=3 independent differentiations for MG^HC1^-DAN^TRIP1^ and MG^HC2^-DAN^TRIP1^). **(G)** Time-resolved FRET quantification of pSyn in cell lysates from DAN^TRIP^ monocultures under non-seeded (NSC) or fibril-seeded (Fib) conditions; cells were treated with 1 μg/ml anti-IL-10Rα or NTC IgG (n = 3 for DAN^TRIP1^). **(H)** qPCR analysis of DAM markers (*CHIT1, LGALS3, GPNMB, P2RY12, CD22*), normalised to *GAPDH*. Data show gene expression in anti-IL-10Rα or IgG (NTC) treated fibril-seeded MG^HC^-DAN^TRIP^ co-cultures (n= 3 independent differentiations for MG^HC1^-DAN^TRIP1^ and MG^HC2^-DAN^TRIP1^). **(I)** Quantification of GPNMB protein levels by ELISA in cell lysates from MG^HC^-DAN^TRIP^ co-cultures treated anti-IL-10Rα antibodies or NTC IgG, under non-seeded (NSC) or fibril-seeded (Fib) conditions (n = 3 independent differentiations for MG^HC1^-DAN^TRIP1^ and MG^HC2^-DAN^TRIP1^). **(J)** Time-resolved FRET quantification of pSyn in cell lysates from DAN monocultures and MG-DAN co-cultures. MG-DAN co-culture conditions included MG *GPNMB* knockdown or control ±IL-10 receptor blockade (aIL10R; n = 3 independent differentiations for DAN^TRIP1^ and MG^dCas9^-DAN^TRIP1^). All graphs show mean ± SD and were analysed by one sample t-test (**H**) or one-way ANOVA (**C, D, E, F, G, I, J**) followed by Tukey’s post hoc test.

To identify potential secreted regulators of the microglial phenotype, we used a membrane-based proteome array of 105 cytokines and chemokines and analysed their relative detected intensity (AU) in pooled co-culture conditioned media from three independent differentiations at D73. This analysis identified 48 differentially secreted cytokines in the conditioned media of fibril-seeded co-cultures compared to non-seeded co-cultures (Fig. 6A). We observed high levels of IL-10 under conditions of seeded aggregation relative to non-seeded baseline (Fig. 6B). Tumour Necrosis Factor-α (TNFα), a key cytokine of microglial-mediated cytotoxicity, that is down-regulated by IL-10 signalling (36), was not detectable under these conditions, in contrast to what was seen earlier in LPS-treated co-cultures (Fig. 2P).

We confirmed by ELISA that addition of fibrillar but not monomeric α-synuclein significantly increased IL-10 secretion in iPSC-derived MG monocultures (*P* < 0.0001) (Fig. 6C). Blocking the action of IL-10 in MG monocultures with anti-IL10 receptor alpha (anti-IL-10Rα) antibodies (1 µg/ml) significantly increased the degradation of α-synuclein fibrils when compared to non-targeting control (NTC) IgG (P = 0.0183) (Fig. 6D). Thus, IL-10 may act as an autocrine negative regulator of microglial degradation of α-synuclein fibrils. In the MG^HC^-DAN^TRIP^ model, we first showed that IL-10 was detectable only in the conditioned media from co-cultures, not DAN^TRIP^ monocultures, and significantly increased further when MG were co-cultured with fibril-seeded DAN^TRIP^ that exhibit more extensive α-synuclein aggregation (*P* = 0.0016) (Fig. 6E). We then assessed the effect of anti-IL-10Rα blocking antibodies (1 µg/ml) for the 2-week duration of the co-culture of MG with DAN^TRIP^ that were previously seeded with fibrils. This analysis revealed a dose-dependent reduction in pSyn levels, indicating that blocking IL-10 signalling enhances microglial-induced clearance of pSyn^+^ aggregates when compared to NTC IgG (*P* = 0.0038) (Fig. 6F). In control experiments, we tested and confirmed that IL-10Rα blockade did not have a direct effect on pSyn^+^ aggregates in neuronal monocultures (Fig. 6G). We also assessed factors that were strongly released across co-culture conditions such as MCP-1 (using inhibitor RS504393, 2 µM), SPP1 (using anti-SPP1 antibody, 5 µg/ml), and MMP9 (using inhibitor MMP9i 444278, 5 µM). These treatments did not modify microglial clearance of neuronal pSyn aggregates (fig. S10A and S10B).

To assess whether IL-10 signalling influences microglial activation, we performed - qPCR in co-cultures treated with either anti-IL10Rα or NTC IgG antibodies under fibril-induced aggregation conditions. This analysis showed that blocking IL-10Rα significantly increased the expression of target genes identified in scRNAseq including *GPNMB* (*P* = 0.0035) as well as *CD22* (*P* = 0.0034) and *P2RY12* (*P* = 0.0013) (Fig. 6H and fig. S10C).

It is noteworthy, that LPS treatment, which is associated with a cytotoxic state, does not up-regulate *GPNMB* expression in either microglial monoculture (fig. S10D) or in fibril-treated co-cultures (fig. S10E). The IL-10 induced upregulation of GPNMB was further confirmed by ELISA (P = 0.0027) (Fig. 6I). To functionally delineate this signalling cascade, we added anti-IL10Rα antibodies to co-cultures where *GPNMB* was specifically knocked down in MG using CRISPRi. We found that knockdown of GPNMB muted the effect of IL-10 blockade on aggregate clearance (Fig. 6J, fig. S10F). These findings suggest that IL-10 acts as an autocrine negative regulator of GPNMB-mediated microglial clearance of neuronal α-synuclein aggregates.

## Discussion

Most research on microglia in PD has involved the study of animal models or short-term rodent co-culture systems, whereas the chronic response of human microglia to aggregate-laden human DANs is unknown. We developed iPSC-derived microglia and DAN co-cultures to determine microglial mechanisms of aggregate clearance in the human condition. Our findings identify distinct microglial states that exert beneficial effects, challenging the dominant view from animal studies, that in PD microglial activation is principally detrimental (9, 37).

A key finding of our study is that microglia trogocytose α-synuclein aggregates from the neuronal sub-compartment where they reside, based on the detection of pSyn^+^, synapsin^+^ (pre-synaptic) and MAP2^+^ (somatodendritic) puncta within microglia and the requirement for direct contact between the two cell types. Initial α-synuclein aggregates in animal models (38) and human PD brain (39) localise predominantly in presynaptic terminals, raising the possibility that these are the earliest sites of microglial trogocytosis. Presynaptic microglial trogocytosis was previously shown to remodel synapses (40). Our study uncovers some of the underpinning signals and suggests that MG trogocytose intraneuronal aggregates throughout the neuronal compartments. This allows microglia to preserve neuronal integrity while simultaneously clearing toxic species. Our data suggest that this action is mediated by lysosomal degradation but is not detectably compromised by PD-associated *LRRK2*^*G2019S*^ or *GBA*^*N370S*^ mutations in MG. Similarly, *SNCA*^*TRIP*^ in MG did not affect pSyn clearance, cathepsin B activity or TNFα secretion under the conditions tested. However, further studies across additional iPSC clones and lines will be important to fully exclude a potential modifier effect. LPS activated MG secreted TNFα and triggered neuronal death, demonstrating that prolonged or over-activation is detrimental in this model, as previously suggested in other model systems (30, 41).

Multiple mechanisms of α-synuclein uptake by microglia have been described, primarily in rodent models such as endocytosis of secreted α-synuclein (5, 42) or tunnelling nanotube–mediated transfer (43). It is possible that such mechanisms also contribute in our human model, especially in the early stages as direct contact between microglia and neurons was required for the removal of intraneuronal aggregates. However, the mechanism we identified also differs substantially in that it is unaffected by TREM2 knockout or the LRRK2^G2019S^ mutation, two genes that regulate tunnelling nanotube mediated transfer from neurons to MG (18). Additionally, blockade of neuronal phosphatidylserine, a well-established generic “eat-me” signal via microglial TREM2 (31), had no impact on aggregate removal in agreement with the dispensability of TREM2 in our system. Whether these differences are due to species- or context-specific mechanisms of microglial function remains to be seen. Phosphatidylserine blockage prevented microglial phagocytosis of GABA neurons but not DANs, suggesting that microglia utilise different receptor repertoire for interactions with different types of neurons (44).

Instead, we found that trogocytosis is more nuanced and mediated by multiple effectors acting in concert, including sensing signals via P2RY12 and negative regulation via the sialic-acid–binding immunoglobulin-like lectin CD22 and the anti-inflammatory cytokine IL-10. *P2RY12* gene variants have been implicated in PD pathogenesis (45) and CD22 was previously identified in animal models as an age-related negative regulator of microglial phagocytosis including removal of α-synuclein aggregates (12). IL-10 levels are increased in the blood of patients with PD (46) and IL-10 over-expression in animal models increased α-synuclein aggregate burden and reduced survival (47). It is likely that additional mechanisms contribute such as remodelling of the ECM by neurons and signals from neighbouring glia, including microglia subtypes with secretory function as suggested by our transcriptomic profiling. This withstanding, our study demonstrates that signals such as CD22 and IL-10 may act as brakes on microglial surveillance or activation to ensure it remains proportionate to the extent of neuronal pathology. Therefore, their graded inhibition could restore or amplify degradative capacity, especially in the presence of proteopathic stress. Defining this regulatory “sweet spot” of microglial activation is critical for designing therapeutics to enhance aggregate clearance while minimising inflammatory damage.

We found that aggregate clearance coincided with a shift in microglial morphology and gene expression profile. The microglial morphological transformation to a less ramified, more amoeboid state is consistent with the long held observation in post-mortem PD brains (48). This was accompanied in our model by up-regulation of DAM markers, including lysosomal and phagocytic genes and innate immune receptors, which have been implicated in neurodegeneration (49). Although DAM signalling may be broadly conserved across neurodegenerative diseases, our results suggest that the DAM response is also adapted to α-synuclein pathology. For example, unlike its critical microglial function in AD (11, 50), TREM2 was not required for α-synuclein aggregate clearance in our model and it is not an established risk gene in PD (32, 33). Microglia in our model retained clearance competence without inducing neuronal death, despite up-regulation of DAM-associated genes, as evident by the up-regulation and retained function of the homeostatic gene *P2RY12* and the lack of TNFα secretion. We identified two transcriptionally distinct trajectories that diverge along separate paths in pseudotime analysis. The first trajectory was marked by upregulation of *C1Q*, a key initiator of the classical complement cascade traditionally involved in developmental synaptic pruning (51) and genes implicated in endosomal sorting, protein synthesis and secretion. The second trajectory, encompassing clusters 3 and 4, was expanded under conditions of increased aggregate abundance and displayed the DAM-like profile. This includes upregulation of *APOE, APOC1, LGALS3, CHIT1, FTH1*, and *FTL*, genes linked to lipid metabolism, phago-lysosomal degradation, and iron sequestration, which are dysregulated in PD and other neurodegenerative conditions. The enrichment of *HLA-DRB1*, a well-established GWAS locus for PD risk (52), alongside other MHC class II genes in cluster 3, which exhibits the strongest DAM signature, suggest a role for MHC-II–mediated microglial immunosurveillance in response to α-synuclein pathology. *HLA-DR* is highly expressed in reactive microglia in the substantia nigra of post-mortem PD brains (53). Therefore, subtypes of microglia in our model may secret regulatory signals or actively process and present antigenic material, potentially α-synuclein peptides (54), suggesting that the response we detected in culture could be further modified in vivo by signals from adaptive immunity or systemic inflammation (55).

An important finding is the identification of GPNMB as a key effector of microglial aggregate clearance. *GPNMB* is a PD GWAS candidate gene (56, 57) and one of the most strongly up-regulated genes in our microglial cluster 3 and 4. In postmortem brain, GPNMB immunoreactivity was increased specifically in microglia in the substantia nigra with α-synuclein pathology, in agreement with findings in animal models (58, 59). *GPNMB* gene knockout studies in animal models of α-synuclein pathology did not reveal a modifier action (60), whereas studies in iPSC-derived neurons suggested that loss of *GPNMB* prevents the uptake of α-synuclein fibrils and α-synuclein pathology (57). Our data show that GPNMB is highly expressed in microglia both in culture and in post-mortem brains, with minimal detection in neurons and it is required for efficient clearance of aggregates. Furthermore, *GPNMB* expression was regulated by an autocrine feedback loop involving IL-10 (fig. S10G). Thus, in agreement with our findings in post-mortem brains, our mechanistic investigations suggest a microglia-centric role for GPNMB in PD. Specifically, we found that GPNMB protein is minimally detected in DANs, and it is not up-regulated in neurons upon fibril exposure. Instead, GPNMB is upregulated in microglia when co-cultured with neurons previously seeded with fibrils. GPNMB was shown in other cell types to interact with extracellular matrix components such as syndecans (61) that were up-regulated in our model in neurons contacting MG. Although GPNMB is secreted under baseline conditions, potentially to modulate immune responses (62), we did not detect increased secretion when MG were co-cultured with fibril-seeded neurons. Using proximity ligation assay, we identified an interaction between GPNMB and pSyn within microglia. Thus, GPNMB is retained intracellularly upon binding to pSyn^+^ aggregates, facilitating lysosomal degradation, as previously shown in other contexts (63, 64), rather than contributing to aggregate spread (57). This underscores the importance of cell-type resolved and species-specific modelling in response to intraneuronal pathology when evaluating microglial gene function in complex diseases such as PD. Future experiments combining intracellular knockdown with extracellular GPNMB addition would help to more fully define the function of distinct GPNMB proteoforms.

There are limitations to our study. While our iPSC-based co-culture system enables dissection of human-specific neuron–microglia interactions, it does not fully recapitulate the multicellular and systemic complexity of the intact brain. Systemic factors may influence the full manifestation of pathogenic mutations and genetic modifiers in vivo. However, given well-established species differences in microglial biology, such human models provide a complementary and potentially more translationally relevant platform to interrogate disease mechanisms. Our modelling involved primarily DAN^TRIP^ and generalisation to sporadic synucleinopathies and different neuronal subtypes requires further investigation. In this regard, our findings in post-mortem human substantia nigra support a role for microglial GPNMB in sporadic PD.

In summary, our findings indicate that microglia deploy multiple regulatory checkpoints to fine-tune the trogocytosis of intraneuronal aggregates, revealing tractable therapeutic nodes in PD, including modulation of P2RY12 and GPNMB or suppression of IL-10 and CD22 signalling. Effective translation of these strategies will require accessible biomarkers capable of resolving microglial state transitions and guiding stage-specific intervention across the course of the disease.

## Materials and Methods

### Study Design

The objective of this study was to define mechanisms by which human microglia respond to and clear intraneuronal α-synuclein aggregates. Human iPSC-derived dopaminergic neurons and microglia were used in monoculture and co-culture systems to model endogenous α-synuclein aggregation caused by *SNCA* triplication or aggregation induced by exogenous α-synuclein fibrils. Experimental outcomes included quantification of α-synuclein aggregation, neuronal viability, microglial activation, transcriptomic changes, cytokine secretion, and aggregate clearance. Mechanistic studies were performed using pharmacological inhibitors, blocking antibodies, and inducible CRISPRi-mediated knockdown of candidate genes. Findings were assessed in postmortem human substantia nigra tissue from neurologically healthy controls, individuals with incidental Lewy body disease, and individuals with Parkinson’s disease (n=3 per group). Human iPSC lines and postmortem tissues were selected based on genotype and tissue availability and were not randomised. For in vitro experiments, treatment allocation was predetermined by experimental design. Investigators were blinded during image acquisition and data analysis. For single-cell RNA sequencing analyses, low-quality cells, ambient RNA–contaminated droplets, and doublets were excluded according to predefined quality-control criteria; no additional outliers were excluded. No statistical methods were used to predetermine sample size. Sample sizes were based on previous experience with iPSC differentiation experiments and functional assays. All experiments were independently replicated using multiple differentiations and, where indicated, independent iPSC lines. Biological replicate numbers are provided in the figure legends. Ethical approval for the use of human tissue, provided by the Oxford Brain Bank, was obtained from the Oxford C REC Ethics Committee (no. 15/SC/0639). Ethics Committees that approved the iPSC lines used in this study: for healthy control donors, National Health Service, Health Research Authority, NRES Committee South Central, Berkshire, UK, REC 10/H0505/71, and for SNCA patients REC 07/H0720/161. All experiments were performed in accordance with UK guidelines and regulations and as set out in the REC.

### Statistical Analysis

All statistical analyses were performed using GraphPad Prism 8 (GraphPad Software Inc., USA). Data were examined for normality, and appropriate statistical tests were selected accordingly. For normally distributed data, one-sample *t*-test, two-sided unpaired Student’s *t*-test or one-way ANOVA were used as shown. For non-normally distributed data, Wilcoxon Signed-Rank Test or the Kruskal–Wallis test was applied. The null hypothesis was rejected at a significance level of *P =* 0.05. Statistical significance in single cell analyses was assessed using Fisher’s Exact test, and multiple testing correction was applied using the false discovery rate (FDR) method. Only the top 20 terms based on fold enrichment score with FDR-adjusted P values < 0.05 were included. In experiments involving iPSC lines, biological replicates (*n*) were defined as one independent differentiation per iPSC line. Differentiations yielded either microglial monocultures, dopaminergic neuron monocultures, or co-cultures containing both cell types. Individual level data are presented in data file S1.

## Supplementary Material

Supplement

Supplementary data File S1

Table S2

Table S3

Table S4

Table S5

## Data Availability

All data needed to evaluate the conclusions in the study are presented in the paper and the Supplementary Materials. RNASeq data were deposited to GEO under the accession numbers GSE290525, GSE299790 and GSE299369. The anti-TREM2 antibodies used in this study were made available under a Material transfer Agreement with Eli Lilly. Requests for the dox inducible dCas9-KRAB iPSC line and other resources or reagents should be directed to and will be fulfilled by the lead contact, subject to completion of the required institutional material transfer agreement. All other iPSC lines are available from EBiSC. All other materials are commercially available or will be made available upon reasonable request.

## One-sentence Summary

Microglia clear intraneuronal α-synuclein aggregates

## References

[1] Tofaris GK (2022). Initiation and progression of α-synuclein pathology in Parkinson’s disease. Cellular and molecular life sciences.

[2] Zenko D, Marsh J, Castle AR, Lewin R, Fischer R, Tofaris GK (2023). Monitoring α-synuclein ubiquitination dynamics reveals key endosomal effectors mediating its trafficking and degradation. Science Advances.

[3] Wong YC, Krainc D (2017). α-synuclein toxicity in neurodegeneration: mechanism and therapeutic strategies. Nature medicine.

[4] Lee H-J, Patel S, Lee S-J (2005). Intravesicular localization and exocytosis of α-synuclein and its aggregates. Journal of Neuroscience.

[5] Choi I, Zhang Y, Seegobin SP, Pruvost M, Wang Q, Purtell K, Zhang B, Yue Z (2020). Microglia clear neuron-released α-synuclein via selective autophagy and prevent neurodegeneration. Nature communications.

[6] Krasemann S, Madore C, Cialic R, Baufeld C, Calcagno N, El Fatimy R, Beckers L, O’loughlin E, Xu Y, Fanek Z (2017). The TREM2-APOE pathway drives the transcriptional phenotype of dysfunctional microglia in neurodegenerative diseases. Immunity.

[7] Kim C, Ho D-H, Suk J-E, You S, Michael S, Kang J, Joong Lee S, Masliah E, Hwang D, Lee H-J (2013). Neuron-released oligomeric α-synuclein is an endogenous agonist of TLR2 for paracrine activation of microglia. Nature communications.

[8] Flury A, Aljayousi L, Park H-J, Khakpour M, Mechler J, Aziz S, McGrath JD, Deme P, Sandberg C, Ibáñez FG (2024). A neurodegenerative cellular stress response linked to dark microglia and toxic lipid secretion. Neuron.

[9] Zhang W, Wang T, Pei Z, Miller DS, Wu X, Block ML, Wilson B, Zhang W, Zhou Y, Hong J-S (2005). Aggregated α-synuclein activates microglia: a process leading to disease progression in Parkinson’s disease. The FASEB Journal.

[10] Pluvinage JV, Haney MS, Smith BA, Sun J, Iram T, Bonanno L, Li L, Lee DP, Morgens DW, Yang AC (2019). CD22 blockade restores homeostatic microglial phagocytosis in ageing brains. Nature.

[11] Keren-Shaul H, Spinrad A, Weiner A, Matcovitch-Natan O, Dvir-Szternfeld R, Ulland TK, David E, Baruch K, Lara-Astaiso D, Toth B (2017). A unique microglia type associated with restricting development of Alzheimer’s disease. Cell.

[12] Chiu IM, Morimoto ET, Goodarzi H, Liao JT, O’Keeffe S, Phatnani HP, Muratet M, Carroll MC, Levy S, Tavazoie S (2013). A neurodegeneration-specific gene-expression signature of acutely isolated microglia from an amyotrophic lateral sclerosis mouse model. Cell reports.

[13] Dolan M-J, Therrien M, Jereb S, Kamath T, Gazestani V, Atkeson T, Marsh SE, Goeva A, Lojek NM, Murphy S (2023). Exposure of iPSC-derived human microglia to brain substrates enables the generation and manipulation of diverse transcriptional states in vitro. Nature Immunology.

[14] Sun N, Victor MB, Park YP, Xiong X, Scannail AN, Leary N, Prosper S, Viswanathan S, Luna X, Boix CA (2023). Human microglial state dynamics in Alzheimer’s disease progression. Cell.

[15] Huang Y, Happonen KE, Burrola PG, O’Connor C, Hah N, Huang L, Nimmerjahn A, Lemke G (2021). Microglia use TAM receptors to detect and engulf amyloid β plaques. Nature immunology.

[16] Gosselin D, Skola D, Coufal NG, Holtman IR, Schlachetzki JC, Sajti E, Jaeger BN, O’Connor C, Fitzpatrick C, Pasillas MP (2017). An environment-dependent transcriptional network specifies human microglia identity. Science.

[17] Geirsdottir L, David E, Keren-Shaul H, Weiner A, Bohlen SC, Neuber J, Balic A, Giladi A, Sheban F, Dutertre C-A (2019). Cross-species single-cell analysis reveals divergence of the primate microglia program. Cell.

[18] Scheiblich H, Eikens F, Wischhof L, Opitz S, Jüngling K, Cserép C, Schmidt SV, Lambertz J, Bellande T, Pósfai B (2024). Microglia rescue neurons from aggregate-induced neuronal dysfunction and death through tunneling nanotubes. Neuron.

[19] Haenseler W, Zambon F, Lee H, Vowles J, Rinaldi F, Duggal G, Houlden H, Gwinn K, Wray S, Luk KC (2017). Excess α-synuclein compromises phagocytosis in iPSC-derived macrophages. Scientific reports.

[20] Tanudjojo B, Shaikh SS, Fenyi A, Bousset L, Agarwal D, Marsh J, Zois C, Heman-Ackah S, Fischer R, Sims D (2021). Phenotypic manifestation of α-synuclein strains derived from Parkinson’s disease and multiple system atrophy in human dopaminergic neurons. Nature communications.

[21] Haenseler W, Sansom SN, Buchrieser J, Newey SE, Moore CS, Nicholls FJ, Chintawar S, Schnell C, Antel JP, Allen ND (2017). A highly efficient human pluripotent stem cell microglia model displays a neuronal-co-culture-specific expression profile and inflammatory response. Stem cell reports.

[22] Welch JD, Kozareva V, Ferreira A, Vanderburg C, Martin C, Macosko EZ (2019). Single-cell multi-omic integration compares and contrasts features of brain cell identity. Cell.

[23] Wang X, Park J, Susztak K, Zhang NR, Li M (2019). Bulk tissue cell type deconvolution with multi-subject single-cell expression reference. Nature communications.

[24] Butovsky O, Jedrychowski MP, Moore CS, Cialic R, Lanser AJ, Gabriely G, Koeglsperger T, Dake B, Wu PM, Doykan CE (2014). Identification of a unique TGF-β–dependent molecular and functional signature in microglia. Nature neuroscience.

[25] Mittelbronn M, Dietz K, Schluesener H, Meyermann R (2001). Local distribution of microglia in the normal adult human central nervous system differs by up to one order of magnitude. Acta neuropathologica.

[26] Fujiwara H, Hasegawa M, Dohmae N, Kawashima A, Masliah E, Goldberg MS, Shen J, Takio K, Iwatsubo T (2002). α-Synuclein is phosphorylated in synucleinopathy lesions. Nature cell biology.

[27] Jonikas M, Madill M, Mathy A, Zekoll T, Zois CE, Wigfield S, Kurzawa-Akanbi M, Browne C, Sims D, Chinnery PF, Cowley SA (2018). Stem cell modeling of mitochondrial parkinsonism reveals key functions of OPA1. Ann Neurol.

[28] Lee H, Flynn R, Sharma I, Haberman E, Carling PJ, Nicholls FJ, Stegmann M, Vowles J, Haenseler W, Wade-Martins R (2020). LRRK2 is recruited to phagosomes and co-recruits RAB8 and RAB10 in human pluripotent stem cell-derived macrophages. Stem Cell Reports.

[29] Fernandes HJ, Hartfield EM, Christian HC, Emmanoulidou E, Zheng Y, Booth H, Bogetofte H, Lang C, Ryan BJ, Sardi SP (2016). ER stress and autophagic perturbations lead to elevated extracellular α-synuclein in GBA-N370S Parkinson’s iPSC-derived dopamine neurons. Stem cell reports.

[30] Kim W-G, Mohney RP, Wilson B, Jeohn G-H, Liu B, Hong J-S (2000). Regional difference in susceptibility to lipopolysaccharide-induced neurotoxicity in the rat brain: role of microglia. Journal of Neuroscience.

[31] Rueda-Carrasco J, Sokolova D, Lee SE, Childs T, Jurčáková N, Crowley G, De Schepper S, Ge JZ, Lachica JI, Toomey CE (2023). Microglia-synapse engulfment via PtdSer-TREM2 ameliorates neuronal hyperactivity in Alzheimer's disease models. The EMBO journal.

[32] Lill CM, Rengmark A, Pihlstrøm L, Fogh I, Shatunov A, Sleiman PM, Wang LS, Liu T, Lassen CF, Meissner E (2015). The role of TREM2 R47H as a risk factor for Alzheimer's disease, frontotemporal lobar degeneration, amyotrophic lateral sclerosis, and Parkinson's disease. Alzheimer's & Dementia.

[33] Rayaprolu S, Mullen B, Baker M, Lynch T, Finger E, Seeley WW, Hatanpaa KJ, Lomen-Hoerth C, Kertesz A, Bigio EH (2013). TREM2 in neurodegeneration: evidence for association of the p. R47H variant with frontotemporal dementia and Parkinson’s disease. Molecular neurodegeneration.

[34] Haynes SE, Hollopeter G, Yang G, Kurpius D, Dailey ME, Gan W-B, Julius D (2006). The P2Y12 receptor regulates microglial activation by extracellular nucleotides. Nature neuroscience.

[35] Pósfai B, Szabadits E, Cserép C, Vida S, Schwarcz AD, Fekete R, Tóth K, Bánkövi O, Szekeres-Paraczky C, Maglóczky Z (2026). P2Y12 Receptor Function Governs Microglial Surveillance and Cell–Cell Interactions in the Cerebral Cortex. Glia.

[36] Shin D-I, Banning U, Kim Y-M, Verheyen J, Hannen M, Bönig H, Körholz D (1999). Interleukin 10 inhibits TNF-alpha production in human monocytes independently of interleukin 12 and interleukin 1 beta. Immunological investigations.

[37] Duffy MF, Collier TJ, Patterson JR, Kemp CJ, Luk KC, Tansey MG, Paumier KL, Kanaan NM, Fischer DL, Polinski NK (2018). Lewy body-like alpha-synuclein inclusions trigger reactive microgliosis prior to nigral degeneration. Journal of neuroinflammation.

[38] Tofaris GK, Reitböck PG, Humby T, Lambourne SL, O’Connell M, Ghetti B, Gossage H, Emson PC, Wilkinson LS, Goedert M (2006). Pathological changes in dopaminergic nerve cells of the substantia nigra and olfactory bulb in mice transgenic for truncated human α-synuclein (1–120): implications for Lewy body disorders. Journal of Neuroscience.

[39] Schulz-Schaeffer WJ (2010). The synaptic pathology of α-synuclein aggregation in dementia with Lewy bodies, Parkinson’s disease and Parkinson’s disease dementia. Acta neuropathologica.

[40] Weinhard L, di Bartolomei G, Bolasco G, Machado P, Schieber NL, Neniskyte U, Exiga M, Vadisiute A, Raggioli A, Schertel A, Schwab Y (2018). Microglia remodel synapses by presynaptic trogocytosis and spine head filopodia induction. Nature communications.

[41] Nimmervoll B, White R, Yang J-W, An S, Henn C, Sun J-J, Luhmann HJ (2013). LPS-induced microglial secretion of TNFα increases activity-dependent neuronal apoptosis in the neonatal cerebral cortex. Cerebral Cortex.

[42] Lee H-J, Suk J-E, Bae E-J, Lee S-J (2008). Clearance and deposition of extracellular α-synuclein aggregates in microglia. Biochemical and biophysical research communications.

[43] Scheiblich H, Dansokho C, Mercan D, Schmidt SV, Bousset L, Wischhof L, Eikens F, Odainic A, Spitzer J, Griep A (2021). Microglia jointly degrade fibrillar alpha-synuclein cargo by distribution through tunneling nanotubes. Cell.

[44] Hakim-Mishnaevski K, Flint-Brodsly N, Shklyar B, Levy-Adam F, Kurant E (2019). Glial phagocytic receptors promote neuronal loss in adult Drosophila brain. Cell Reports.

[45] Lopes KdP, Snijders GJ, Humphrey J, Allan A, Sneeboer MA, Navarro E, Schilder BM, Vialle RA, Parks M, Missall R (2022). Genetic analysis of the human microglial transcriptome across brain regions, aging and disease pathologies. Nature genetics.

[46] Qin X-Y, Zhang S-P, Cao C, Loh YP, Cheng Y (2016). Aberrations in peripheral inflammatory cytokine levels in Parkinson disease: a systematic review and meta-analysis. JAMA neurology.

[47] Cockey SG, McFarland KN, Koller EJ, Brooks MM, Gonzalez De La Cruz E, Cruz PE, Ceballos-Diaz C, Rosario AM, Levites YR, Borchelt DR (2021). Il-10 signaling reduces survival in mouse models of synucleinopathy. npj Parkinson’s Disease.

[48] Beach TG, Sue LI, Walker DG, Lue LF, Connor DJ, Caviness JN, Sabbagh MN, Adler CH (2007). Marked microglial reaction in normal aging human substantia nigra: correlation with extraneuronal neuromelanin pigment deposits. Acta neuropathologica.

[49] Heneka MT, Kummer MP, Latz E (2014). Innate immune activation in neurodegenerative disease. Nature Reviews Immunology.

[50] Zhou Y, Song WM, Andhey PS, Swain A, Levy T, Miller KR, Poliani PL, Cominelli M, Grover S, Gilfillan S (2020). Human and mouse single-nucleus transcriptomics reveal TREM2-dependent and TREM2-independent cellular responses in Alzheimer’s disease. Nature medicine.

[51] Stevens B, Allen NJ, Vazquez LE, Howell GR, Christopherson KS, Nouri N, Micheva KD, Mehalow AK, Huberman AD, Stafford B (2007). The classical complement cascade mediates CNS synapse elimination. Cell.

[52] Le Guen Y, Luo G, Ambati A, Damotte V, Jansen I, Yu E, Nicolas A, De Rojas I, Peixoto Leal T, Miyashita A (2023). Multiancestry analysis of the HLA locus in Alzheimer’s and Parkinson’s diseases uncovers a shared adaptive immune response mediated by HLA-DRB1* 04 subtypes. Proceedings of the National Academy of Sciences.

[53] McGeer PL, Itagaki S, Boyes BE, McGeer E (1988). Reactive microglia are positive for HLA-DR in the substantia nigra of Parkinson's and Alzheimer's disease brains. Neurology.

[54] Sulzer D, Alcalay RN, Garretti F, Cote L, Kanter E, Agin-Liebes J, Liong C, McMurtrey C, Hildebrand WH, Mao X (2017). T cells from patients with Parkinson’s disease recognize α-synuclein peptides. Nature.

[55] Harms AS, Yang Y-T, Tansey MG (2023). Central and peripheral innate and adaptive immunity in Parkinson’s disease. Science translational medicine.

[56] Nalls MA, Blauwendraat C, Vallerga CL, Heilbron K, Bandres-Ciga S, Chang D, Tan M, Kia DA, Noyce AJ, Xue A (2019). Identification of novel risk loci, causal insights, and heritable risk for Parkinson's disease: a meta-analysis of genome-wide association studies. The Lancet Neurology.

[57] Diaz-Ortiz ME, Seo Y, Posavi M, Carceles Cordon M, Clark E, Jain N, Charan R, Gallagher MD, Unger TL, Amari N (2022). GPNMB confers risk for Parkinson’s disease through interaction with α-synuclein. Science.

[58] Moloney EB, Moskites A, Ferrari EJ, Isacson O, Hallett PJ (2018). The glycoprotein GPNMB is selectively elevated in the substantia nigra of Parkinson's disease patients and increases after lysosomal stress. Neurobiology of disease.

[59] Smajić S, Prada-Medina CA, Landoulsi Z, Ghelfi J, Delcambre S, Dietrich C, Jarazo J, Henck J, Balachandran S, Pachchek S (2022). Single-cell sequencing of human midbrain reveals glial activation and a Parkinson-specific neuronal state. Brain.

[60] Brendza R, Lin H, Stark K, Foreman O, Tao J, Pierce A, Ngu H, Shen K, Easton AE, Bhangale T (2021). Genetic ablation of Gpnmb does not alter synuclein-related pathology. Neurobiology of Disease.

[61] Shikano S, Bonkobara M, Zukas PK, Ariizumi K (2001). Molecular cloning of a dendritic cell-associated transmembrane protein, DC-HIL, that promotes RGD-dependent adhesion of endothelial cells through recognition of heparan sulfate proteoglycans. Journal of Biological Chemistry.

[62] Ripoll VM, Irvine KM, Ravasi T, Sweet MJ, Hume DA (2007). Gpnmb is induced in macrophages by IFN-γ and lipopolysaccharide and acts as a feedback regulator of proinflammatory responses. The Journal of Immunology.

[63] Wallings RL, Gillett DA, Staley HA, Mahn S, Mark J, Neighbarger N, Kordasiewicz H, Hirst WD, Tansey MG (2025). ASO-mediated knock-down of GPNMB in mutant-GRN and in Grn-deficient peripheral myeloid cells disrupts lysosomal function and immune responses. Molecular Neurodegeneration.

[64] Liu M, Zhu J, Zheng J, Han X, Jiang L, Tong X, Ke Y, Guo Z, Huang W, Cong J, Liu M (2025). GPNMB and ATP6V1A interact to mediate microglia phagocytosis of multiple types of pathological particles. Cell Reports.

[65] Sabate-Soler S, Nickels SL, Saraiva C, Berger E, Dubonyte U, Barmpa K, Lan YJ, Kouno T, Jarazo J, Robertson G (2022). Microglia integration into human midbrain organoids leads to increased neuronal maturation and functionality. Glia.

[66] Horlbeck MA, Gilbert LA, Villalta JE, Adamson B, Pak RA, Chen Y, Fields AP, Park CY, Corn JE, Kampmann M (2016). Compact and highly active next-generation libraries for CRISPR-mediated gene repression and activation. elife.

[67] Soneson C, Love MI, Robinson MD (2016). Differential analyses for RNA-seq: transcript-level estimates improve gene-level inferences. F1000Research.

[68] Lun AT, Riesenfeld S, Andrews T, Dao TP, Gomes T, Marioni JC, P. i. t. s. H. C. A. Jamboree (2019). EmptyDrops: distinguishing cells from empty droplets in droplet-based single-cell RNA sequencing data. Genome biology.

[69] Yang S, Corbett SE, Koga Y, Wang Z, Johnson WE, Yajima M, Campbell JD (2020). Decontamination of ambient RNA in single-cell RNA-seq with DecontX. Genome biology.

[70] Germain P, Lun A, Garcia Meixide C, Macnair W, Robinson M (2022). Doublet identification in single-cell sequencing data using scDblFinder. F1000Research.

[71] Hao Y, Stuart T, Kowalski MH, Choudhary S, Hoffman P, Hartman A, Srivastava A, Molla G, Madad S, Fernandez-Granda C (2024). Dictionary learning for integrative, multimodal and scalable single-cell analysis. Nature biotechnology.

[72] Ge SX, Jung D, Yao R (2020). ShinyGO: a graphical gene-set enrichment tool for animals and plants. Bioinformatics.

[73] Aran D, Looney AP, Liu L, Wu E, Fong V, Hsu A, Chak S, Naikawadi RP, Wolters PJ, Abate AR (2019). Reference-based analysis of lung single-cell sequencing reveals a transitional profibrotic macrophage. Nature immunology.

[74] Traag VA, Waltman L, Van Eck NJ (2019). From Louvain to Leiden: guaranteeing well-connected communities. Scientific reports.

[75] Finak G, McDavid A, Yajima M, Deng J, Gersuk V, Shalek AK, Slichter CK, Miller HW, McElrath MJ, Prlic M (2015). MAST: a flexible statistical framework for assessing transcriptional changes and characterizing heterogeneity in single-cell RNA sequencing data. Genome biology.

[76] Tuddenham JF, Taga M, Haage V, Marshe VS, Roostaei T, White C, Lee AJ, Fujita M, Khairallah A, Zhang Y (2024). A cross-disease resource of living human microglia identifies disease-enriched subsets and tool compounds recapitulating microglial states. Nature Neuroscience.

[77] Miller SA, Policastro RA, Sriramkumar S, Lai T, Huntington TD, Ladaika CA, Kim D, Hao C, Zentner GE, O’Hagan HM (2021). LSD1 and aberrant DNA methylation mediate persistence of enteroendocrine progenitors that support BRAF-mutant colorectal cancer. Cancer research.

[78] González-Velasco Ó, Simon M, Yilmaz R, Parlato R, Weishaupt J, Imbusch CD, Brors B (2024). Identifying similar populations across independent single cell studies without data integration. bioRxiv.

[79] Street K, Risso D, Fletcher RB, Das D, Ngai J, Yosef N, Purdom E, Dudoit S (2018). Slingshot: cell lineage and pseudotime inference for single-cell transcriptomics. BMC genomics.

[80] Van den Berge K, Roux de Bézieux H, Street K, Saelens W, Cannoodt R, Saeys Y, Dudoit S, Clement L (2020). Trajectory-based differential expression analysis for single-cell sequencing data. Nature communications.

