# Supplementary material for: Human microglia clear intraneuronal α-synuclein aggregates by GPNMB-mediated trogocytosis
Source: Sci Transl Med. Author manuscript; Available in PMC 2026 Oct 19. (PMC7619603; doi:10.1126/scitranslmed.adz9258)
Supplement: Supplementary file 1 [file EMS220162-supplement-Supplement.pdf]

Supplementary materials for

Human microglia clear intraneuronal α-synuclein aggregates by GPNMB-mediated trogocytosis

Hung-Ju Chueh *et al.*

Corresponding author: Prof. George K. Tofaris (george.tofaris@ndcn.ox.ac.uk)

This PDF file includes:

Supplementary Material and Methods

Fig. S1 to S10

Table S1, S6, S7, S8

SUPPLEMENTARY MATERIALS AND METHODS

iPSC maintenance and differentiation of dopaminergic neurons and microglia

All iPSC cell lines have been previously characterised as summarised in **Table S1**. iPSCs were maintained in mTesR1 media (Stem Cell Technologies, 95957) and were differentiated into dopaminergic neurons using a protocol developed by Kriks et al. as adapted in our lab by Tanudjojo *et al.*(*24*). In summary, iPSCs were seeded on Geltrex-coated six-well plates and expanded to >80% confluency. The media were then switched from mTeSR1 to floor plate induction media until DIV (day in vitro) 12, consisting of 2 μM A83-01 (Tocris, 2939), 100 nM LDN (Sigma, SML0559), 300 ng/ml Sonic Hedgehog (Shh – R&D Systems, 464-SH-200), 2 μM purmorphamine (Tocris, 4551), 200 ng/ml FGF8a (R&D Systems, 4745-F8), and 3 μM CHIR-99021 (Tocris, 4423). From DIV 12, media were switched to dopaminergic neural differentiation and maturation media, consisting of 20 ng/ml BDNF (PeproTech, 450–02), 20 ng/ml GDNF (PeproTech, 450–10), 1 ng/ml TGFβ3 (Life Technologies, PHG9305), 10 μM DAPT (Tocris, 2634), 200 μM ascorbic acid (Sigma, A4403), and 500 μM dbcAMP (Sigma, D0627). At DIV20, cells were replated onto Geltrex-coated plates or coverslips. Starting from DIV21, media changes incorporated DIV11-19 components until the designated analysis timepoints.

Human microglia-like cells were derived from iPSCs using an established protocol (*19*). In summary, iPSCs were transferred to AgreWell800 plates (Stem Cell Technologies, 34811) when cultured to >90% confluency, with Embryoid Body (EB) medium consisting of 50 ng/ml Vascular endothelial growth factor (VEGF – Invitrogen, PHC9394), 50 ng/ml Bone morphogenetic protein 4 (BMP4 – Invitrogen, PHC9534), and 20 ng/ml Stem cell factor (SCF – Milteny, 130-096-692). On day 7, T175 flasks with 15 ml 'factory' differentiation medium were prepared. EBs from each AggreWell were transferred into two T175 flasks. EB medium was replaced with factory medium consisting of the X-vivo 15 medium (Lonza, BE02-060F) and small molecules including 25 ng/ml Interleukin-3 (IL3 – Invitrogen, PHC0033) and 100 ng/ml Macrophage stimulating factor (M-CSF – Invitrogen, PHC9501). Factory medium was added weekly (10–15 ml), with macrophage/microglia precursors (MGpre) emerging around days 14–28. Harvested cells were cultured for up to 2 weeks either as microglia monocultures (MG) or in co-culture with DANs (MG-DAN) at a 1:1 ratio. It has been previously demonstrated that certain components of iPSC-DAN medium are directly toxic to iPS-microglia (dbcAMP, TGFβ3) (*65*), and that GM-CSF is optional for the differentiation of microglia (*19*), so

these were not included in the coculture medium, and so the mono/co-culture medium consisted of Advanced DMEM/F12 and 100 ng/ml of Interleukin-34 (IL-34 – Peprotech, 200–34).

Generation of dCas9-KRAB iPSC-derived microglia

dCas9-KRAB HC iPSCs were generated by lentiviral transduction of dCas9-KRAB and M2rtTA into the AAVS1 locus (S7). dCas9-KRAB iPSCs were transduced in suspension with sgRNA lentivirus targeting P2RY12 or GPNMB. Transduced iPSCs were then selected with 1 μg/ml puromycin (Sigma, P8833) for 5 days or until 90–95% confluency was reached. sgRNA sequences were obtained from the hCRISPRi-v2.1 Weissman Laboratory Library (*66*). Transduced iPSCs were differentiated into microglia as described previously and treated with 1.5 μg/ml doxycycline (Sigma, D3447) every 48h to maintain dCas9 expression and gene knockdown until the final timepoints.

α-Synuclein purification and fibril assembly

Generation of α-synuclein (αSyn) fibrils was carried out following the method we described previously (*20*). In summary, the expression construct pRK172, encoding recombinant wild-type human αSyn was transformed into BL21 (DE3) E.Coli ((New England BioLabs, C2527H). The transformed cells were grown on ampicillin LB agar plate overnight. All grown colonies were collected and transferred into Terrific broth media. The bacteria were allowed to expand for 2.5 hours while shaking, after which 0.4 mM isopropyl β-d-1-thiogalactopyranoside (IPTG, VWR, 437144N) was added to induce αSyn expression followed by 5 hours of further incubation. Bacteria were then harvested and resuspended in low salt buffer [50 mM Tris-HCl, pH 7.4 (Santa Cruz Biotechnology, sc-301950), 20 mM NaCl (Sigma, S9888), 1 mM EDTA, 0.1 mM DTT, 0.1 mM PMSF (Calbiochem, 52332), and 2x cOmplete Protease Inhibitor Cocktail tablets (Roche, 04693116001)] containing 1 mg/ml lysozyme (Thermo Scientific, 89833), DNase (Invitrogen, 18047019), and RNase (Thermo Scientific, EN0601). Cells were lysed by sonication on ice (5 seconds on/5 seconds off, 40% amplitude) for 2 minutes. Cell debris were removed by centrifuging at 15,000 x g for 20 minutes at 4°C. Supernatant was collected and purified using a two-stage high-performance liquid chromatography. Supernatant was fractionated with a HiTrap Q HP anion exchange chromatography column (Cytiva, 17115401) followed by a HiLoad 16/600 Superdex 75 pg size exclusion chromatography column (Cytiva, 28-

9893-33). Fractions enriched in αSyn, identified by SDS-PAGE Coomassie stain and immunoblotting, were pooled and spin-concentrated at 400g for 8–12 hours at 4°C using a Vivaspin column (Cytiva, 28-9323-58). Buffer exchange was then done using a 50 mM/150 mM KCL solution. The concentration of the purified αSyn was measured using Pierce BCA assay kit (Thermo Scientific, 23225) per manufacturer's instructions and NanoDrop spectrophotometer (Thermo Scientific, ND-ONE-W) and diluted to 350 μM (5 mg/ml). The purified α-synuclein were assembled into fibrils via 1000 rpm rotational shaking at 37°C for 7 days. Fibrils were diluted to a working concentration of 1 μM followed by sonication at 20% amplitude for 2mins before added to cell media. Endotoxin levels at 1× dilution, or in the conditioned media of seeded neurons, were assessed using the Pierce Chromogenic Endotoxin Quant Kit (Thermo Fisher, A39553).

Transmission electron microscopy

Transmission electron microscopy was used to characterise the morphology of αSyn fibrils. Fibril were deposited onto freshly glow-discharged, carbon-coated 300 mesh copper grids (TAAB Laboratories, C267/050) and incubated for 2 minutes, blotted with filter paper and stained with 2% uranyl acetate for 10 seconds, blotted gently, and air-dried. Imaging was performed using a JEOL 1400 TEM operating at 120 kV, equipped with a Gatan Rio CMOS camera. Fibril length quantification post sonication was carried out in ImageJ51.

Thioflavin T assay

Fibril generation was monitored using Thioflavin T assay as instructed by the manufacturer. Briefly, samples taken over a period of 1 to14 days of shaking were mixed with 25μM ThT in PBS followed by 1hour incubation in the dark at 37°C and subsequently analysed using a CLARIOstar Plus microplate reader with excitation at 450 nm and emission at 485 nm. Fluorescence intensity values were normalised against αSyn monomer to calculate the fold change in aggregation over time.

Seeding of cells with exogenous αSyn fibrils

Before experimental use, de novo generated fibrils were tested in a HEK293 cell line overexpressing human αSyn-Venus fusion protein via lentiviral transduction and G418 antibiotic selection. HEK293 cells were

cultured in DMEM/F-12 (Gibco, 11320033) supplemented with 10% FBS (Sigma, F7524), 1% GlutaMAX (Gibco, 35050061), and 1% Penicillin/Streptomycin (Gibco, 15140122). Fibrils were sonicated diluted to 1 μM in cell media and sonicated for 2 minutes (1s on/1s off, 20% amplitude) before adding to HEK-mVenus cells for 6 hours using Lipofectamine 2000 (Invitrogen, 17504044). Imaging was then performed using an EVOS FL fluorescent microscope (Invitrogen, AMF4300) to confirm the presence of fluorescent punctate, indicating αSyn aggregation. Lysates were than collected 24 hours post-transfection followed by immunoblotting. A similar protocol was adapted for adding fibrils to neuronal or microglial cultures, but without the use of lipofectamine in their corresponding culture media.

Bulk RNA sequencing

RNA samples from neuronal monocultures and neuron-microglia co-cultures were prepared using standard poly-A enrichment, 250–300 bp insert cDNA library was prepared with the NEB Next Ultra RNA Library Prep Kit (New England Biolabs, E7530) and sequenced on Illumina NovaSeq 6000 (S4) using 150 bp paired-end reads. Sequencing resulted in ~40 million reads per sample. Filtered substantia nigra single nuclei count data with cell type annotations for 7 post-mortem individuals was downloaded from the Broad Institute Single Cell Portal [https://singlecell.broadinstitute.org/single_cell]. The count matrices for all individuals were merged with 24 cell types encompassing all known resident cell classes as identified previously in the literature (*22*). For cell type deconvolution analysis, these cell types were merged into eight major subtypes: astrocytes, dopaminergic neurons, endothelial, excitatory and inhibitory neurons, microglia, oligodendrocytes and oligodendrocyte progenitor cells endothelial, excitatory and inhibitory neurons, microglia, oligodendrocytes and oligodendrocyte progenitor cells. Kallisto (v0.46.2) was used to estimate abundance for Ensembl (release 88) protein-coding genes from RNAseq reads. The cell-type composition of the bulk RNA-Seq samples was characterised using the MuSiC method (*23*).

To visualise the expression profiles of cell-type and stress-related genes across different experimental conditions, we generated heatmaps using normalised transcriptomic data. Transcript-level abundance estimates (TPM) were imported using the tximport R package (*67*). Gene-level TPM values were extracted by filtering for NSC-specific samples using consistent naming conventions. Metadata, including tissue type and

replicate information, was annotated using sample identifiers. Gene sets were compiled based on prior literature and publicly available datasets. These included markers associated with oxidative, ER, hypoxia, mitochondrial, and metabolic stress. TPM values were log2-transformed and z-scored (row-scaled) across each gene to emphasize relative expression differences while accounting for baseline variability. Genes with zero variance across all samples were excluded from visualization. Heatmaps were generated in R, with annotation metadata overlaid as sample labels. To maintain interpretability, only non-redundant gene symbols were retained, and duplicates were removed based on gene names.

Single cell RNA sequencing

Cell suspensions were enzymatically dissociated using Accutase to achieve a single-cell state. Following dissociation, cells were fixed in 100% methanol in accordance with the protocol recommended by 10x Genomics. Cells were loaded into the Chromium Controller (10x Genomics) for microfluidic partitioning and barcoding. Library preparation was performed using the Chromium Single Cell 3' Reagent Kit v3 (10x Genomics, PN-1000268), following the manufacturer's protocol. Sequencing was conducted on the Illumina NovaSeq 6000 platform. CellRanger count pipeline was used to process FASTQ files, generating feature-barcode matrices. Cells were identified using DropletUtils::emptyDrops (*68*). Those with high-ambient RNA contamination were identified and removed using decontX (*69*) and doublets were filtered out using scDblFinder (*70*). Low-ambient singlets were further processed and analysed primarily using Seurat V5 (*71*), with additional packages used as specified. Biological replicates were integrated using Seurat::CCAIntegration.

<u>Transcriptomic profiling of Neurons in monoculture and co-culture</u>

To investigate whether clearance of aggregated proteins is associated with neuronal rescue, we performed single-cell RNA sequencing (scRNA-seq) on dopaminergic neurons (DANs) seeded with fibrils alone (monoculture) and fibril-seeded DANs co-cultured with microglia (MG). Processed Seurat objects corresponding to different experimental groups were merged and annotated to distinguish monoculture and co-culture conditions. After merging, highly variable genes were identified using Seurat::FindVariableFeatures with the 'vst' method (n = 2000). The data were scaled, and principal component

analysis (PCA) was performed. Clustering was carried out using Seurat::FindNeighbors and FindClusters with a resolution of 0.1, followed by dimensionality reduction with UMAP (dims = 1:30).

Nine transcriptionally distinct clusters were identified. Clusters 0, 1, 2, 4, 6, and 7 were annotated as neurons based on the expression of canonical neuronal and dopaminergic markers. Cluster 3 was annotated as microglia. We then focused on neuronal clusters to examine expression signatures associated with various cellular stress responses. Expression levels for these gene sets were visualised using Seurat::DotPlot. Values were log-normalised and z-score transformed across genes. Dot plots were generated to show expression intensity and the proportion of cells expressing each gene. Differential gene expression analysis was performed using Seurat::FindAllMarkers with the Wilcoxon rank-sum test (test.use = "wilcox") and parameters set to logfc.threshold = 0.25 and min.pct = 0.1. Gene Ontology (GO) and pathway enrichment analyses were conducted using ShinyGO (v0.77) (*72*). Only differentially expressed genes between monoculture and co-culture groups were included. GO terms with FDR-adjusted P values < 0.05 were considered significant.

Trajectory Inference and Functional Annotation of Microglia

Cells were annotated by mapping to reference dataset from Welch et al. (*26*) using SingleR (*73*) and Seurat::TransferData as a complementary method. Only microglia cells (N=5640) confidently annotated by both methods were used for further analysis. Microglia cells were re-integrated using Seurat::RPCAIntegration, followed by clustering using the Leiden algorithm (resolution 0.4) (*74*). To identify top differentially expressed genes for each cluster, pairwise testing with MAST was performed (*75*) adapting code from Tuddenham et al. (*76*) to summarise the results from all pairwise comparisons. Significance of differences in cluster proportions between non-seeded and fibril seeded conditions was determined using scProportionTest (*77*). Module scores for DAM genes were calculated using Seurat::AddModuleScore. Similarity of clusters to the microglia states defined by Dolan et al. (*13*) were determined using ClusterFoldSimilarity (*78*), which determines similarity by comparing fold changes of shared genes across clusters.

Trajectory was inferred using Slingshot (*79*) and generalized additive models were fitted using tradeSeq (*80*). Microglia cluster 2, with the highest proportion in non-seeded condition and the lowest DAM score among

the four largest microglia clusters, was specified as the starting cluster for the trajectory inference. To characterise each trajectory, Gene Ontology (GO): Cellular Component enrichment analysis was performed using the PANTHER Overrepresentation Test (Release 20240807) with the database (DOI: 10.5281/zenodo.15066566, released 2025-03-16), based on differentially expressed genes between the start and end clusters of each trajectory.

Homogenous time resolved fluorescence (HTRF)

αSyn aggregation was measured using an anti-phospho Ser129 αSyn HTRF kit (Revvity, 6FSYNPEG) following the manufacturer's instructions. 4 µl of donor and acceptor Förster Resonance Energy Transfer (FRET) fluorophores were mixed with 16 µl of cell lysate in a 96-well plate, sealed with a film and incubated for 2 hours in the dark. Fluorescence intensity was recorded using a CLARIOstar Plus Microplate Reader (BMG Labtech, Ortenberg, Germany) with excitation at 340 nm and emission readings at 620 nm and 665 nm. Time-resolved FRET was determined by calculating the emission ratio (665/620) followed by background subtraction, BCA normalisation, and fold increase over non-seeded control.

Immunoblotting

Cells were lysed in RIPA [50 mM Tris-HCl, pH 7.4 (Santa Cruz Biotechnology, sc-301950), 500 mM NaCl (Sigma, S9888), 0.1% SDS (Sigma, L3771), 5 mM EDTA (Invitrogen, 15575020), 1 mM DTT (Roche, 10708984001) and 1x protease inhibitor tablet (Roche, 11697498001) in dH_2O], followed by 10-minute centrifugation at 10,000g to remove cell debris. The lysate was then further spun down at 100,000g for 2 hours. The supernatant was collected as the RIPA soluble fraction and the pellet was resuspended in the same RIPA buffer with 2% SDS, sonicated for 2 minutes (1s on/1s off, 20% amplitude) and kept as the insoluble fraction for determining the level of pSyn.

Protein concentration was quantified by BCA following manufacturer's instructions and the lysates were then mixed with NuPAGE LDS Sample Buffer (Invitrogen, NP0007) and Sample Reducing Agent (Invitrogen, NP0009) and heated at 95°C for 5 minutes. Samples were resolved by 4–12% Bis-tris gels and transferred to a nitrocellulose membrane. Membranes were blocked for 1 hour in 5% bovine serum albumin (Melford, A30075) in 1X TBS with 0.1% Tween-20 (Sigma, P1379). Membranes were incubated overnight

at 4°C with primary antibodies, followed by a 1hour incubation at room temperature with HRP-conjugated secondary antibodies. Membranes were then incubated with ECL Start Western Blotting Detection Reagent (Cytiva, GERPN3244) for 1–5 minutes and imaged using a ChemiDoc Imaging System (Bio-Rad). Band intensities were quantified by densitometry using Image Lab software (Bio-Rad, v6.1). All antibodies and their dilutions are listed in **Table S7**.

Microscopy

Immunocytochemistry: Cells were fixed in 4% paraformaldehyde (PFA), permeabilised with 0.1% Triton-X-100, and blocked in 10% BSA in PBST (0.1% Tween-20) for 1 hour at RT. This was followed by overnight incubation with primary antibodies at 4°C and washed in DPBS three times for 15 minutes at RT the following day. Cells were then stained with fluorescently labelled secondary antibodies for 1 hour at RT, washed in DPBS three times for 5 minutes. For detection of cathepsin B activity, cells were incubated with Magic Red (Abcam, ab270772; 1:25 dilution of the prepared stock) for 30 minutes, followed by washing in DPBS two times, prior to imaging. Finally, samples were mounted in ProLong Glass Antifade Mountant with DAPI (Invitrogen, P36981) or VECTASHIELD Antifade Mounting Medium (Vector Laboratories, H-1000-10) and sealed with CoverGrip Coverslip Sealant (Biotium, 23005).

Immunohistochemistry for human brain sections: Antigen retrieval was performed by steaming slides in 10 mM sodium citrate buffer (pH 6.0, 0.05% Tween-20) for 30 minutes, followed by cooling at room temperature for 1 hour in retrieval buffer. Slides were then blocked with 5% BSA at room temperature. Primary antibodies [anti-GPNMB (1:200), antiIBA1 (1:200), anti-pSyn (1:500)] were applied and incubated overnight at 4°C. Slides were washed 3× in TBST, followed by incubation with secondary antibodies for 1 hour 30 minutes at room temperature. After washing 3× in TBST, autofluorescence was quenched using Autofluorescence Quenching Reagent for 10 minutes (ThermoFisher, R37630), followed by washes 2× in TBST and 1× in TBS. Samples were then mounted with ProLong Glass Antifade Mountant (Invitrogen, P36980).

Super-resolution microscopy: Imaging was performed using a Zeiss LSM980 laser scanning confocal microscope, integrated with an Airyscan 2 module, featuring an Olympus Plan-Apochromat 63x 1.43 NA oil-

immersion lens and a 32-channel gallium arsenide phosphide (GaAsP-PMT) area detector. Z-stack images were generated, with stack intervals at 0.13 μm, operated by a Wienecke & Sinske PiezoDrive CAN stage controller, post-processed by Zen Blue (V3 3.89). All Images are quantified in Fiji (ImageJ 3.5). For Super-resolved structured illumination microscopy (iSIM), VisiTech iSIM super-resolution module integrated with an Olympus IX83 inverted microscope, equipped with a high-resolution 100× UPlanApo TIRF oil immersion objective (NA 1.50). Detection was carried out using a Hamamatsu ORCA-Quest qCMOS camera. To enhance image contrast and spatial resolution while minimizing artefact introduction, raw image data were processed in cellSens Dimension software (Olympus, v4.2.1).

Proximity Ligation Assay

Proximity ligation assays (PLA) were performed using 13 mm 1.5H high-precision glass coverslips (Marienfeld, Cat. No. 0117530). All PLA procedures used the Duolink In Situ Red Starter Kit for Mouse/Rabbit (Sigma, DUO92101), following the manufacturer's instructions. In summary, cells were initially fixed with 4% PFA in DPBS for 15 minutes, then permeabilized for 30 minutes at room temperature using DPBS supplemented with 0.1% Triton X-100. After permeabilization, neurons were incubated with Duolink Blocking Solution at 37°C for one hour. Primary antibodies were diluted in Duolink Antibody Diluent and applied overnight at 4°C. The following day, two washes were performed using 1X Wash Buffer A, each for 10 minutes. PLA probes (PLUS and MINUS) were prepared at a 1:5 dilution in Antibody Diluent along with corresponding secondary antibodies. Co-cultures were incubated with this solution, followed by another two 10-minute washes in 1X Wash Buffer A. Subsequently, ligation was carried out by incubating in the Ligation Buffer containing ligase for 30 minutes at 37°C. After ligation, two further washes with Wash Buffer A were conducted before amplification using 1X Amplification Buffer for 100 minutes. Post-amplification, cells were washed twice in Wash Buffer B for 10 minutes each and finally rinsed in 0.01X Wash Buffer B for 1 minute.

Image quantification and 3D reconstruction

iPSC-Derived Cultures

Images were acquired using a Zeiss LSM 880 microscope or iSIM at specified magnifications (20×–63×, ≥3–10 fields/differentiation/cell line). All image analyses were performed in ImageJ/FIJI using consistent pipelines. To assess microglial morphology, masked microglia were analysed for circularity (4π×area/perimeter²), aspect ratio (major/minor axis), and roundness (4×area/(π×major axis²)) (, n≥5 fields/differentiation/cell line). To assess neuron–microglia interactions, neuronal and microglial markers were masked and thresholded using Fiji algorithms, and their interactions per stack were quantified using Mander's coefficient (n ≥ 5 cells /differentiation/cell line).To quantify pSyn or Synapsin I level, thresholding were applied, followed by "Analyse Particles" for particle number, % area, size; normalized to $MAP2^+$ neurons (n≥3 fields/differentiation/cell line).To measure $pSyn^+$ or $MAP2^+$ signal in microglia, $MAP2^+$ $pSyn^+$ areas were quantified as above in CD68+ ROIs. (n≥10 cells/differentiation/cell line).

For stained human postmortem tissue images, confocal z-stacks were subjected to background subtraction, automatic thresholding using Fiji algorithms, and JACoP plugin analysis to determine percentage voxel overlap. All three-dimensional reconstructions were performed in Imaris (version 10.0.0).

Relative gene expression

RNA extraction was carried out using the RNeasy Kit (Qiagen, 74104) following the guidelines provided by the manufacturer. A total of 1 µg of cDNA was generated using random hexamers provided in the QuantiTect Reverse Transcription Kit (Qiagen, 205311). Quantification of mRNA levels was done using real-time PCR, which involved a mixture containing 5 ng of cDNA, LightCycler 480 SYBR Green I Master Mix (Roche Life Science, 04707516001) and 1 µM of each primer, both forward and reverse. Normalisation of the data were done using the housekeeping gene GAPDH. The sequences of all primers used are shown in **Table S8**. Analysis of the samples was conducted using the LightCycler 480 Instrument (Roche Life Science). The instrument's software was utilised for the determination of crossing threshold (Ct) values. The relative expression of genes was then calculated using the ΔΔCt method.

MSD electrochemiluminescence

The R-plex human neurofilament L kit was used as per manufacturer's instruction (Meso Scale Discovery, F217X-3), using 25 µl of sample (diluted cell lysate at 1:50) or standard. Briefly, samples and standards were prepared and added to pre-coated assay plates for 1 hour at RT, followed by the addition of electrochemiluminescent-labelled detection antibodies. After 1 hour of incubation and washing to remove unbound substances, the assay was read using MESO QuickPlex SQ 120MM (Meso Scale Diagnostics, USA).

ELISA

α-Syn, TNFα, IL-10, and GPNMB levels in lysates and conditioned media from mono- and co-cultures were quantified using corresponding ELISA kits (BioLegend 448607, KHC3011, Life Technologies, BioLegend 430604, and Bio-Techne DY2550, respectively) following the manufacturers' protocols. For all assays, 96-well plates were coated with a capture antibody and incubated overnight at 4°C. The next day, 100 µl of either samples or standards were added to the wells and incubated for 2 hours. Following this, a detection antibody was applied and incubated for another 2 hours. After each step, the plates were washed three times with wash buffer. Subsequently, 100 µl of Avidin-HRP was added and incubated for 30 minutes, followed by the addition of TMB substrate solution for another 30 minutes. The reaction was terminated with 50 µl of stop solution. Absorbance was measured at 450 nm using a CLARIOstar Plus microplate reader (BMG Labtech, Germany). The following dilutions were used for α-Syn: 1/1000 for lysates from dopaminergic neuron and 1/100 for lysates from co-culture; for TNFα: 1/2 for conditioned media; for IL-10: 1/10 for monoculture-conditioned media and 1/1 for co-culture-conditioned media. For GPNMB, the dilutions were: 1/250 for dopaminergic neuron lysates, 1/1000 for co-culture lysates, 1/10 for dopaminergic neuron-conditioned media, and 1:1000 for co-culture-conditioned media.

Human cytokine array

Cytokines in conditioned media from co-cultured microglia and dopaminergic neurons were detected using the Proteome Profiler Human XL Cytokine Array Kit (R&D Systems, # ARY022B). Dopaminergic neurons were plated at 800,000 cells/well in 12-well plates, with microglia added at a 1:1 ratio; conditioned media were collected 2 weeks later, clarified by centrifugation at 1200 × g for 10 min at 4°C, and processed as per

the manufacturer's protocol. For biological replicates, media from three independent iPSC differentiations were pooled per condition onto duplicate-spotted nitrocellulose membranes, which were blocked, incubated overnight with samples at 4°C, then sequentially with detection antibody cocktail, streptavidin-HRP, and chemiluminescent reagents. Spot intensities were quantified using the ChemiDoc MP Imaging System (Bio-Rad).

Treatments with Antibodies, Inhibitors, LPS, and Annexin V

Antibodies targeting human IL-10 receptor-alpha (0.1 µM and 1 µM), CD22 (1 µM) or SPP1 (5 µg/ml) and the non-targeted controls (corresponding concentrations) or inhibitors targeting MCP-1 (#RS504393, 2 µM), MMP9 (#444278, 5 µM) or P2RY12 (PSB-0739, 2 µM and 4 µM) were added every 3 days to microglia monocultures or neuron-microglia co-cultures. A similar protocol was followed for the TREM2 antagonist and its corresponding IgG control, applied at a concentration of 10 µg/ml. Annexin V was added to neurons at a concentration of 0.2 µg/ml prior to co-culturing with microglia and subsequently re-applied every three days at 0.2 µg/ml until the end of the co-culture period. LPS was added two days after plating the microglia at a final concentration of 200 ng/ml and subsequently re-applied every three days until the end of the co-culture period.

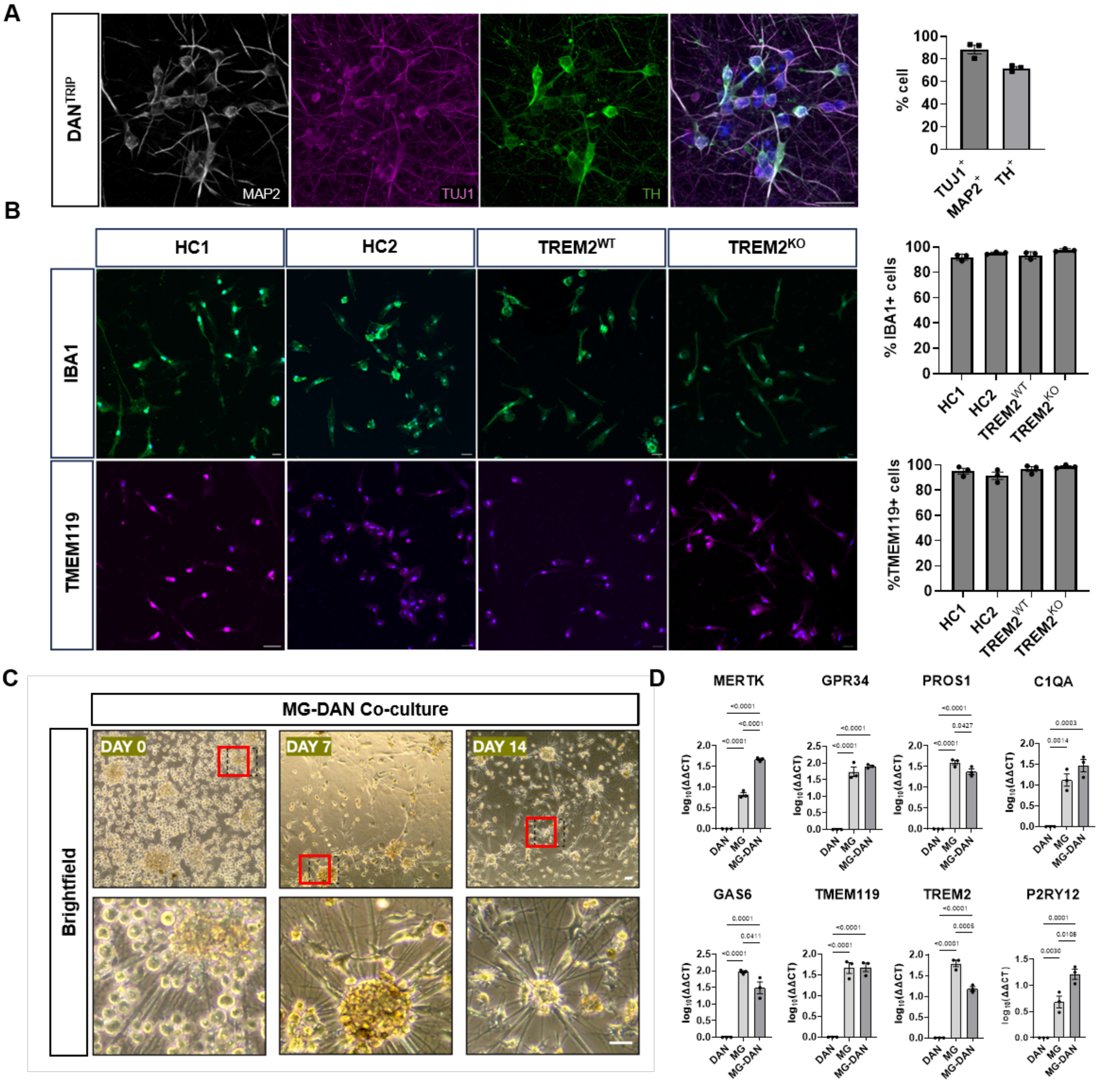

Fig. S1 Characterisation of iPSC-derived dopaminergic neurons and microglia in monoculture and co-culture.
(A) Representative confocal images and quantification of iPSC-derived dopaminergic neurons (DANs) from the DAN^{TRIP1} line, stained for TH, MAP2, and TUJ1 (n=3 independent differentiations for DAN^{TRIP1}).
(B) Representative confocal images and quantification of iPSC-derived microglia (MG) stained with IBA1 and TMEM119 from healthy controls (HC1, HC2), isogenic control (ISO), and TREM2 knockout (TREM2 KO) lines (n=3 differentiations per iPSC line).
(C) Brightfield images showing time-dependent morphological maturation of MG^{pre} in MG–DAN co-culture. Initially, pMacpre displayed a uniform round shape. After 7 days, cells adopted a ramified MG morphology among DANs under baseline conditions. The representative image of D14 MG-DAN co-culture is also shown in **Fig 1A**.
(D) qPCR quantification of MG gene expression in DAN^{TRIP} monocultures, MG monoculture, and MG-DAN^{TRIP} co-cultures (n=3 independent differentiations for DAN^{TRIP1}, MG^{HC1}, MG^{HC1}-DAN^{TRIP1}).

Data are presented as mean ± SD. Statistical analysis by one-way ANOVA **(D)** with Tukey's post hoc test. *Scale bar*: 20 µm.

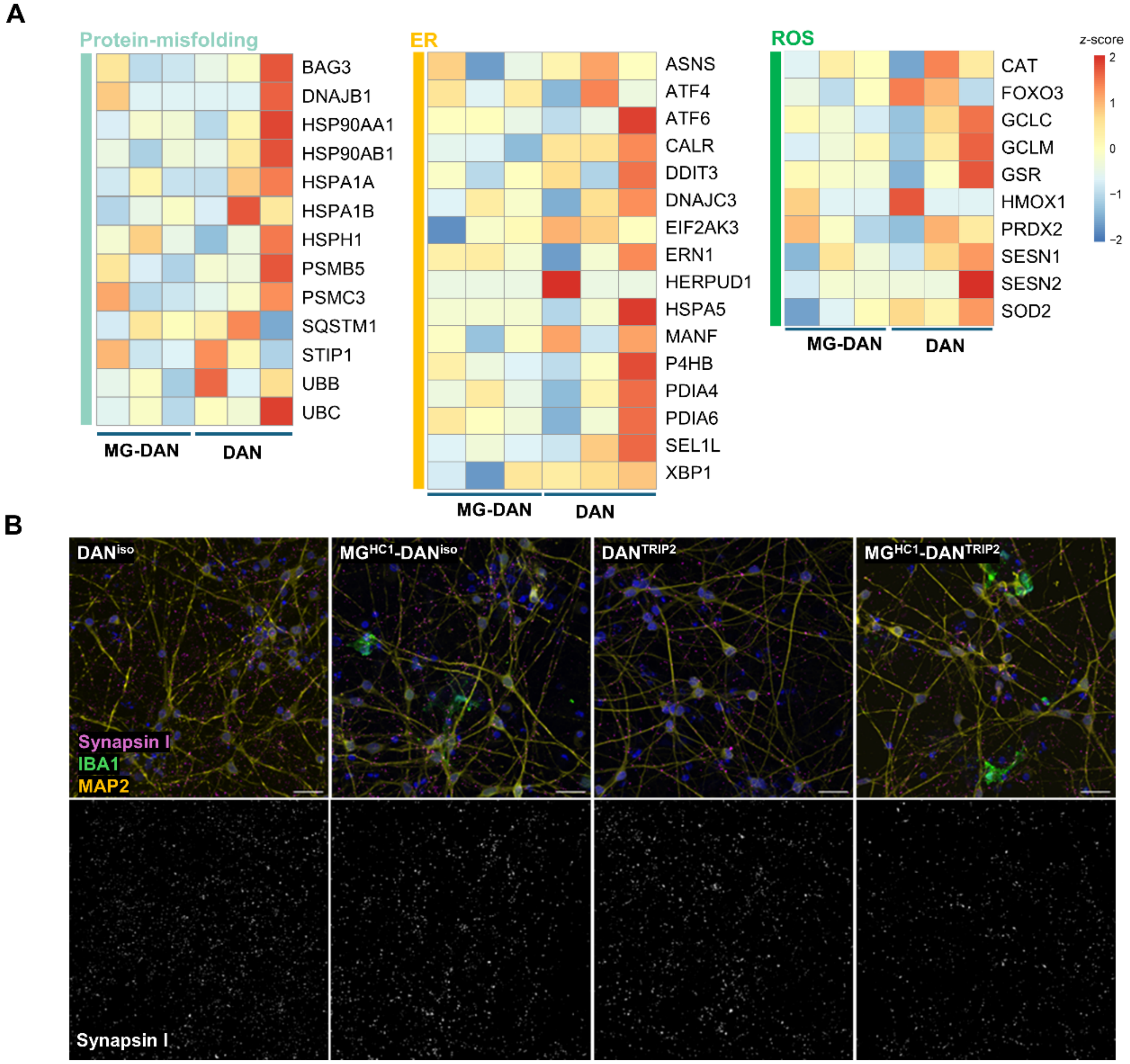

Fig. S2 Characterisation of iPSC-derived dopaminergic neurons and microglia in co-culture.

(A) Bulk RNA-seq heatmaps showing stress gene expression profile involving protein-misfolding, endoplasmic reticulum (ER), and inflammation in DAN^{TRIP} monocultures which is reduced in MG-DAN^{TRIP} co-cultures.

(B) Representative confocal images of Synapsin I^{+} pre-synaptic terminals in DAN^{TRIP2} or DAN^{ISO} monocultures and when co-cultured with IBA1^{+} microglia (MG). Note reduced Synapsin I^{+} puncta in DAN^{TRIP2} neurons in co-culture with MG. The representative image of MG^{HC1}-DAN^{TRIP2} was used for 3D reconstruction and is also shown in **Fig.1J.** Quantification is shown in **Fig. 1I.** *Scale bar*, 20 µm.

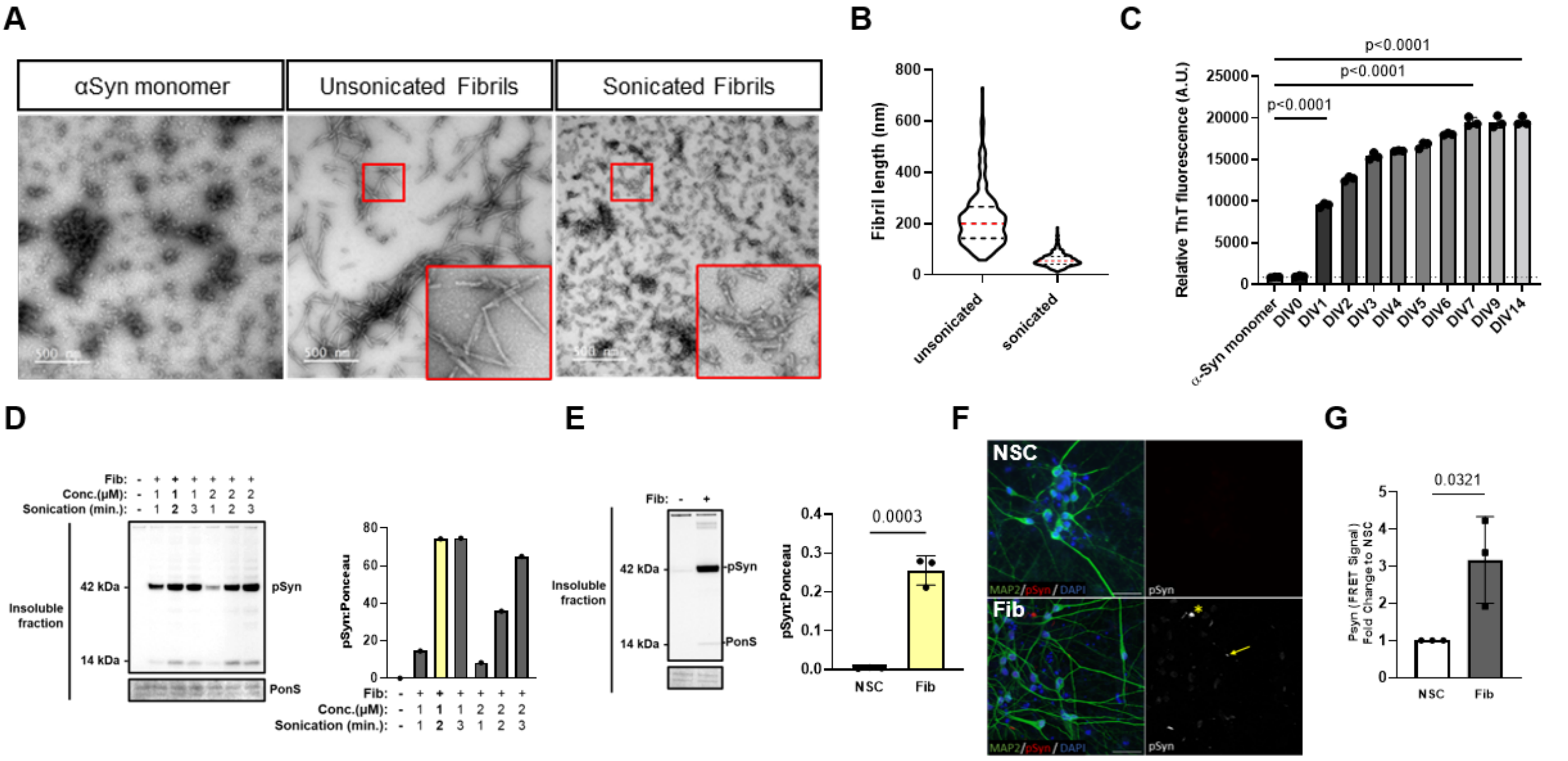

Fig. S3 *De novo* generated α-synuclein fibrils seed aggregation in HEK293 cells and iPSC-derived dopaminergic neurons.

(A) Electron micrographs depict α-synuclein (αSyn) monomers or αSyn fibrils after 7 days of shaking, and fragmented fibrils after sonication.

(B) Median Fibril length decreased from 200 nm to 55 nm after 2 min of sonication.

(C) Quantification of Thioflavin T(ThT) fluorescence showed that fibrils (Fib) unlike monomeric protein exhibit increased signal indicating assembly into β-sheet-rich structures (n=3 independent experiments).

(D) Immunoblotting and quantification for pSyn in the RIPA-insoluble fraction of HEK293 stably expressing α-Syn-venus after seeding overnight showing optimisation of fibril dose at different fibril sonication times.

(E) Immunoblotting and quantification of pSyn-venus confirmed the reproducibility of seeding with fibrils (1µM) at the optimal (2 minutes) sonication (n=3 independent experiments).

(F) Representative confocal images of DAN^{TRIP} treated with sonicated fibrils (Fib) for 2 weeks at 1µM stained positive for pSyn aggregates in processes (arrowheads) and cell bodies (asterisk), which are absent in the non-seeded condition (NSC). *Scale bar*, 20 µm.

(G) Time-resolved FRET assay using DAN lysates revealed increased pSyn levels 2 weeks post seeding with Fib compared to NSC (n=3 independent differentiations for DAN^{TRIP1}).

Data are presented as mean ± SD. Statistical analysis by one-way ANOVA **(C)** with Tukey's post hoc test, two-tailed t-test **(E)**, or one sample t-test **(G)**.

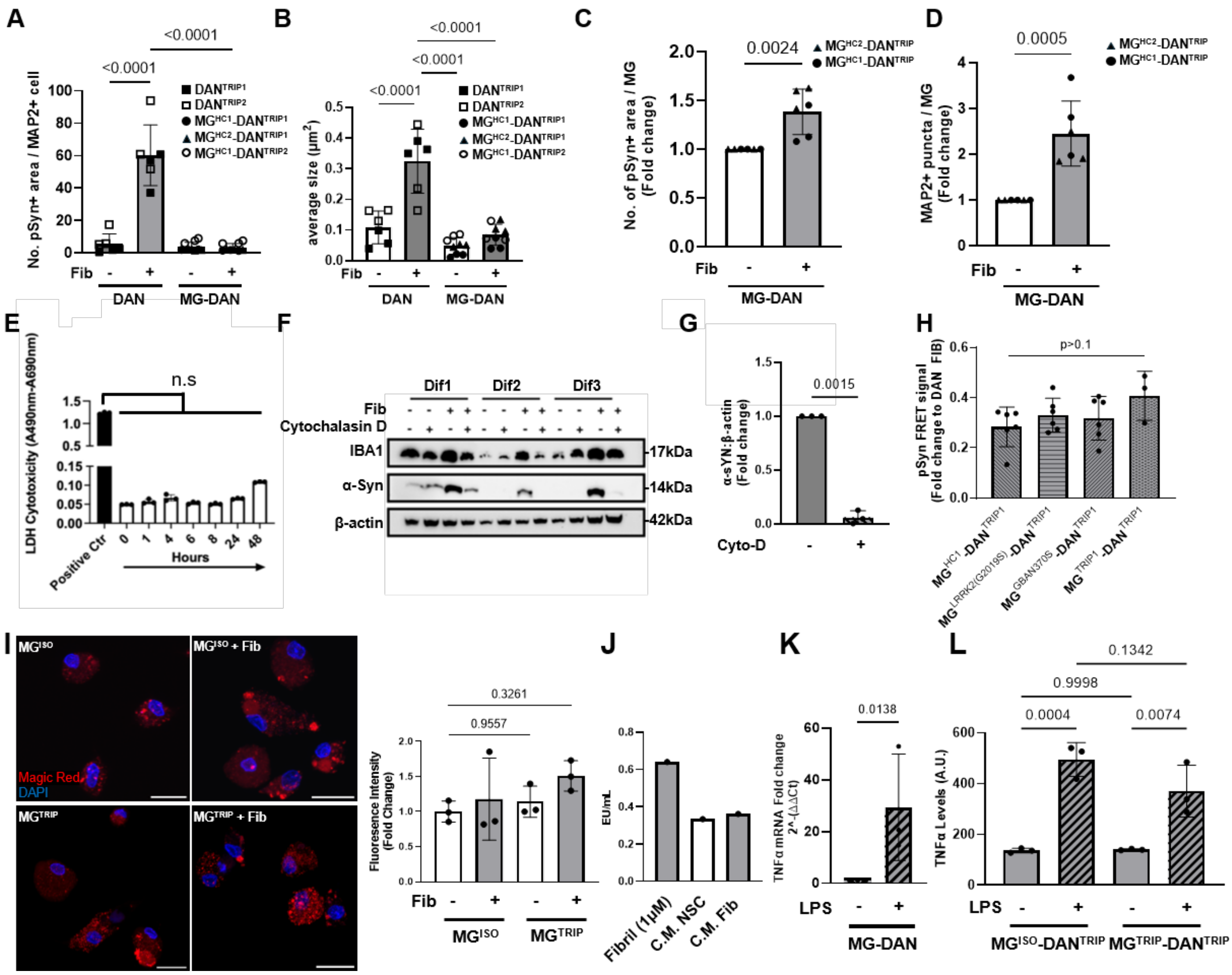

Fig. S4 Microglia remove large intraneuronal α-synuclein aggregates.

(A) Quantification of the total pSyn^{+} area count normalised to the number of MAP2^{+} neurons showing reduced pSyn aggregates in MG-DAN co-cultures compared to DAN monocultures under Fibril-seeded conditions (n = 3 independent differentiations for DAN^{TRIP1}, DAN^{TRIP2}, MG^{HC1}-DAN^{TRIP1}, MG^{HC2}-DAN^{TRIP1} and MG^{HC1}-DAN^{TRIP2}).

(B) Quantification of the total pSyn^{+} area size normalised to number MAP2^{+} neurons showing reduced pSyn aggregates in MG-DAN co-cultures compared to DAN monocultures under fibril-seeded conditions (n = 3 independent differentiations for DAN^{TRIP1}, DAN^{TRIP2}, MG^{HC1}-DAN^{TRIP1}, MG^{HC2}-DAN^{TRIP1} and MG^{HC1}-DAN^{TRIP2}).

(C) Quantification of pSyn^{+} area count within CD68^{+} MG (n = 3 independent differentiations for MG^{HC1}-DAN^{TRIP1} and MG^{HC2}-DAN^{TRIP1}).

(D) Quantification of MAP2^{+} area count within CD68^{+} MG (n = 3 independent differentiations for MG^{HC1}-DAN^{TRIP1} and MG^{HC2}-DAN^{TRIP1}).

(E) Time-course examination of 10 µM cytochalasin D revealed MG toxicity at 48 h but not at earlier time-points using LDH (Lactate Dehydrogenase) (n=3 independent differentiations for MG^{HC1}).

(F) αSyn fibril (1 µM) uptake by MG is blocked in cells treated with cytochalasin D (10 µM 1h prior to fibril addition and for 24h thereafter), compared to the fibril condition alone, shown using immunoblotting.

(G) Quantification of fibril uptake following inhibition of phagocytosis using cytochalasin D (n=3 independent differentiations for MG^{HC1}).

(H) HC, *GBA*^{N409S}, *LRRK2*^{G2019S}, or *SNCA*^{TRIP} mutant MG clear pSyn^{+} neuronal aggregates similarly when co-cultured with DAN under fibril-seeded conditions, measured using time-resolved FRET assay (n≥3 independent differentiations per MG iPSC line).

(I) Representative images and quantification of Cathepsin B activity using the Magic Red assay showing no change in fluorescent intensity between MG^{ISO} and MG^{TRIP} monocultures at baseline and when treated with fibrils (n=3 independent differentiations for MG^{ISO} and MG^{TRIP2}). *Scale bar*, 20 µm.

(J) Endotoxin levels in the conditioned media of fibril-seeded DAN monocultures before the addition of MG is less than 0.5 EU/ml and comparable to NSC DANs.

(K) qPCR analysis of TNFα mRNA in LPS-treated co-cultures vs baseline showing LPS-induced TNFα up-regulation (n=3 independent differentiations for MG^{ISO}-MG^{TRIP2}).

(L) TNFα levels do not differ between MG^{ISO} and MG^{TRIP} co-cultured with DAN^{TRIP} under basal or LPS-treated conditions (n=3 independnet differentiations for MG^{ISO}-DAN^{TRIP2} and MG^{TRIP2}-DAN^{TRIP2}).

Data are presented as mean ± SD. Statistical analysis by one-way ANOVA **(A, B, E, H, I, L)** with Tukey's post hoc test or one sample t-test **(C, D, G, K)**.

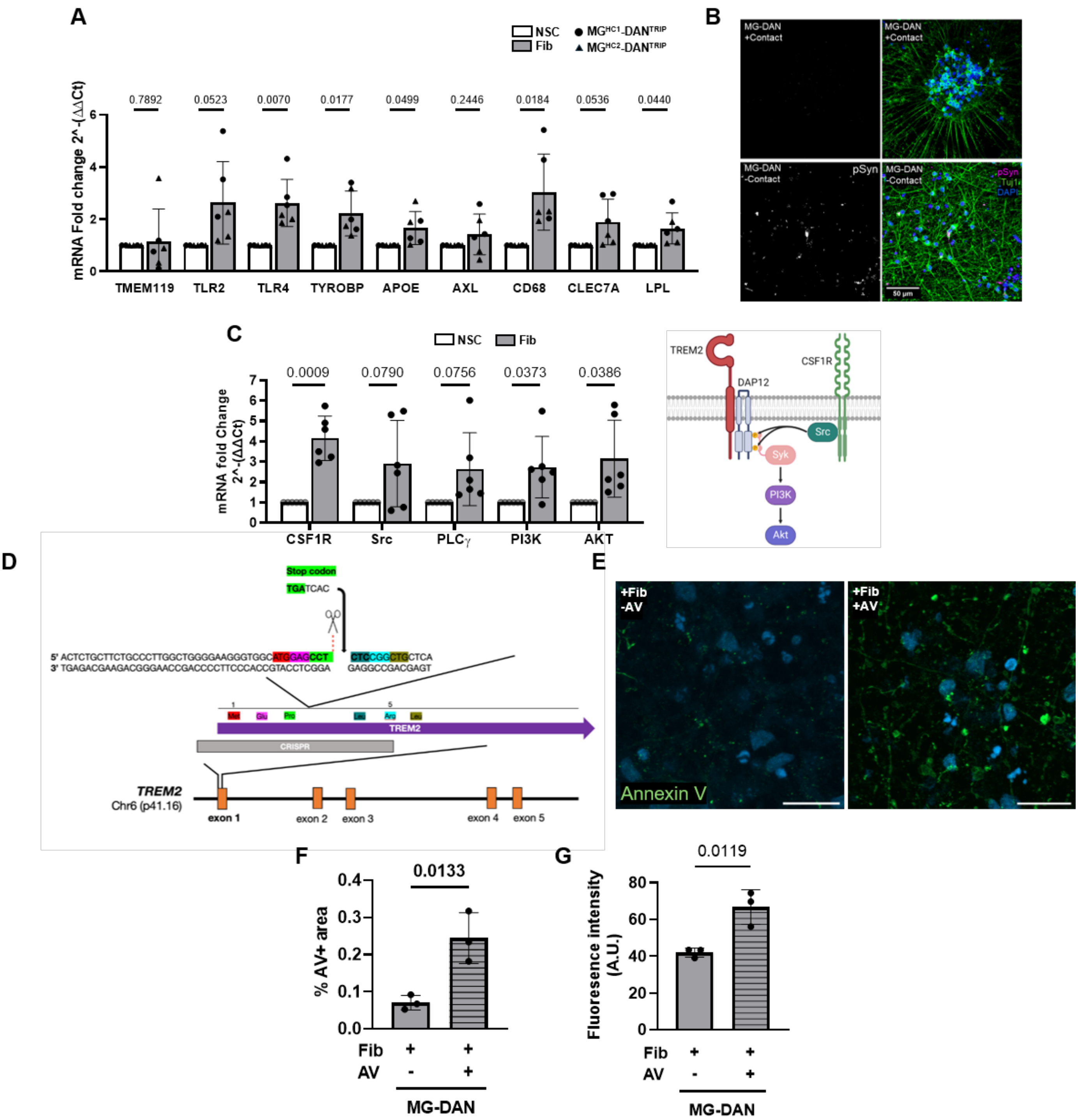

Fig. S5 Clearance of α-synuclein aggregates is TREM2-independent

(A) qPCR analysis of relevant markers (*TMEM119, TLR2, TLR4, TYROBP, APOE, AXL, CD68, CLEC7A, LPL*) normalised to GAPDH and plotted relative to the NSC condition showing up-regulation of DAM and no change in the homeostatic gene *TMEM119* (n=3 independent differentiations for MG^{HC1}-DAN^{TRIP1} and MG^{HC2}-DAN^{TRIP1}).

(B) Representative confocal images of pSyn staining in DAN^{TRIP} when MG^{HC1} were co-cultured with or without contact using a transwell under fibril-seeded conditions.

(C) qPCR analysis showing upregulation of genes involved in TREM2 signalling (*CSF1R*, *Src*, *PLCγ*, *PI3K*, and *AKT*) in the fibril-seeded condition compared to NSC in MG-DAN co-cultures (n=6 independent differentiations for MG^{HC1}-DAN^{TRIP1}).

(D) CRISPR/Cas9-mediated knockout of human TREM2. The 5' end of exon 1 was targeted using CRISPR/Cas9, introducing a TGA stop codon and four additional bases via homology-directed repair (HDR), resulting in a homozygous TREM2 knockout (BIONi010-C).

(E) Representative confocal images of Annexin V (AV) staining to confirm binding to and thus blocking of phosphatidylserine.

(F) Quantification of % AV^+ area normalised to number of nuclei (n=3 independent differentiations for MG^{HC1}-DAN^{TRIP1}). *Scale bar*, 20 µm

(G) Quantification of AV fluorescence intensity normalised to number of nuclei (n=3 independent differentiations for MG^{HC1}-DAN^{TRIP1}).

Data are presented as mean ± SD. Statistical analysis by one sample t-test **(A, C)** or two-tailed t-test **(F, G)**. *Scale bar*, 20 µm, unless else specified.

A

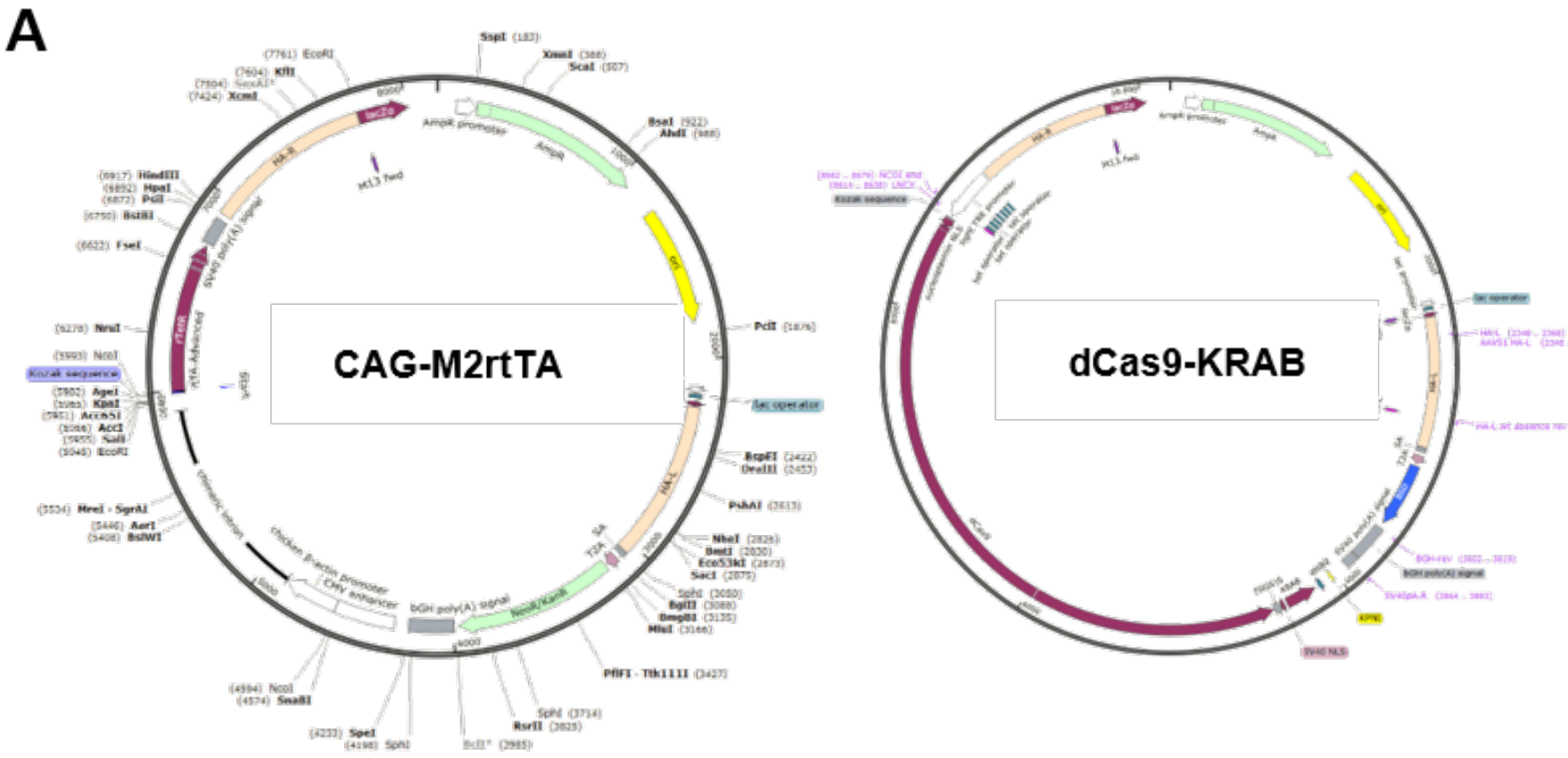

B

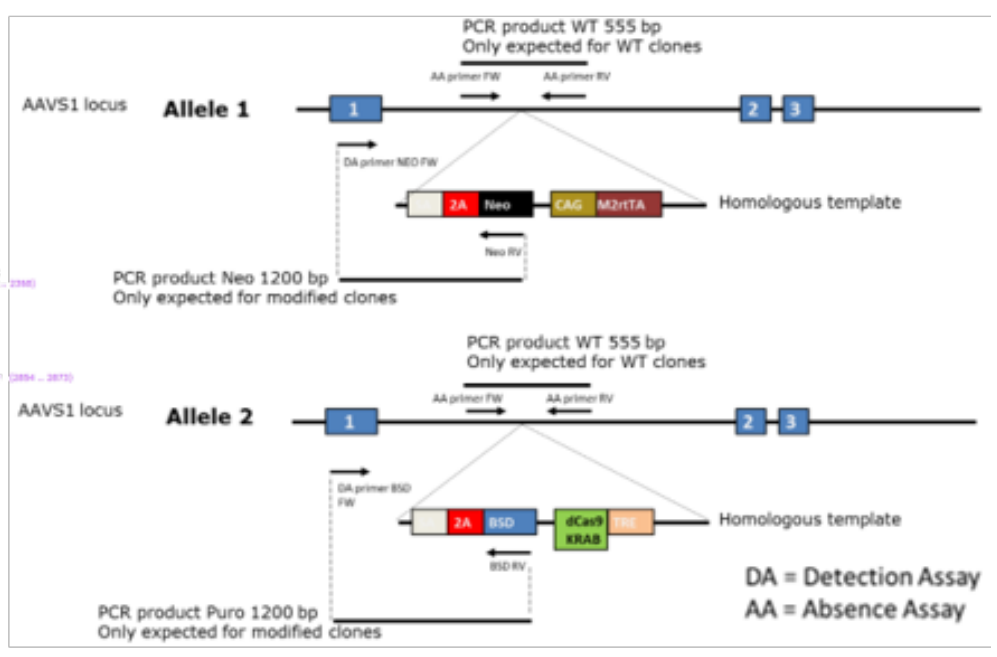

C

CRISPR	Sequence
CR2 AAVS1_rv	TGTCCCTAGTGGCCCCACTGTGG

Primer	Sequence	Product size
DA primer BSD_fw	CTCCGACGGATGTCTCCCTTGCG	1200
DA primer BSD_rv	CACGCTGTAATCCTCGCTGA	
DA primer NEO_fw	CCAGAGGCGGCGCAGAAGCCAG	1200
DA primer NEO_rv	ATCTTGTTCAATGGCCGATCCCAT	
AA primer_fw	GTCACCAGATAAGGATCTGCC	555
AA primer_rv	CTCCGACGGATGTCTCCCTTGCG	

D

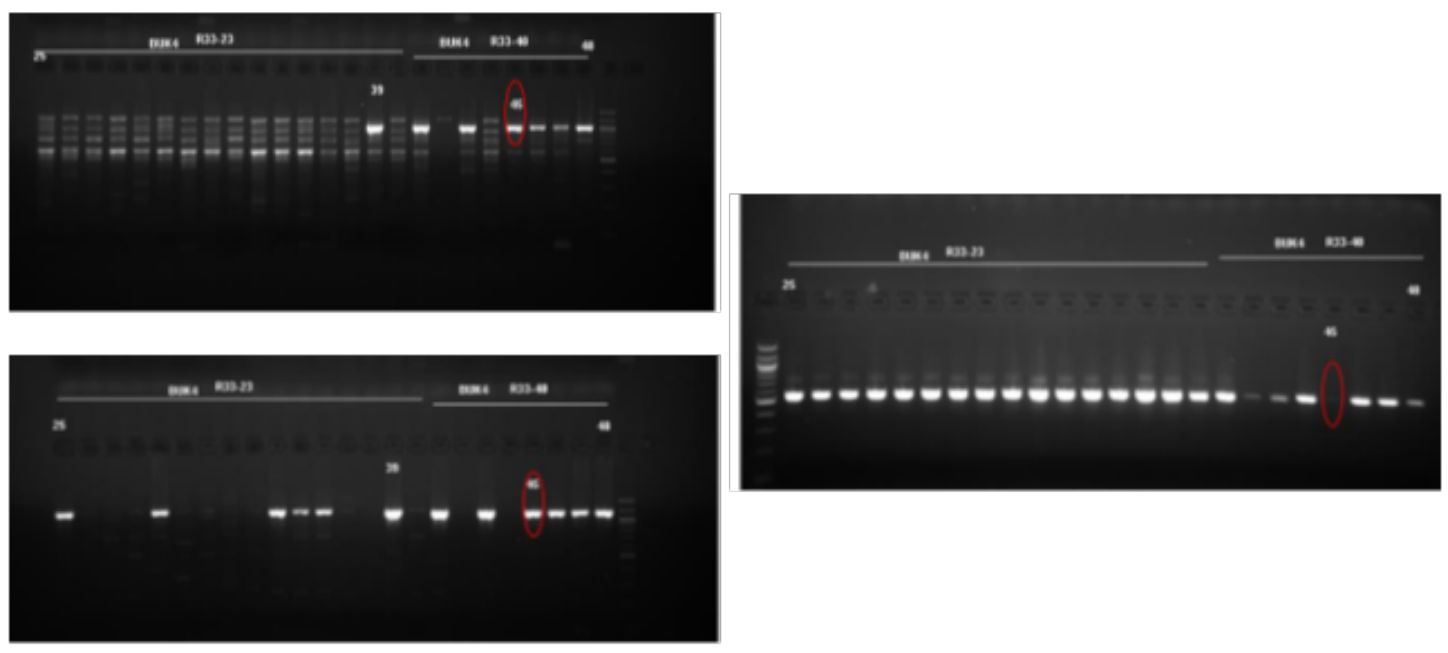

E

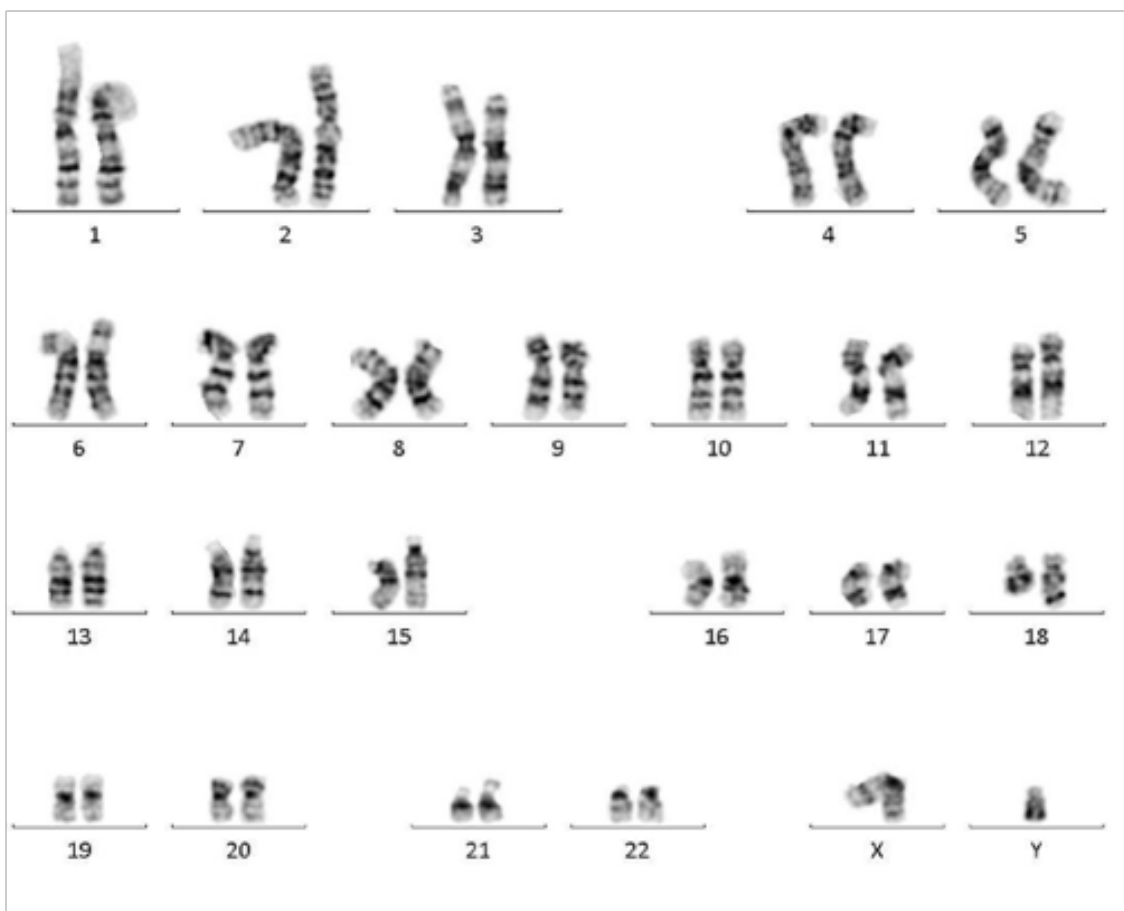

F

	HC1		dCas9-HC1	
Marker	Allele 1	Allele 2	Allele 1	Allele 2
AMEL	X	Y	X	Y
CSF1PO	10	12	10	12
D13S317	8		8	
D16S539	10	13	10	13
D18S51	15	16	15	16
D19S433	16		16	
D21S11	30	31,2	30	31,2
D2S1338	23	24	23	24
D3S1358	15	17	15	17
D5S818	11		11	
D7S820	8	11	8	11
D8S1179	11	15	11	15
FGA	20	21	20	21
TH01	9	9,3	9	9,3
TPOX	8		8	
vWA	15	18	15	18

G

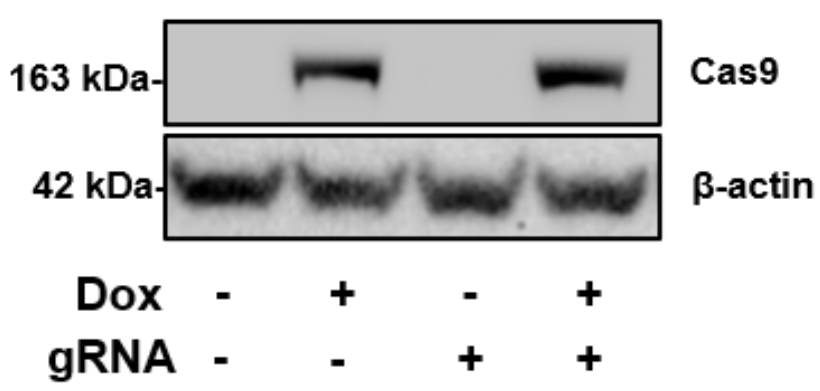

H

Gene	gRNA ID	TSS Site	Protospacer Sequence
P2RY12	P2RY12.1	P1P2	GTGTCCAGGGTCAGATTACA
P2RY12	P2RY12.2	P1P2	GGTGATCACAATCAGAAGAC
P2RY12	P2RY12.3	P1P2	GCTGAAATGACAAAAATCCA

I

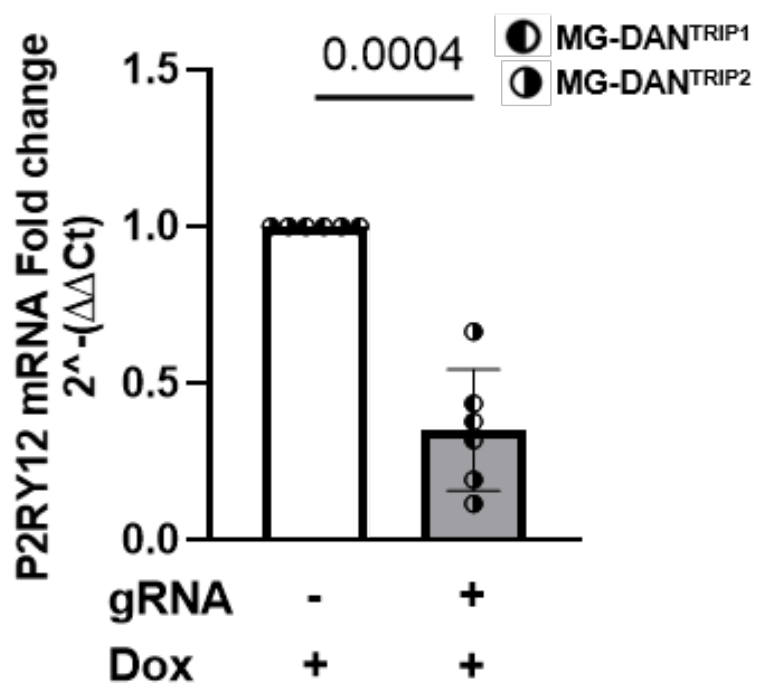

Fig S6. Generation of doxycycline inducible dCas9-KRAB gene in hiPSCs

(A) Plasmid maps of the reverse tetracycline-controlled transactivator (rtTA) and dCas9 sequences used to generate dead Cas9-KRAB hiPSCs using clone OX3-9 (HC1). iPSCs were nucleofected with the CRISPR “CR2_AAVS1_RV” and 2 µg of each plasmid.

(B) Schematic showing the integration sites of the rtTA and dCas9 sequences at the AAVS1 loci. 1, 2 and 3 represent the respective exon numbers in the AAVS1 locus. SA: splice acceptor, 2A: self-splicing protein, CAG: constitutively active promoter, TRE: tetracycline transactivator responsive element. Screening primers are shown as arrows.

(C) Table showing the primer sequences used to generate and confirm the doxycycline inducible dCas9 system in hiPSCs.

(D) PCR gels confirming insertion of the M2rtTA and the dCas9-KRAB expression cassettes into the AAVS1 locus of HC1 hiPSCs resulting in a doxycycline inducible dCas9 system.

(E) Karyotyping of the newly generated dCas9-KRAB HC1 hiPSCs.

(F) Short tandem repeat (STR) analysis of the dCas9-KRAB HC1 hiPSC clone and its corresponding line of origin. The number of allelic repeats tested in each locus matched between both lines, confirming that HC1/dCas9 clone was derived from the parent HC1 hiPSC line.

(G) Immunoblotting with anti-Cas9 antibody in iPSC lysates showing robust dCas9 induction upon doxycycline (Dox) treatment, without any expression observed in non-Dox treated controls.

(H) gRNA sequences obtained from the hCRISPRi-v2.1 Weissman Laboratory Library. The targeted promoter region at the transcription start site (TSS) is indicated.

(I) Quantification of P2RY12 mRNA level by qPCR confirmed P2RY12 knockdown in MG-DAN co-cultures compared to control MG (n = 3 independent differentiations for MG^{dCas9}-DAN^{TRIP1} and MG^{dCas9}-DAN^{TRIP2}).

Data are presented as mean ± SD. Statistical analysis by one sample t-test **(I)**.

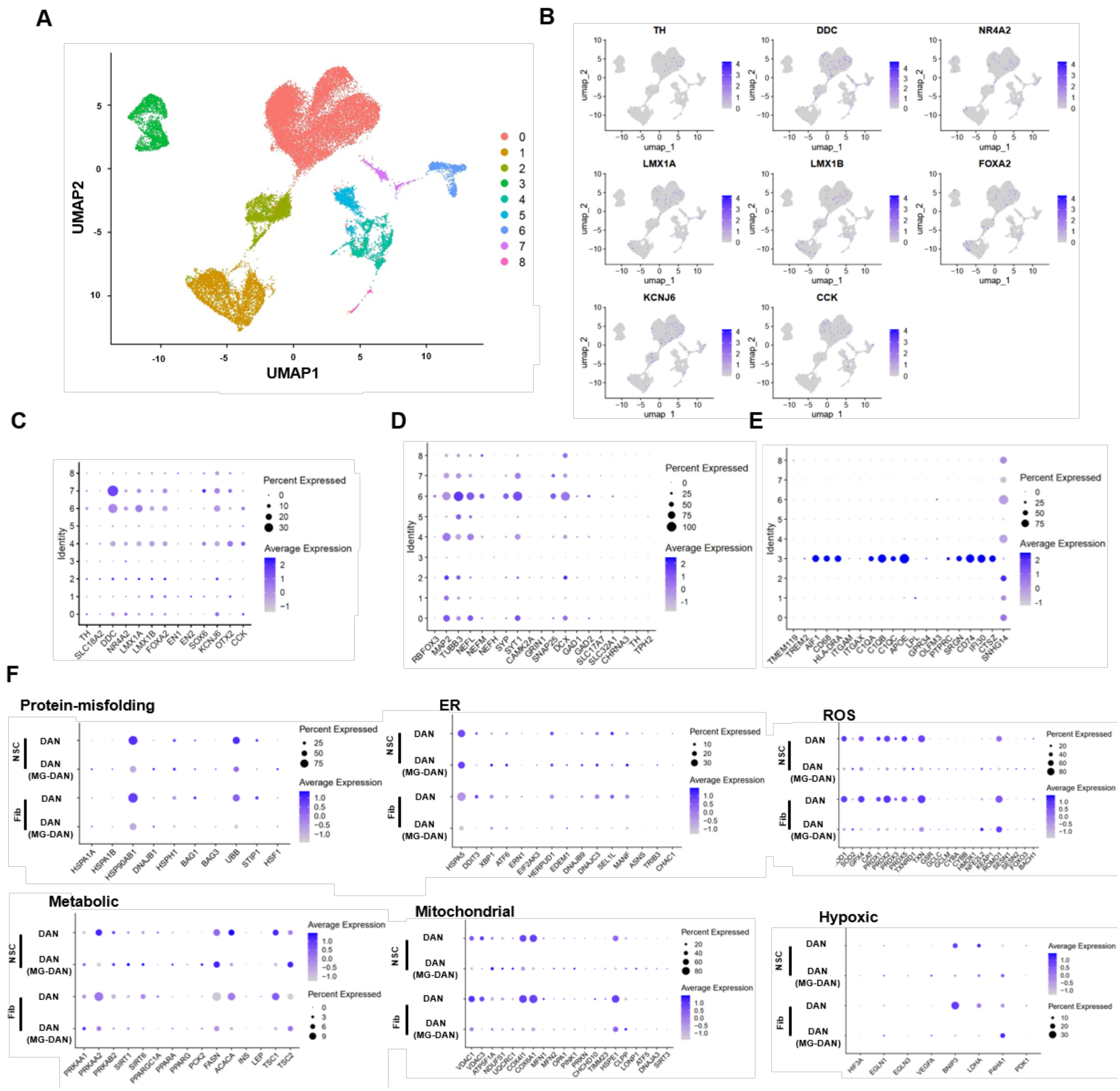

Fig S7 Single cell RNA Sequencing analysis for DANs

(A) UMAP of single-cell transcriptomes from MG–DAN co-cultures reveal neuronal (clusters 0, 4, 6, 7) and microglial (cluster 3) identities.

(B) Expression of DAN markers across cell clusters visualised by UMAP projection.

(C) Dot plot showing expression of DAN markers across clusters.

(D) Dot plot showing expression of neuronal markers in each cluster.

(E) Dot plot showing expression of MG markers across clusters.

(F) Dot plot showing reduced stress gene expression profile in DANs isolated from co-cultures compared to monocultures in relation to protein-misfolding, ER stress and reactive oxygen species (ROS), metabolic stress, mitochondrial stress and hypoxia. The strongest effect was observed under the fibril-seeded conditions.

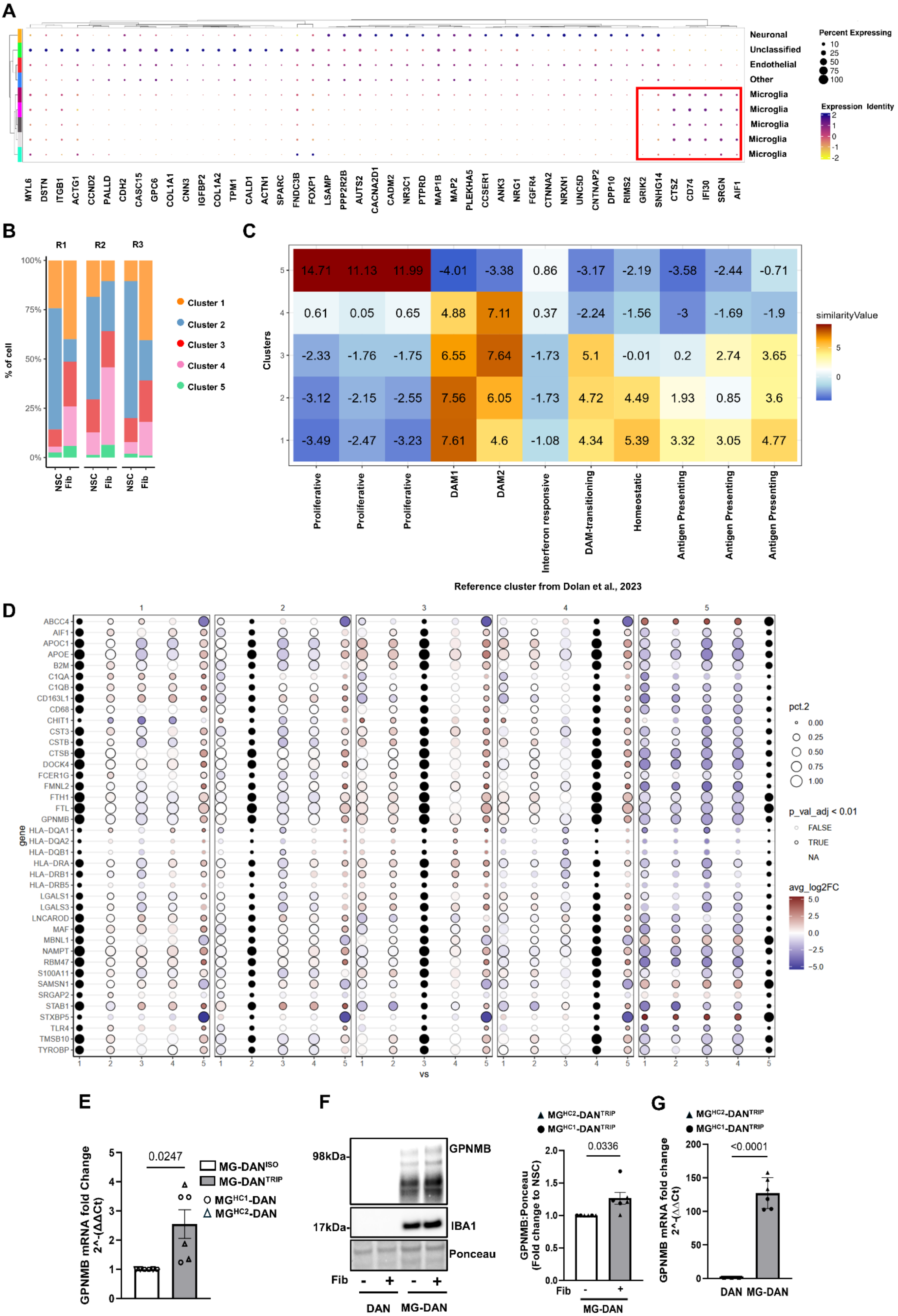
A
Neuronal
Unclassified
Endothelial
Other
Microglia
Microglia
Microglia
Microglia
Microglia
Percent Expressing
10
25
50
75
100
Expression Identity
2
1
0
-1
-2
MYL6
DSTN
ITGB1
ACTG1
CCND2
PALLD
CDH2
CASC15
GPC6
COL1A1
CNN3
IGFBP2
COL1A2
TPM1
CALD1
ACTN1
SPARC
FNDC3B
FOXP1
LSAMP
PPP2R2B
AUTS2
CACNA2D1
CADM2
NR3C1
PTPRD
MAP1B
MAP2
PLEKHA5
CCSER1
ANK3
NRG1
FGFR4
CTNNA2
NRXN1
UNC5D
CNTNAP2
DPP10
RIMS2
GRIK2
SNHG14
CTSZ
CD74
IFI30
SRGN
AIF1
B
R1
R2
R3
100%
75%
50%
25%
0%
% of cell
NSC
Fib
Cluster 1
Cluster 2
Cluster 3
Cluster 4
Cluster 5
C
Clusters
5: 14.71, 11.13, 11.99, -4.01, -3.38, 0.86, -3.17, -2.19, -3.58, -2.44, -0.71
4: 0.61, 0.05, 0.65, 4.88, 7.11, 0.37, -2.24, -1.56, -3, -1.69, -1.9
3: -2.33, -1.76, -1.75, 6.55, 7.64, -1.73, 5.1, -0.01, 0.2, 2.74, 3.65
2: -3.12, -2.15, -2.55, 7.56, 6.05, -1.73, 4.72, 4.49, 1.93, 0.85, 3.6
1: -3.49, -2.47, -3.23, 7.61, 4.6, -1.08, 4.34, 5.39, 3.32, 3.05, 4.77
Proliferative
Proliferative
Proliferative
DAM1
DAM2
Interferon responsive
DAM-transitioning
Homeostatic
Antigen Presenting
Antigen Presenting
Antigen Presenting
similarityValue
5
0
Reference cluster from Dolan et al., 2023
D
1
2
3
4
5
gene
ABCC4
AIF1
APOC1
APOE
B2M
C1QA
C1QB
CD163L1
CD68
CHIT1
CST3
CSTB
CTSB
DOCK4
FCER1G
FMNL2
FTH1
FTL
GPNMB
HLA-DQA1
HLA-DQA2
HLA-DQB1
HLA-DRA
HLA-DRB1
HLA-DRB5
LGALS1
LGALS3
LNCAROD
MAF
MBNL1
NAMPT
RBM47
S100A11
SAMSN1
SRGAP2
STAB1
STXBP5
TLR4
TMSB10
TYROBP
vs
pct.2
0.00
0.25
0.50
0.75
1.00
p_val_adj < 0.01
FALSE
TRUE
NA
avg_log2FC
5.0
2.5
0.0
-2.5
-5.0
E
0.0247
MG-DAN^ISO
MG-DAN^TRIP
MG^HC1-DAN
MG^HC2-DAN
GPNMB mRNA fold Change
2^-(ΔΔCt)
F
98kDa-
GPNMB
17kDa-
IBA1
Ponceau
Fib
DAN
MG-DAN
MG^HC2-DAN^TRIP
MG^HC1-DAN^TRIP
0.0336
GPNMB:Ponceau
(Fold change to NSC)
MG-DAN
G
MG^HC2-DAN^TRIP
MG^HC1-DAN^TRIP
<0.0001
GPNMB mRNA fold change
2^-(ΔΔCt)
DAN
MG-DAN

Fig S8. scRNA-seq reveals DAM Subtype mediating clearance of α-synuclein aggregates.

(A) Clustered dot plot showing marker expression across cell types, with microglia further grouped into subclusters

(B) Analysis of MG cluster composition across NSC and fibril-seeded conditions revealed a higher proportion of MG in clusters 3 and 4 under the fibril-seeded conditions.

(C) Similarity of MG clusters with published iPSC-derived human microglia states upon exposure to different brain substrates (Dolan et al., 2023) based on similarity scores (González-Velasco et al., 2024).

(D) Dot plot showing expression of MG cluster-defining genes with pairwise comparisons between clusters.

(E) qPCR analysis showing increased expression of *GPNMB* in MG co-cultured with DAN^{TRIP} compared to DAN^{ISO} normalized to GAPDH (n = 3 independent differentiations for MG^{HC1}-DAN^{ISO}, MG^{HC1}-DAN^{TRIP2} , MG^{HC2}-DAN^{ISO}, and MG^{HC2}-DAN^{TRIP2}).

(F) Immunoblotting confirmed increased expression of GPNMB in MG-DAN co-cultures under fibril-seeded conditions (n=3 independent differentiations for MG^{HC1}-DAN^{TRIP1} and MG^{HC2}-DAN^{TRIP1}).

(G) qPCR analysis shows that *GPNMB* is primarily expressed in MG-DAN co-cultures (n = 3 independent differentiations for MG^{HC1}-DAN^{TRIP1} and MG^{HC2}-DAN^{TRIP1}).

Data are presented as mean ± SD. Statistical analysis by one sample t-test **(E, F, G)**.

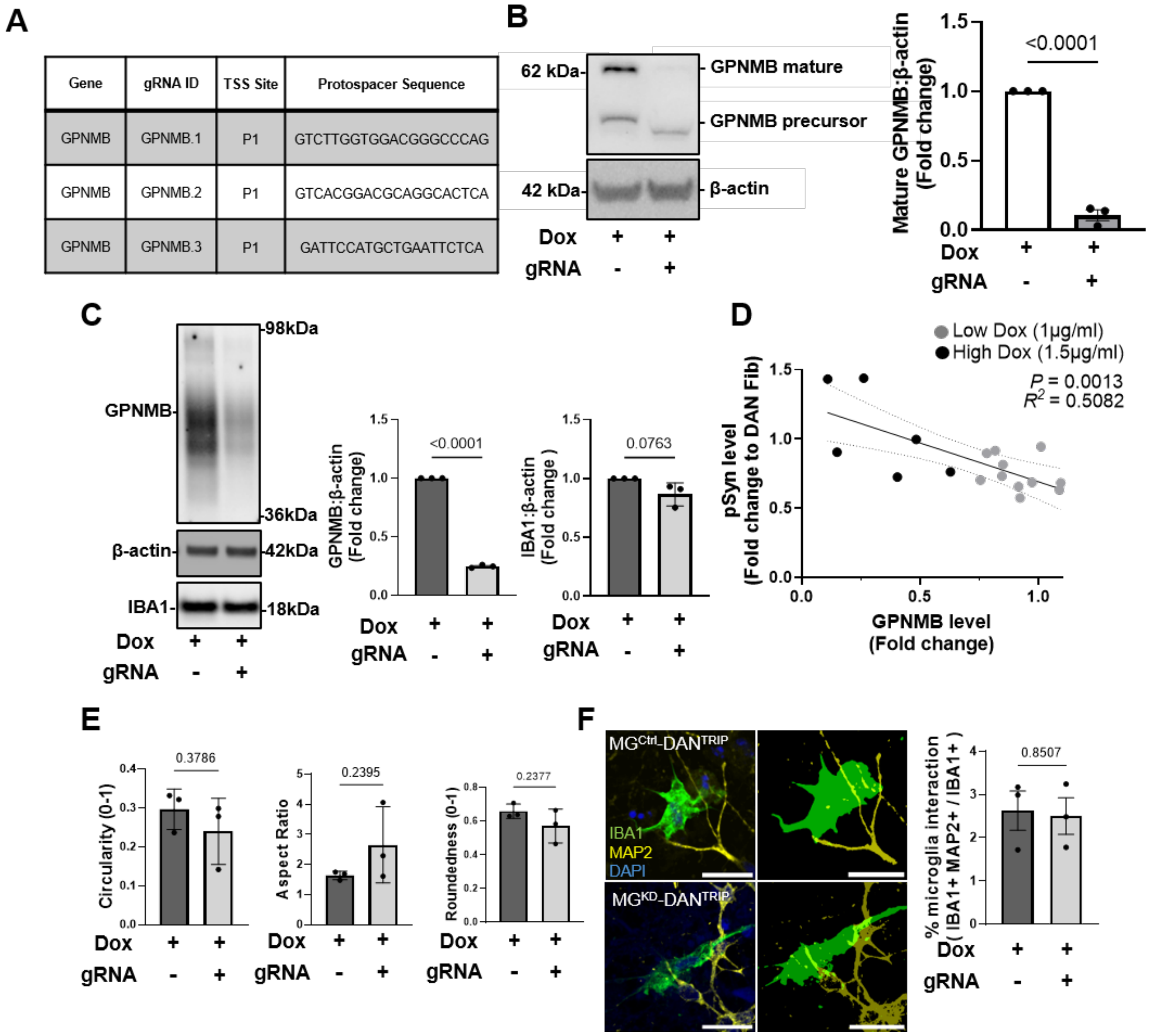

Fig. S9 Validation and characterization of GPNMB Knockdown

(A) gRNA sequences obtained from the hCRISPRi-v2.1 Weissman Laboratory Library. The targeted promoter region at the transcription start site (TSS) is indicated.

(B) Representative immunoblot and quantification for GPNMB in iPSC lysates showing 90% knockdown upon doxycycline treatment in transduced stem cells (n = 3 independent experiments for iPSC^{dCas9}).

(C) Representative immunoblot and quantification of GPNMB and IBA1 in MG monoculture lysates, showing a 75% knockdown of GPNMB upon doxycycline treatment in transduced MG compared to control, with no change in IBA1 expression (n = 3 independent experiments for MG^{dCas9}).

(D) Simple linear regression revealed a significant inverse relationship between relative GPNMB levels (fold change relative to control) and pSyn levels normalised to DAN Fib ($R^2 = 0.5082$, slope = −0.5605, $P = 0.0013$; n = 17 independent differentiations for MG^{dcas9}-DAN^{TRIP1}).

(E) Quantification of IBA1-masked MG for roundedness, circularity and aspect ratio under non-seeded (NSC) and fibril-seeded (Fib) conditions in MG-DAN^{TRIP} co-cultures with or without GPNMB knockdown (n=3 independent differentiations for MG^{dcas9}-DAN^{TRIP1}). *Scale bar*, 20 µm

(F) Representative confocal images and corresponding binary masks of MG^{dcas9}-DAN^{TRIP} co-cultures with or without GPNMB knockdown, showing IBA1/MAP2 overlap. Quantification was performed using Manders' coefficient to determine the percentage overlap of IBA1 with MAP2 in each image stack.(n = 3 independent differentiations for MG^{dcas9}-DAN^{TRIP1}).

The graphs show mean ± SD and was analysed by one sample t-test **(B， C)**, two-tailed t-test **(E, F)** and simple linear regression **(D).**

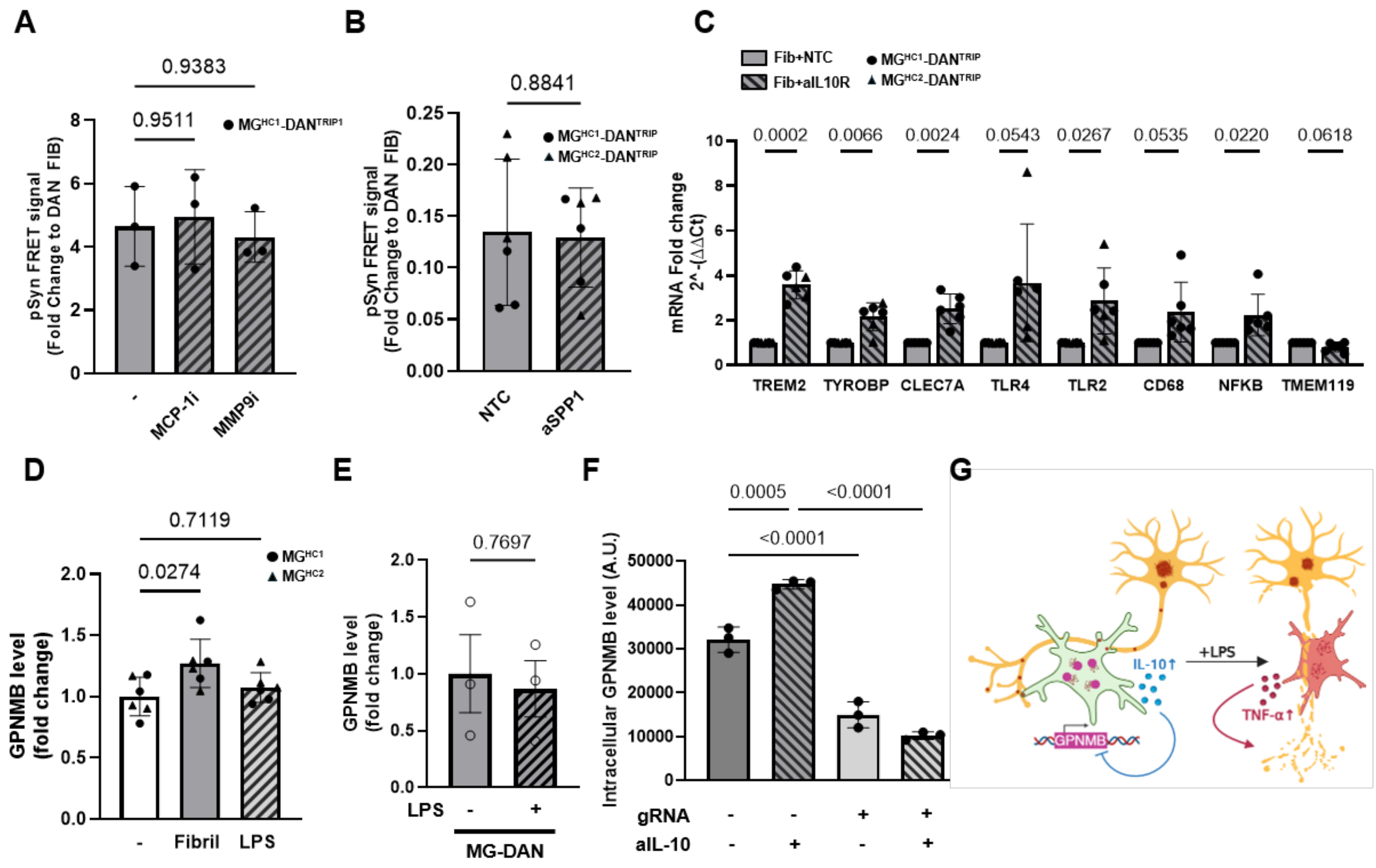

Fig S10. Autocrine IL-10 signalling limits microglia-mediated clearance of α-synuclein aggregates

(A) pSyn levels in MG-DAN did not change upon treatment of MCP-1 inhibitor (2µM) or MMP9 inhibitor (5µM), as quantified using time-resolved FRET (n = 3 independent differentiations for MG^{HC1}-DAN^{TRIP1}).

(B) pSyn levels in MG-DAN did not change upon treatment of anti-SPP1 antibody (5µg/mL) compared to NTC IgG (5µg/mL), as quantified using time-resolved FRET (n = 3 independent differentiations for MG^{HC1}-DAN^{TRIP1} and MG^{HC2}-DAN^{TRIP1}).

(C) qPCR analysis showed increased expression of DAM markers (*TREM2, TYROBP, CLEC7A, TLR4, TLR2, CD68, NFKB*), and no change in the homeostatic receptor TMEM119 in anti-IL-10R treated co-cultures versus NTC IgG (n= 3 independent differentiations for MG^{HC1}-DAN^{TRIP1} and MG^{HC2}-DAN^{TRIP1})

(D) GPNMB protein levels in MG monoculture lysates as quantified using ELISA are increased in response to fibril treatment, but not LPS (n = 3 independent differentiations for MG^{HC1} and MG^{HC2}).

(E) Quantification of GPNMB protein levels in MG-DAN co-culture lysates using ELISA reveals no increase upon LPS treatment in fibril- seeded conditions (n = 3 independent differentiations for MG^{ISO}-MG-DAN^{TRIP2}).

(F) Quantification of GPNMB protein levels in MG–DAN co-culture lysates using ELISA reveals up-regulation following anti-IL-10R treatment in control MG, but not in co-cultures with GPNMB KD MG (n = 3 independent differentiations for MG^{HC1}-DAN^{TRIP1}).

(G) Schematic diagram of the identified autocrine mechanism and effect of LPS.

Data are presented as mean ± SD. Statistical analysis by one sample t-test **(C)**, two-tailed t-test **(B, E)**, and one-way ANOVA **(A, D, F)** followed by Tukey's post hoc test.

ID	Diagnosis	genotype	Sex	Biopsy age (years)	Original ID	Reprogramming method	iPSC clone characterisation	GEO
HC1	HC	WT/WT	M	49	OX3-9	Cytotune1	Dafinca et al., 2016	GSE64584
HC2	HC	WT/WT	F	67	AH017-13<br>(STBCi026-A)	Cytotune1	Fernandes et al., 2016	GSE53426
$SNCA^{ISO}$	PD	Isogenic to $SNCA^{TRIP2}$	F	55	Clone 1-13	Cytotune1	Heman-Askah et al., 2017	GSE89886
$SNCA^{TRIP1}$	PD	Triplication/WT	F	55	SFC831-03-05<br>(STBCi024-C)	Cytotune1	Haenseler et al., 2017	GSE89886
$SNCA^{TRIP2}$	PD	Triplication/WT	F	55	ND34391G	Cytotune1	Heman-Askah et al., 2017	GSE89886
dCas9-HC1	HC	WT/WT	F	49	BUK4<br>(dCas9-OX 3-9)	Cytotune1	This study	N/A
$TREM2^{WT}$	HC	WT/WT	M	19	BIONi010-C	Cytotune 2	Hall-Roberts et al., 2020	GSE157635
$TREM2^{KO}$	HC	KO/KO	M	19	BIONi010-C-17	Cytotune 2	Hall-Roberts et al., 2020	GSE157635
LRRK2-1	PD	G2019S	F	57	MK144<br>(UOXFi008-A)	Retrovirus	Sandor et al., 2017	GSM2055807
LRRK2-2	PD	G2019S	M	57	SFC855-03-01<br>(STBCi007-C)	Cytotune 2	Booth et al., 2019	GSE120306
GBA-1	PD	N370S	M	72	SFC834-03-01<br>(STBCi025-A)	Cytotune 2	Fernandes et al., 2016	GSE53426
GBA-2	PD	N370S	M	72	SFC834-03-03<br>(STBCi025-B)	Cytotune 2	Fernandes et al., 2016	GSE53426

Table S1. Cell line information for all hiPSC lines used in this study. HC = healthy control, PD = Parkinson's disease.

ID	Age	Sex	Diagnosis	PMI (hours)	Brain weight (g)
CTRL 1	65	F	Normal	24	-
CTRL 2	72	F	Normal	72	1339
CTRL 3	59	M	Normal	24	1370
iLB1	88	M	Normal (Braak LB stage 5)	24	-
iLB2	77	F	Normal (Braak tangle stage 2)	34	-
iLB3	82	M	Normal (Braak LB stage 5)	29	1435
PD1	72	M	PD (Braak stage 6)	42	-
PD2	81	M	PD (Braak stage 6)	37	-
PD3	80	F	PD (Braak stage 6)	-	-

Table S6. Patient information for post-mortem brain tissue used in this study. PMI=post-mortem interval.

Antibodies		
Mouse anti-Tuj1 (monoclonal), 1:1000	BioLegend	Cat# 801201 [TUJ1], RRID: AB_2313773
Rabbit anti-alpha-synuclein (phospho S129) (monoclonal), 1:1000	Abcam	Cat# ab51253 [EP1536Y], RRID: AB_869973
Chicken anti-MAP2 (polyclonal), 1:1000	Abcam	Cat# ab92434, RRID: AB_2138147
Mouse anti-β-Actin (monoclonal), 1:5000	Sigma	Cat# A1978 [AC-15], RRID: AB_476692
Rabbit anti-tyrosine hydroxylase (monoclonal), 1:500	Cell Signaling Technology	Cat# 58844 [E2L6M], RRID: AB_2744555
Goat anti-IBA1 (polyclonal), 1:500	Abcam	Cat#ab5076 RRID: AB_2224402
Rabbit anti-IBA1 (polyclonal) 1:1000	FUJIFILM Wako Chemicals	Cat#019-19741 RRID: AB_839504
Mouse anti-CD68 (monoclonal) 1:500	Invitrogen	Cat # 14-0688-82 RRID: AB_11151139
Mouse anti-Osteoactivin/GPNMB (polyclonal) 1:500	Bio-Techne	Cat #AF2330 RRID: AB_2112934
Goat anti-Osteoactivin/GPNMB (polyclonal)1:500	Bio-Techne	Cat #AF2550 RRID: AB_416615
Mouse anti-CD22 (Clone 219903) 5μM	Bio-Techne	Cat#MAB19682 RRID: AB_3532205
Moust anti-IL10R Alpha 1μM	Bio-Techne	Cat#MAB274 RRID: AB_358187
anti-human osteopontin (SPP1) 5ug	Bio X cell	Cat#BE0382 RRID: AB_2927519
Mouse IgG_1 Isotype Control	Bio-Techne	Cat#MAB002 RRID: AB_357344
TREM2 Antaognist 10μg/ml	Eli Lilly	
IgG Control	Eli Lilly	
Goat anti-Mouse Alexa Fluor™ 488-conjugate, 1:1000	Invitrogen	Cat# A-11001, RRID: AB_2534069
Goat anti-Rabbit Alexa Fluor™ 488-conjugate, 1:1000	Invitrogen	Cat# A-11034, RRID: AB_2576217
Goat anti-Mouse Alexa Fluor™ 568-conjugate, 1:1000	Invitrogen	Cat# A-11031, RRID:AB_144696
Goat anti-Rabbit Alexa Fluor™ 568-conjugate, 1:1000	Invitrogen	Cat# A-11036, RRID: AB_10563566
Goat anti-Chicken Alexa Fluor™ 647-conjugate, 1:1000	Invitrogen	Cat# A-21449, RRID: AB_2535866
Donkey anti-Goat Alexa Fluor™ Plus 405-conjugate, 1:250	Invitrogen	Cat# A48259 RRID: AB_2890272
Donkey anti-Chicken Alexa Fluor™ 647-conjugate, 1:1000	Invitrogen	Cat#A78952 RRID: AB_2921074
Donkey anti-Rabbit Alexa Fluor™ 568-conjugate,1:1000	Invitrogen	Cat#A10042 RRID: AB_2534017
Donkey anti-Mouse Alexa Fluor™ 488-conjugate, 1:1000	Invitrogen	Cat#A21202 RRID: AB_141607

Table S7. Information for all antibodies used for blocking, immunocytochemistry and immunoblotting in this study.

Gene	Forward sequence (5' to 3')	Reverse sequence (3' to 5')
APOE	GTTGCTGGTCACATTCCTGG	GCAGGTAATCCCAAAAGCGAC
AXL	ATCAGCTTCGGCTAGGCAG	TCCGCGTAGCACTAATGTTCT
CD22	GCACCCTGAAACCCTCTACG	ATCAAACTTCGAGGTGTTCTTGT
CD68	CTTCTCTCATTCCCCTATGGACA	GAAGGACACATTGTACTCCACC
CHIT1	GGGCTCTGCTGCAAAACTG	AGAGTCTCGTCATTCCACTCAG
CLEC7A	GTCCTGGGTACCATGGCTATT	GTGTGGGTTGACTGTGGTTC
C1QA	GTGACACATGCTCTAAGAAG	GACTCTTAAGCACTGGATTG
CSF1R	GGGAATCCCAGTGATAGAGCC	TTGGAAGGTAGCGTTGTTGGT
GAPDH	TGGAAGGACTCATGACCACAG	CAGTGGGGACACGGAAGG
GAS6	CGAAGAAACTCAAGAAGCAG	AGACCTTGATCTCCATTAGG
GPNMB	CTTCTGCTTACATGAGGGAGC	GGCTGGTGAGTCACTGGTC
GPR34	GAAGACAATGAGAAGTCATACC	TGTTGCTGAGAAGTTTTGTG
LGALS3	ATGGCAGACAATTTTTCGCTCC	GCCTGTCCAGGATAAGCCC
LPL	AGGATGTGGCCCGGTTTATC	CCAAGGCTGTATCCCAAGAGAT
MERTK	AGGACTTCCTCACTTTACTAAG	TGAACCCAGAAAATGTTGAC
NFKB1	GAAGCACGAATGACAGAGGC	GCTTGGCGGATTAGCTCTTTT
P2RY12	AAGAGCACTCAAGACTTTAC	GGGTTTGAATGTATCCAGTAAG
PROS1	AAAGATGTGGATGAATGCTC	TCACATTCAAAATCTCCTGG
TLR2	AGTTGTGGGTTGAAGCACTG	TGGAGAGGCTGATGATGACC
TLR4	GCGTGGAGGTGGTTCCTAAT	GAGGTGGCTTAGGCTCTGAT
TNF-α	CAAAGTAGACCTGCCCAGAC	GACCTCTCTCTAATCAGCCC
TREM2	TCTGAGAGCTTCGAGGATGC	GGGGATTTCTCCTTCAAGA
TYROBP	ACTGAGACCGAGTCGCCTTAT	ATACGGCCTCTGTGTGTTGAG

Table S8. Primer sequences used for RT-qPCR.
